# Studies on a Urea-Complexed Iron(III) Dichromate, a Precursor of Chromium-Rich Nanospinel Catalysts Prepared for the Reductive Transformation of Carbon Dioxide

**DOI:** 10.1021/acs.inorgchem.4c05009

**Published:** 2025-02-10

**Authors:** Kende Attila Béres, Zoltán Homonnay, Laura Bereczki, Vladimir M. Petruševski, Attila Farkas, Zsuzsanna Czégény, Péter Németh, Péter Pekker, Fanni Béres-Szilágyi, Tomáš Stryšovský, Libor Kvitek, Ágnes Gömöry, László Kótai

**Affiliations:** † Institute of Materials and Environmental Chemistry, HUN-REN Research Centre for Natural Sciences, H-1117 Budapest, Hungary; ‡ György Hevesy PhD School of Chemistry, ELTE Eötvös Loránd University, H-1053 Budapest, Hungary; § Institute of Chemistry, ELTE Eötvös Loránd University, H-1053 Budapest, Hungary; ∥ Centre for Structural Science, HUN-REN Research Centre for Natural Sciences, H-1117 Budapest, Hungary; ⊥ Institute of Chemistry, Faculty of Natural Sciences and Mathematics, Ss. Cyril and Methodius University, Skopje MK-1000, Republic of North Macedonia; # Department of Organic Chemistry and Technology, Faculty of Chemical Technology and Biotechnology, Budapest University of Technology and Economics, H-1111 Budapest, Hungary; 7 Institute for Geological and Geochemical Research, HUN-REN Research Centre for Astronomy and Earth Sciences (MTA Centre of Excellence), H-1112 Budapest, Hungary; 8 University of Pannonia, Research Institute of Biomolecular and Chemical Engineering, Nanolab, H-8200 Veszprém, Hungary; 9 Department of Development, Bay Zoltán Nonprofit Ltd. for Applied Research, H-1116 Budapest, Hungary; 10 Faculty of Science, Department of Physical Chemistry, Palacky University Olomouc, Olomouc 77146, Czech Republic; 11 Institute of Organic Chemistry, MS Proteomics Research Group, HUN-REN Research Centre for Natural Sciences, H-1117 Budapest, Hungary

## Abstract

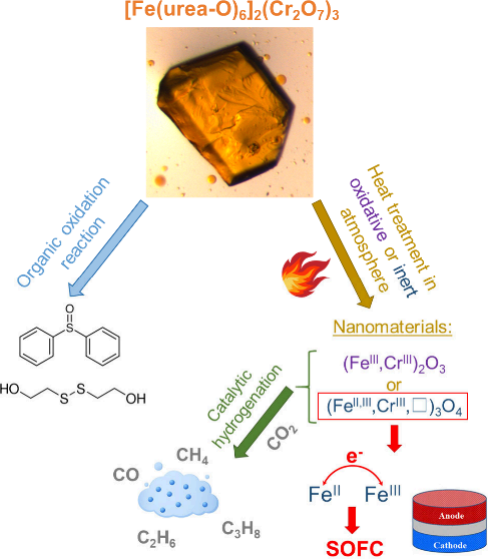

Energy-saving and cost-efficient reaction routes to prepare highly active catalysts for CO_2_ hydrogenation or solid oxide fuel cells (SOFCs) are enormously important. In this paper, we report a detailed study of a dichromate salt of [Fe(urea)_6_]^3+^, a member of the [*M*(urea)_6_]^3+^ complex family (*M* = Fe, Al, Mn, Cr, V, or Ti) with oxidizing anions, which is a promising precursor of a Cr-rich mixed chromium iron oxide catalyst prepared at a low temperature in the solid phase. The single-crystal X-ray structure, various (infrared, ultraviolet–visible, and Raman) spectroscopic studies, and thermal analysis (differential scanning calorimetry and thermogravimetric analysis/mass spectrometry) of [hexakis(urea-*O*)iron(III)] dichromate {[Fe(urea-O)_6_]_2_(Cr_2_O_7_)_3_} and its decomposition products confirmed the presence of a quasi-intramolecular redox reaction between the urea ligands and dichromate anions. The redox reactions result in various mixed Cr–Fe oxides with amorphous structure, whereas above 550 °C, the crystal structure and composition of the final products depend on the atmosphere during the thermal decomposition. The iron–chromium mixed oxides are potential catalysts in CO_2_ hydrogenation that afford CO, CH_4_, C_2_H_6_, and C_3_H_8_. Furthermore, our Mössbauer spectroscopy studies show a possible electron hopping between the Fe^II^ and Fe^III^ ions at the tetrahedral sites of the spinel structure, which suggests that the formed chromite is also a potential SOFC material. Our study also demonstrates that hexaureairon(III) dichromate is a selective oxidation agent of sulfur-containing organic compounds.

## Abstract 2

We performed studies on [hexakis(urea-*O*)iron(III)] dichromate {[Fe(urea-O)_6_]_2_(Cr_2_O7)_3_} with single-crystal X-ray structure and various spectroscopic (infrared, ultraviolet−visible, Raman, and Mössbauer) and thermal analysis (differential scanning calorimetry and thermogravimetric analysis/mass spectrometry) methods. A quasi-intramolecular redox reaction has occurred between the urea ligands and dichromate anions upon heating, which resulted in various nanosized mixed Cr−Fe oxides. Their phase relations depended on the atmosphere and the temperature of the heat treatment, and they showed catalytic activity in CO_2_ hydrogenation. Additionally, the spinel-like Cr−Fe oxide showed the presence of possible electron hopping between the Fe^II^ and Fe^III^ ions located at the tetrahedral spinel sites, which opens additional possibilities to use this material in solid oxide fuel cells.

## Introduction

The enormous importance of carbon dioxide reduction/transformation to decrease the global greenhouse effect and produce gaseous and liquid hydrocarbon fuels and oxygenated chemicals^1^ has initiated intensive research on developing new and efficient catalysts.^2^ Numerous methods and catalysts have been developed for the relevant synthesis technologies, including electrochemical, homogeneous, and heterogeneous reactions, with the formation of hydrocarbons, alcohols, and other chemicals.^3−12^ Carbon monoxide may also be hydrogenated (Fischer–Tropsch) into hydrocarbons using mixed transition metal oxides.^13^ The spinel catalysts can be prepared via various reaction routes, including sol–gel syntheses, oxidation of metallic alloys, co-precipitation reactions, etc.; however, these reaction routes result in various catalytic properties of the spinels depending on the starting materials, reaction type, composition, and synthesis conditions.^14−16^ Because the simple iron oxide catalysts convert into iron carbide during the catalytic reaction, stabilizers like potassium,^17^ manganese,^18^ and chromium^16^ are needed. In all of these cases, the level of conversion of CO_2_ was found to be ∼40%. To synthesize new catalysts for both CO_2_ and CO reduction and/or hydrogenation, we have prepared various metal complexes with reducing ligands like ammonia, pyridine, and urea and with permanganate anions.^13,19−21^ The solvent-mediated isothermal decomposition reactions led to various amorphous and nanosized mixed metal–manganese oxides, which are candidate catalysts for numerous reactions.^13,19−21^ In analogy to permanganate complexes, it is expected that the solid salts with complex chromate-containing cations and reducing ligands presumably also participate in redox reactions. The catalysts containing Fe and Cr oxides are potential materials in technologies such as CO and CO_2_ hydrogenation,^22,23^ conversion of carbon monoxide into fuels (Fischer–Tropsch^24,25^), and additional organic materials.^26−28^ In addition, the Cr doping of transition metal oxides is highly promising in the preparation of solid oxide fuel cells (SOFCs).^29−33^ Therefore, we launched a systematic study of various iron complex precursors containing chromate–dichromate anions and various reducing ligands.

**Figure 1:**
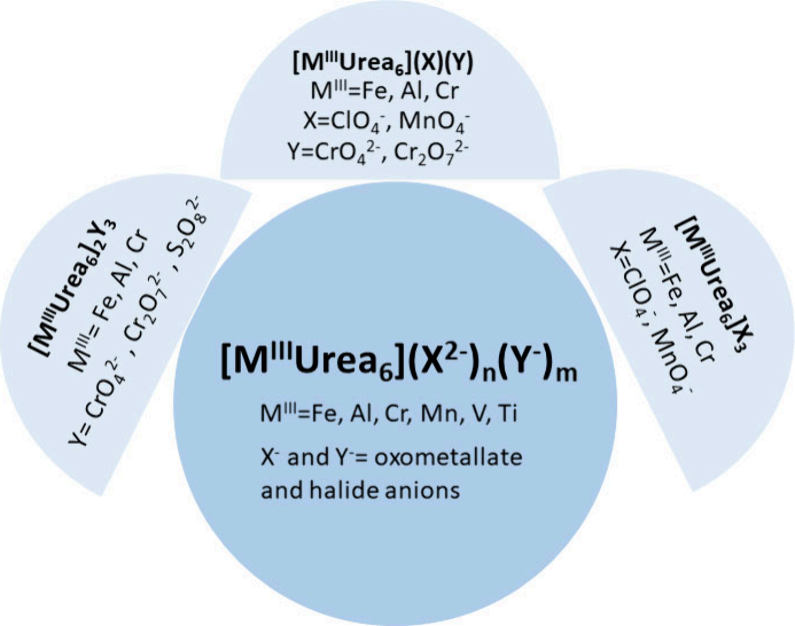
Diversity of the affordable mixed oxide catalyst precursors based on hexaurea complexes of trivalent metals with various metal- or non-metal-containing oxoanions.

Here we chose iron(III)-urea cation-containing complexes as the subject of our study. The [Fe(urea)_6_]^3+^ cations form a series of normal and mixed anionic salts with chromate and dichromate anions, including [Fe(urea)_6_]_2_(CrO_4_) and [Fe(urea)_6_]_2_(Cr_2_O_7_)_3_ (with iron:chromium stoichiometries 1:1.5 and 1:3, respectively). Members of the [*M*^III^(urea)_6_] complex family are summarized in Figure 1. These precursor compounds have been successfully synthesized but have not been characterized in detail.^34,35^ Partial substitutions of CrO_4_^2–^ and Cr_2_O_7_^2–^ anions by other divalent anions like SO_4_^2–^, S_2_O_7_^2–^, and S_2_O_8_^2–^ (which decompose into gaseous products during thermal treatment) ensure advantageous routes to synthesize potential catalysts with non-integer Fe:Cr ratios and with higher than expected Fe:Cr ratios for the nonsubstituted chromate or dichromate compounds. The isomorphism of analogue [Fe(urea)_6_]^3+^ and other [*M*(urea)_6_]^3+^ (*M* = Al, Mn, Cr, V, or Ti) complexes^35−41^ allows the preparation of limited (sometimes unlimited) solid solutions of precursor compounds from [(Fe, Cr, *M*)(urea)_6_](CrO_4_, Cr_2_O_7_, SO_4_, S_2_O_7_, S_2_O_7_, *Z*_2_O_8_)(*X*, *Y*) salts for a desired final Fe:Cr stoichiometry. Fine-tuning the Fe:Cr ratio by introducing a cationic (*M* = Cr^III^) and an anionic (Cr^VI^ in chromate or dichromate) chromium source into the precursor can be expected to influence the catalytic properties of the mixed oxide product. The variable valences of metal ions in the spinel lattices might initiate kinds electron hopping processes, which are highly likely possibilities to catalyze processes based on reversible redox interactions.^42,43^

This new way of preparing catalyst precursors may result in an excellent yield of spinels with specific properties and allows the use of fewer chemicals and less rigorous conditions compared to those of other available methods.^14,35,44^

If the anions X contain metallic elements (permanganate and perrhenate) and Y decomposes into gases (e.g., perchlorate), a series of ternary/doped Fe–Cr oxides might be prepared with various stoichiometries. This system opens virtually infinite possibilities to prepare an Al_2_O_3_/TiO_2_ carrier or supported doped/undoped Fe–Cr solid oxides.

As the initial step of this project, we prepared a simple compound, hexakis(urea-*O*)iron(III) dichromate (2:3), [Fe(urea)_6_]_2_(Cr_2_O_7_)_3_ (compound **1**), to demonstrate its decomposition into iron–chromium mixed oxide(s) with a nominal Fe to Cr ratio of 1:3, and studied its phase relations and catalytic activity in CO_2_ hydrogenation. We chose compound **1** because (1) data are mostly available for the iron-rich FeCr_2_O_4_–FeFe_2_O_4_ solid solutions and (2) the successful preparation and characterization of compound **1** can help prepare and study the chromate (CrO_4_^2–^), chromate–permanganate, and other mixed crystals of the [(Fe, Cr)(urea)_6_][(CrO_4_, SO_4_, Cr_2_O_7_, S_2_O_7_, S_2_O_8_)*_n_*(MnO_4_, ClO_4_)*_m_*] systems.

## Results and Discussion

### Synthesis and Properties of Compound **1**

[Hexakis(urea-*O*)iron(III)] dichromate (compound **1**) was isolated first by Barbieri^34^ in the reaction of aqueous [hexakis(urea-*O*)iron(III)] nitrate and sodium dichromate solutions by the slow concentration and crystallization of the mother liquor.^34^ However, apart from its composition and color, the properties of compound **1** are not known. The dichromate ion is stable only in acidic solutions.^45^ Our HPLC-MS-CI studies on a hexaureairon(III) nitrate precursor solution showed the presence of mixed aquo-urea-iron(III) species (Figure S1, where *m*/*z* values were found for aquo and mixed urea-aquo species without *m*/*z* values of the exclusively urea-coordinated ones), the hydrolysis reactions of which result, at sufficiently low pH values, in the isolation of the dichromate complex without the formation of chromate ions. Following Barbieri’s method,^34^ we isolated compound **1** in 59% yield as orange-colored crystals. Compound **1** dissolves in distilled water (*s* = 37.0 g/L at 20 °C), and the saturated solution is orange-yellowish and, as expected, strongly acidic (pH ∼1.08). It does not dissolve in benzene, diethyl ether, or carbon tetrachloride; it neither reacts with nor dissolves in anhydrous ethanol at room temperature. Compound **1** is stable at room temperature in the dark for more than a month. The pycnometric density (*d*) is determined at 25 °C in chloroform as to be 1.809 g/mL.

Yellow hexagonal prisms (Figure S2) of compound **1** were grown from its saturated aqueous solution at room temperature. Differential scanning calorimetry (DSC) studies showed no phase transition between −173 and 288 K (Figure S3). Because the crystallographic features, including lattice packing and the presence, number, and nature of hydrogen bonds between the anions and cations, are essential points strongly related to the expected quasi-intramolecular redox reactions of compound **1**, the results of single-crystal X-ray structure determination are discussed below in detail.

### Crystal Structure of Compound **1**

[Hexakis(urea)iron(III)] dichromate (2:3) crystallizes in a trigonal system [*a* = 18.6756(13) Å, *c* = 27.2545(9) Å at 128(2) K, *Z* = 6] in space group *R*3̅*c*. Cell parameters and selected crystal structure data of compound **1** are listed in Tables S1–S7. The powder X-ray diffraction pattern of compound **1** is shown in Figure S4, agreeing well with the calculated pattern [derived from synchrotron X-ray diffraction(SXRD)]. Compound **1** contains a hexacoordinated Fe^III^ cation surrounded by six urea ligands arranged in an octahedral geometry. The asymmetric unit of compound **1** contains one-third of an iron(III) ion, two urea molecules, and one-half of a dichromate anion (Figure S5).

**Figure 2:**
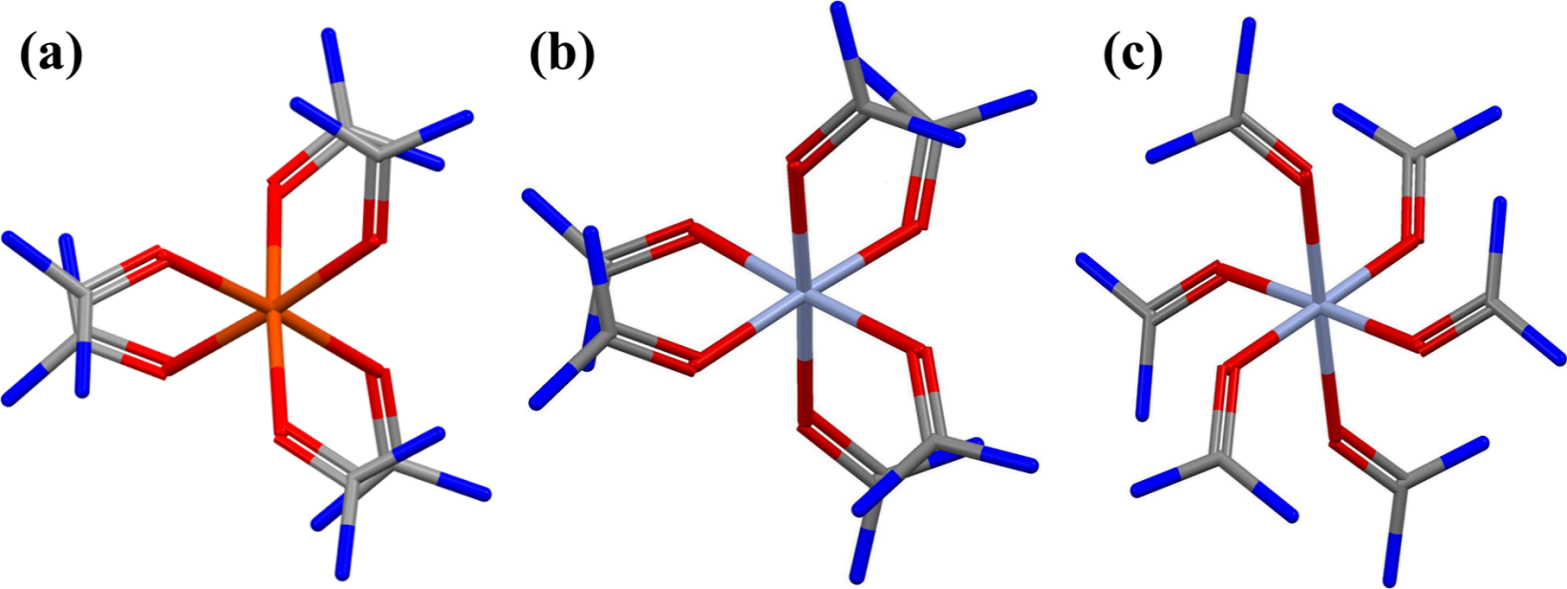
Comparison of the (a) single-cation conformation in compound **1** and (b and c) the two types of cation conformations in compound **1a**. The urea molecules in the foreground are marked with dark colors, and those in the background are marked with light colors.

The urea molecules are arranged like blades of a ship propeller at the two sides of the iron(III) ion (Λ/Δ). The orientation of the urea molecules is always uniform on one side of the complex, which gives the complex a sort of chiral feature. The twists of the urea molecules at the two sides of the complex ion are opposite, and the complex ion itself is not centrosymmetric. However, the complex compound crystallizes in a centrosymmetric space group, and the structure contains both enantiomers of the complex ion. The asymmetric unit of the iron complex contains one-third of the complex cations, whereas analogous Cr compound **1a** (CCSD code PEDWIG) contains two-thirds of the complex cations. The conformations of the two entire Cr complex cations are different (Figure 2b,c), but one of them is similar to that of the iron complex; the rotation directions of the urea propellers are different at the two sides of the cations for (a) compound **1** and (b) compound **1a**. In the case of compound **1a**, the rotation directions of the urea propellers at the two sides of the complex ion are the same (c).

**Figure 3:**
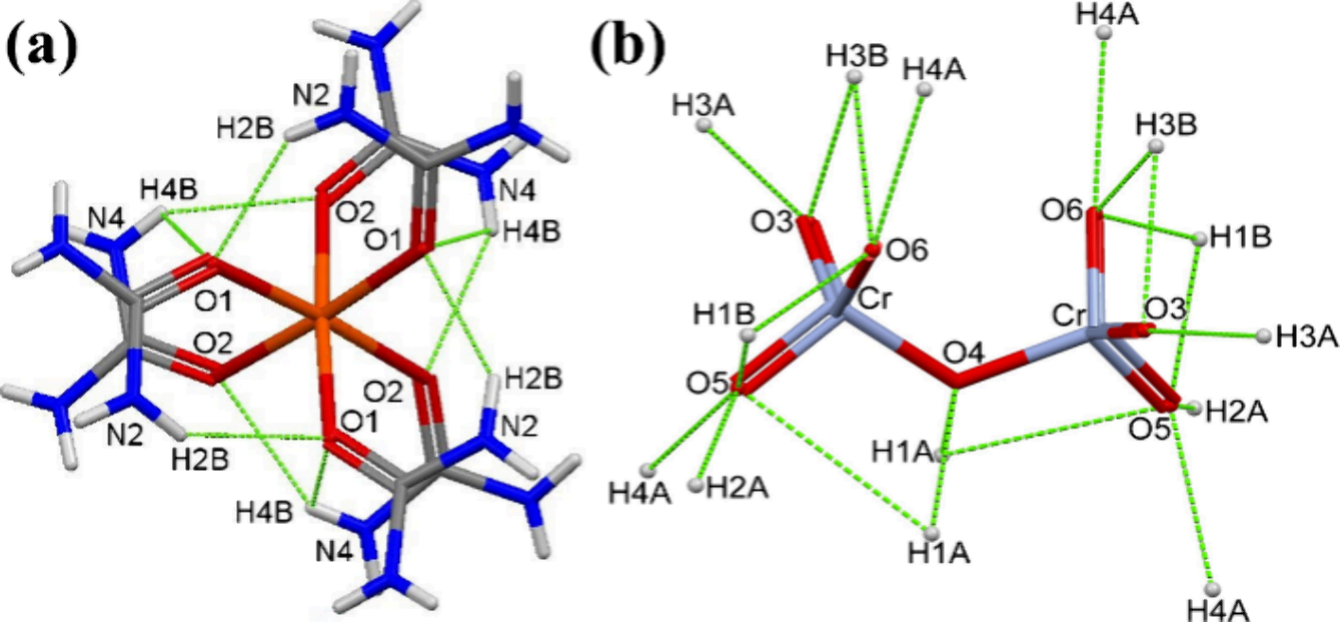
(a) Intramolecular and (b) intermolecular hydrogen bonds in the structure of compound **1**.

The “a” and “b” types of conformations are chiral; however, *R*3̅*c* is a centrosymmetric space group, and the structure contains both enantiomers. In the case of the “c” type, the molecule has an inversion center at the chromium atom and is therefore achiral. The conformation of the complex is fixed by nine internal hydrogen bonds (Figure 3a). All six urea ligands are involved in intramolecular hydrogen bonds, and every urea makes this kind of bonding with only one of its amino groups; accordingly, three urea ligands form monodentate hydrogen bonds, and the other three bifurcated intramolecular hydrogen bonds. Namely, two kinds of urea exist in the structure of compound **1** (urea ligands marked with N1 and N2 as well as with N3 and N4 amino groups). Accordingly, there are two kinds of oxygen atoms of urea, O1 and O2. Every O2-type oxygen atom makes one hydrogen bond with H4B hydrogens, and the H4B hydrogens are bifurcated; each of the three is coordinated to its closest O1 and O2 simultaneously. The O1 atoms are bound in a bidentate fashion with H4B and H2B. The bifurcated hydrogen bonds are stronger than the monocoordinated ones, and both are shorter (stronger) than the hydrogen bonds that exist between the urea hydrogens and dichromate oxygens. The intra- and intermolecular hydrogen bonds in the structure of compound **1** are listed in Table 1 and presented in Figure 3.

**Table 1:** Hydrogen Bonds in the Solid State Structure of Bis[hexakis(urea)-iron(III)] Tris(dichromate)

D–H···A	D–H (Å)	H···A (Å)	D···A (Å)	D–H···A (deg)	symmetry operator
N1–H1A···O4	0.84(4)	2.16(3)	2.980(4)	166(3)	1555
N1–H1B···O6	0.84(3)	2.28(3)	3.047(4)	153(3)	8555
N2–H2A···O5	0.84(3)	2.11(3)	2.912(5)	161(3)	8555
N2–H2B···O1 intra	0.839(18)	2.12(2)	2.854(4)	146(3)	3555
N3–H3A···O3	0.84(2)	2.04(3)	2.866(4)	168(3)	18 555
N3–H3B···O3	0.83(3)	2.31(3)	3.029(6)	145(3)	24 445
N3–H3B···O6	0.83(3)	2.44(3)	3.158(5)	358(4)	24 445
N4–H4A···O5	0.84(3)	2.57(3)	3.086(4)	121(3)	16 445
N4–H4A···O6	0.84(3)	2.40(3)	3.150(6)	354(4)	24 445
N4–H4B···O2 intra	0.82(4)	2.23(4)	2.944(5)	146(3)	2555

Every dichromate ion makes intermolecular hydrogen bonds with six kinds of urea hydrogens (Table 1), and all oxygen atoms of each dichromate ion are involved in these interactions. The urea ligands, which form monocoordinated intramolecular interactions (H2B), are also attached to the three terminal oxygens of 1.5 dichromate ions (two urea ligands/dichromate anion having two terminal CrO_3_ units), through the second hydrogen (H2A) of the same N atom (N2). Thus, N2 amino groups are involved in both intra- and intermolecular interactions. The second amino group of these urea ligands (N1) makes three bifurcated hydrogen bonds (two per dichromate) with the bridging oxygen of the dichromate ions (O4) via the H1A hydrogens, which are bifurcatedly coordinated to terminal dichromate oxygens (O5), as well. Each N1 and N2 urea ligand (three) is bound to one O5 atom of different CrO_3_ units [CrO_3_:Fe(urea)_6_ ratio of 1.5, three CrO_3_ units] of the dichromate ions. The second hydrogens of N1 atoms (H1B) are bridged by two terminal oxygens (O5 and O6) of the two halves of terminal CrO_3_ units in each dichromate ion. The two sets of terminal O3 and O6 atoms of the dichromate anions are bound to monocoordinated H3A and H4A, respectively, and the H3B atoms link these two terminal CrO_3_ atoms. Thus, the O3, O4, and O6 atoms of the terminal CrO_3_ units of each dichromate ion are involved in bidentate (H3A and H3B), tridentate (H1B, H3B, and H4A), and tetradentate (H1A, H1B, H2A, and H4A) hydrogen bond interactions, respectively. Both hydrogens of the N1 and N3 atoms are simultaneously coordinated; on the contrary, one hydrogen of the N2 and N4 atoms each is bound in intramolecular interactions, and the other hydrogens are bound in intermolecular interactions.

**Figure 4:**
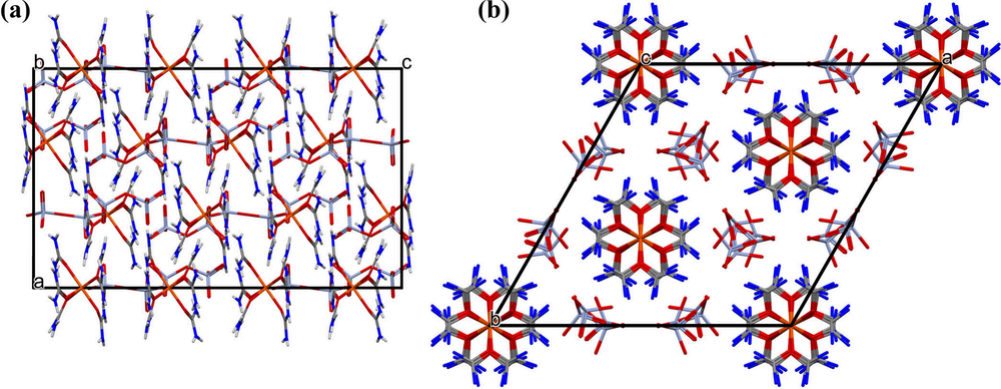
Packing in the unit cell of compound **1**, viewed along the (a) *a*- and (b) *c*-axes.

The shortest distance between the iron ions in the structure is 7.149 Å (Figure S6), longer than in analogue [(hexakis(urea)iron(III)] permanganate (7.037 and 6.667 Å for the two kinds of the complex cation)^46^ and persulfate (6.433 and 6.532 Å for the two kinds of the complex cation).^47^ The packing arrangement of the molecules is presented in Figure 4. The urea propellers are always arranged in the direction of the *c*-axis.

**Figure 5:**
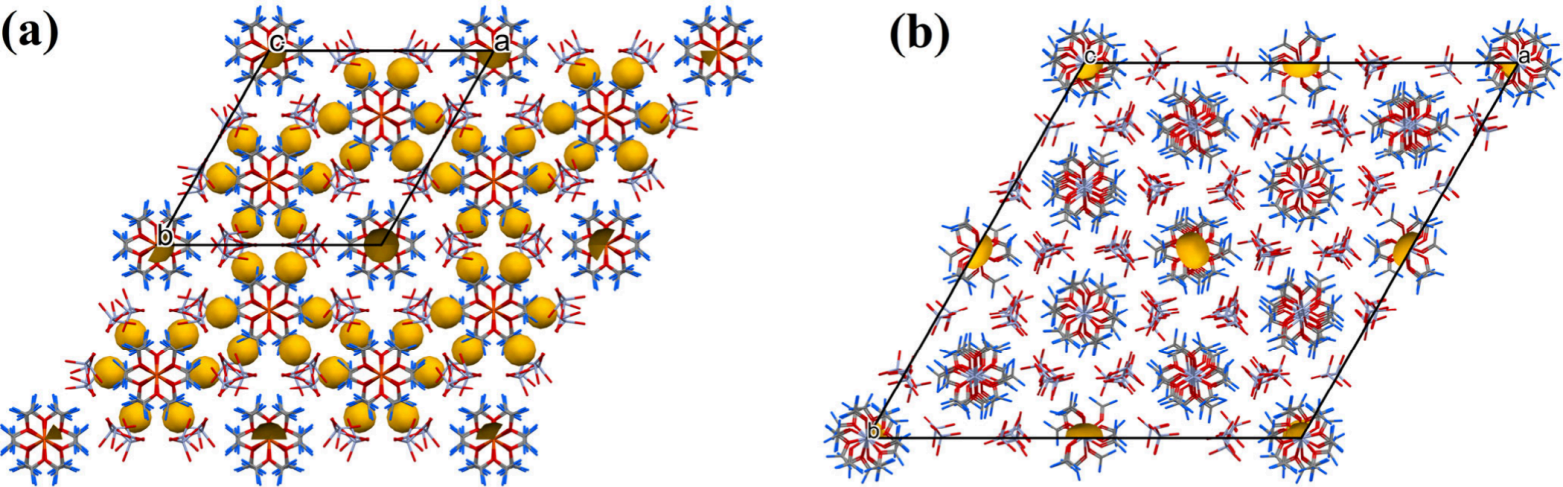
Potential solvent accessible voids in the unit cells of (a) compound **1** and (b) compound **1a**.^48^

The structures of compounds **1** and **1a** contain 1.8% and 0.4% of potential solvent accessible voids with volumes of 147.7 and 120.2 Å^3^ per unit cell, respectively (Figure 5), and with the Kitaigorodskii packing coefficients 66.9% and 69.2%, respectively^48^ (Figure 5).

The packing of the isomorphous [hexakis(urea)chromium(III)] dichromate (PEDWIG in CCSD) is given in Figure S7.^48^ Both complexes crystallize in the trigonal system (*R*3̅*c*). The lengths of the *a* and *b* unit cell axes of the chromium complex are twice those of the iron complex, and thus, the cell volume of the chromium complex is 4 times greater than that of the iron complex (the *c* unit cell axis lengths are similar).

### Results of Vibrational Spectroscopy of Compound **1**

Compound **1** contains two different urea ligands and one kind of dichromate ion in its asymmetric unit (*Z* = 6). The assignment of the urea ligand modes coordinated to iron(III) has already been studied in detail,^46,47^ and the vibrational analysis of the dichromate ion and the assignments of its 21 normal modes were also reported.^49^ On the basis of these results and the correlation analysis of the [Fe(urea)_6_]^3+^ cation and dichromate anion in compound **1**, the peaks in the infrared (IR), Raman, low-temperature Raman, and far-IR spectra of compound **1** were assigned. To unambiguously assign (as much as possible) the coinciding urea ligand peaks, the IR spectrum of deuterated compound **1** (compound **1-D**) was also recorded. The vibrational modes of urea ligands in compounds **1** and **1-D** are listed in Table S8, the IR and Raman bands of dichromate anions in compound **1** are listed in Table 2.

**Table 2:** Assignment of the IR and Raman Bands of the Dichromate Anions of Compound **1**

IR (cm^–1^)	Raman (cm^–1^)	assignment^49^
931	937	ν_as_(CrO_3_)
905	908	ν_s_(CrO_3_) in-phase
887	892, 848	ν_s_(CrO_3_) out-of-phase
767, 753	–	ν_as_(Cr–O–Cr)
499, 475	–	ν_s_(Cr–O–Cr)
385	396, 376	δ_s_(CrO_3_)
365	365, 342	δ_as_(CrO_3_)
330, 264	316, 246	ρ(CrO_3_)
220	217, 213, 194	δ(Cr–O–Cr)
167, 133	140, 123	ρ(CrO_3_)

The primitive cell contains 24 urea molecules, six dichromate(VI) anions, and four iron(III) cations. The results of the correlation analysis for the urea ligand modes are given and are summarized in Text S1 and Figure S8.

**Figure 6:**
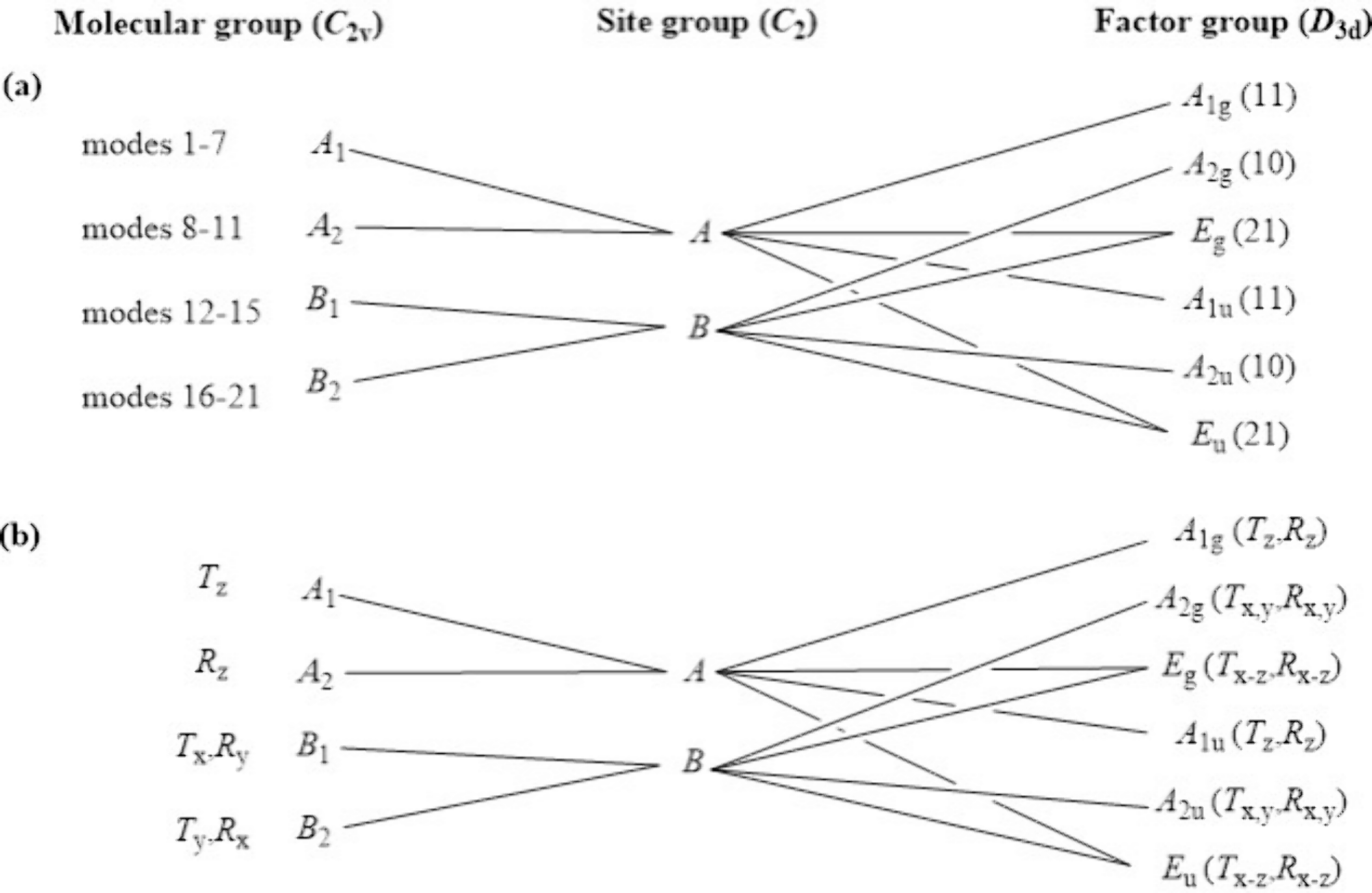
(a) Internal and (b) external vibrations of dichromate anions in compound **1**.

The isolated dichromate ion has a total of 21 internal normal modes under *C*_2*v*_ symmetry (7*A*_1_, 4*A*_2_, 4*B*_1_, and 6*B*). The correlation analysis results for the dichromate anion in compound **1** can be seen in Figure 6a (internal vibrations) and Figure 6b (external vibrations).

The O_3_CrOCrO_3_^2–^ anion belongs to the *C*_2*v*_ point group and shows 17 IR active internal vibrational modes,^49^ whereas in compound **1**, all of the modes are IR and Raman active (Figure 6). The 21 normal vibrational modes of the Cr_2_O_7_^2–^ ion can be classified into in-phase and out-of-phase coupling motions of the terminal CrO_3_ groups and the skeletal vibrations of the CrOCr bridge (Table 2). The external vibrations of the dichromate anions in compound **1** include a total of 18 hindered translations and 18 hindered rotations (equal to 36 external vibrational degrees of freedom).

The central Fe^3+^ cations have hindered translations (a total of 12 vibrational degrees of freedom) (Figure S9).

In total, there are 102 translational degrees of freedom, three of which are acoustic modes (*A*_2*u*_ + *E_u_*), and the remaining 99 belong to hindered translations. There are also 90 rotational degrees of freedom and altogether 558 vibrational degrees of freedom belonging to internal vibrations, thus giving altogether 750 degrees of freedom, which equals 250 (number of atoms in the primitive cell) × 3.

The vibrational modes of the dichromate ion (*C*_2*v*_) may be classified into two main categories, namely, the terminal (O–CrO_3_) and bridging (Cr–O–Cr) modes:^50,51^
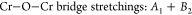

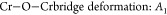

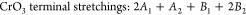

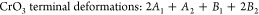

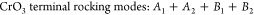

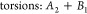


The observed vibrational modes for the dichromate ion in the IR spectrum of compound **1** (Figures S10 and S11) were assigned on the basis of correlation analysis and normal coordinate analysis of the dichromate ion.^49−53^ The terminal antisymmetric, terminal symmetric in-phase, and terminal symmetric out-of-phase stretches were found at 931, 905, and 887 cm^–1^, respectively. The symmetric and antisymmetric stretching modes of the Cr–O–Cr bridge occurred at 499/475 and 767/753 cm^–1^, respectively. The other normal modes of the dichromate ions are located in the far-IR region. A wide weak band between 385 and 365 cm^–1^ contains the terminal symmetric bending (scissoring deformation) modes. A combination band at 330 cm^–1^, a terminal rocking mode at 264 cm^–1^, and a bridging bending mode at 220 cm^–1^ also appeared. The CrO_3_ torsion modes were found at 167 and 133 cm^–1^.

**Figure 7:**
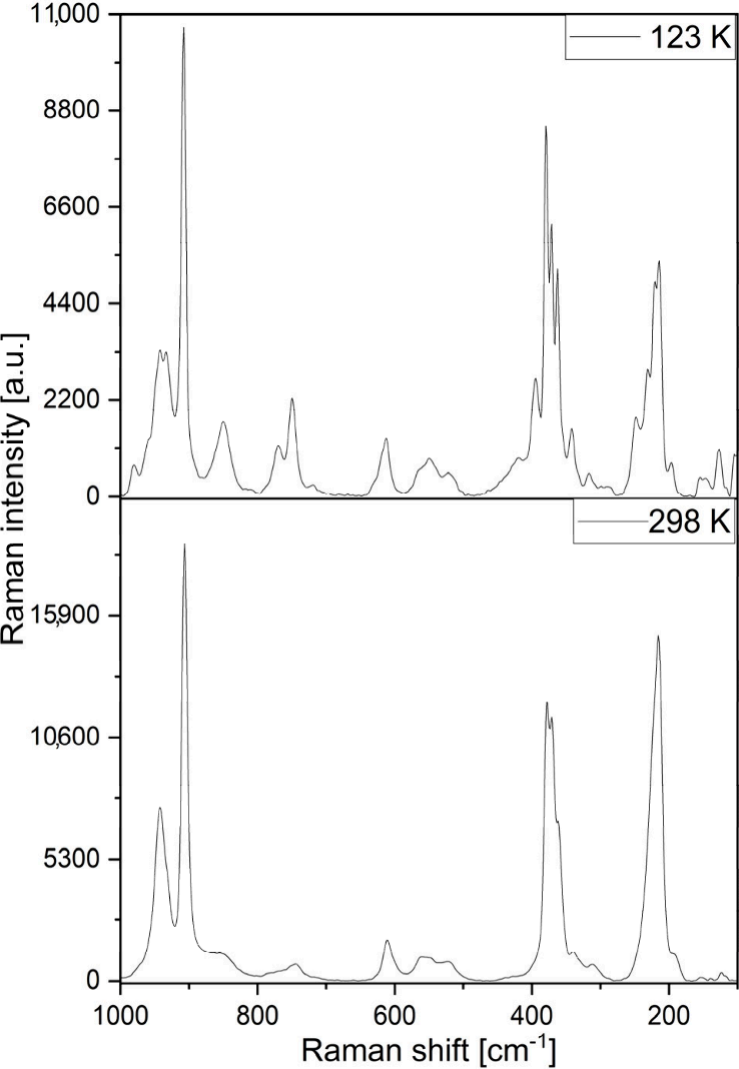
Raman spectra of compound **1** at 123 and 298 K (scanned using the 532 nm excitation line of a laser).

The Raman spectra of compound **1**, recorded at two irradiation wavelengths (532 and 785 nm) and two temperatures (123 and 298 K), were found to be more informative for the dichromate anion than for urea modes (Figure 7 and Figures S12 and S13). The urea and dichromate modes were evaluated from the spectra recorded with 532 and 785 nm excitations, respectively, but most of the urea modes were too weak to be detected. The dichromate terminal stretching modes appeared at 937, 908, 892, and 848 cm^–1^, and the most intense band was the symmetric in-plane stretching mode at 908 cm^–1^. The bands at 396/376 and 365/342 cm^–1^ belong to the symmetric and antisymmetric stretching of the terminal and bridge groups, respectively.^54,55^ The band at 316 cm^–1^ was assigned as the combination band, analogous to that found at 330 cm^–1^ in the IR spectrum. The terminal rocking mode appeared at 246 cm^–1^, whereas the bridging deformation mode bands occurred at 217, 213, and 194 cm^–1^. The torsion modes were found at 140 and 123 cm^–1^.

Due to the coincidence of some dichromate and urea ligand bands in the ranges of 510 and 760 cm^–1^, the IR spectrum of deuterated compound **1** was also recorded. No bands of N–H(D) group modes were found in this spectral range; thus, these bands may belong to C–O/C–N groups and dichromate anions. However, the shape and position variations of the deuterated counterpart suggest that bands in the range of 510 and 760 cm^–1^ more likely belong to the urea ligand than to the modes of the dichromate anions^47,56^ (Figure S13).

### Solid-Phase Ultraviolet–Visible (UV–vis) Spectroscopy Study of Compound **1**

**Figure 8:**
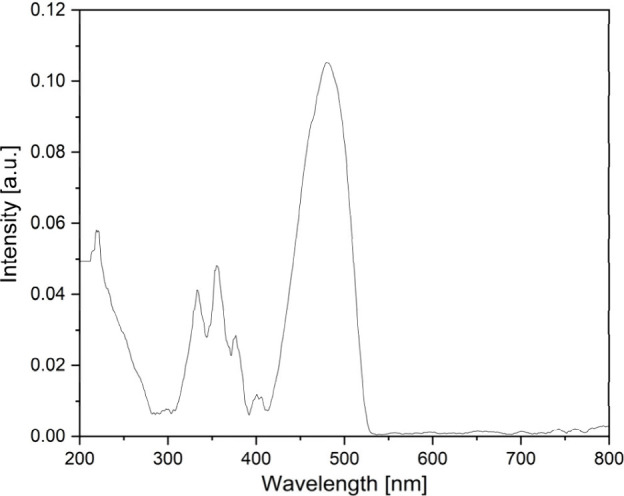
Raman spectra of compound **1** at 123 and 298 K (scanned using the 532 nm excitation line of a laser).

Due to the possible electron transitions of the [Fe(urea)_6_]^3+^ cation^46,47,57,58^ and dichromate anion,^59−64^ a large number of bands are present in the UV–vis spectra of compound **1** (Figure 8). Furthermore, there are some overlaps between the electron transition bands of the anion and the cation. In the UV–vis spectra, three main peaks belonging to the dichromate anion are expected.^59−64^ From the ground state, that is ^1^*A*_1_ because the *t*_1_ orbital is fully filled, electrons can be excited to the empty antibonding *t*_2_ and *e_g_* orbitals.^60^ In compound **1**, the corresponding bands can be found around 250 and 370 nm [^1^*T*_2_(*t*^5^_1_*t*^1^_2_) ← ^1^*A*_1_ and ^1^*T*_2_(*t*^5^_1_*e*^1^) ← ^1^*A*_1_, respectively]. However, the first band overlaps with the n−π* and π–π* transitions of the urea ligand (Fe^III^–O=C LMCT bands), which also occurs for the hexakis(urea)-iron(III) complexes forming a wide band at ∼230 nm.^46,47,57^ Furthermore, this band has at least three shoulders that also suggest that the different CrO_4_ parts of the anion have different hydrogen bonding systems (see above), resulting in a slight change in the symmetry.^59−64^ In contrast, the *T*_2_(*t*^5^_1_*e*^1^) ← ^1^*A*_1_ transition can be ambiguously assigned, because this transition appears with a fine structure^60−63^ at 333, 355, and 377 nm due to the vibration–electronic coupling.^60^ The splitting and the shift of the peaks, as compared to the literature data, also originated from the breaking of the symmetry from *T_d_* (free Cr_2_O_7_^2–^ anion) to *D*_3*d*_ due to the different Cr–O bond distances (confirmed by SXRD). At higher wavelengths, the ^4^*A*_*g*_, ^4^*E_g_*(*G*) ← ^6^*A*_1_ transitions^65−67^ of the cation can be assigned as a high-intensity and broadened peak at 480 nm with the combination of the LMCT transition of the dichromate anion.^57,59^ In the form of low-intensity and broad peaks, the ^4^*T*_2*g*_(*G*) ← ^6^*A*_1_ and ^4^*T*_1*g*_(*G*) ← ^6^*A*_1_ transitions can be found at ∼590 and ∼800 nm, respectively, belonging to the complex cation.

### Selective Oxidations of Organic Substances with Compound **1**

Some transition metal permanganate complexes containing pyridine, bipyridine, or ammonia ligands were found to be mild and selective oxidants in various organic reactions,^68,69^ and Haghgooie et al.^70^ and Zhang et al.^71^ found that some [hexakis(urea)iron(III)] salts such as nitrate, chloride, and sulfate catalyzed organic oxidation reactions. Recently, we found^46^ that [hexakis(urea)iron(III)] permanganate performed well as an oxidizing agent in the reactions of (un)substituted benzyl alcohols with the formation of benzaldehydes and benzonitriles. Therefore, we expanded our studies to the oxidation of some organic compounds with compound **1**, including the (un)substituted benzyl alcohols and 2-octanol used previously.^46^ Compound **1**, however, was found to be a less effective oxidizing agent; the level of conversion of alcohols did not exceed 22% (even at the reflux temperature of benzene), and the yield of benzonitriles was ∼10% (Table S9). The reactions of compound **1** with some more oxidation-sensitive sulfur compounds such as diphenyl sulfide and 2-mercaptoethanol as well as some amine compounds were also tested in benzene at room temperature and at reflux temperature (of benzene) with durations of 2 and 4 h. The results of these oxidation reactions are summarized in Table 3, and more detailed data are listed in Table S9.

**Table 3:** Oxidation Test Results of Sulfur-Containing Organic Compounds with Compound **1**

		conversion (%)		
substrate	product	*t* = 2 h, *T* = 25 °C	*t* = 2 h, *T* = 80 °C	*t* = 4 h, *T* = 80 °C
diphenyl sulfide	diphenyl sulfide	98.03	97.67	–
	diphenyl sulfoxide	0.48	0.89	66.45
	diphenyl sulfone	–	–	0.69
	diphenyl disulfone	0.51	0.42	14.31
	biphenyl	0.49	0.52	14.93
	thianthrene	0.49	0.50	3.62
2-mercaptoethanol	2-hydroxyethyl disulfide	100	100	100

The level of conversion of diphenyl sulfide reached 100% (*T* = 80 °C, *t* = 4 h), but the product distribution depended on the temperature and reaction time (Table 3). Sulfur elimination was observed with biphenyl formation for 4 h at 80 °C. The low reactivity of compound **1** with respect to alcoholic functional groups resulted in the formation of 100% bis(2-hydroxyethyl) disulfide even at room temperature. The dimerization of the thioalcoholic group into disulfide shows that compound **1** is a very mild and selective oxidant (Table 3).

The oxidation of hexylamine was studied at reflux temperature, and ∼80% conversion could be reached. The reaction may involve oxidation of the last carbon atom, which was confirmed by the appearance of the hexanoyl group [C_5_H_11_C(=O)-] (*m*/*z* 112). A series of reaction products were detected, *n*-hexyl hexanoylamide, di-*n*-hexylamine, and *n*-hexanonitrile, but the main product was *n*-hexylpiperidine. This means that some oxidative condensation reactions appear (up to *m*/*z* 250 and fragments with even higher *m*/*z* values), including consecutive elimination and/or ring-closure steps as was found earlier in the oxidation of amines with metal permanganates.^72−75^

### Thermal Decomposition Features of Compound **1**

The thermal decomposition of compound **1** was monitored with TG-DTG, TG-MS, and DSC methods in an inert and air atmosphere (Figures S14–S18). The thermal decomposition began with an endothermic ligand loss process just like for the thermal decomposition of [hexakis(urea-*O*)iron(III)] nitrate.^58^ The ligand loss started around 110 °C (Table S10), but the oxidation of the liberated urea started immediately and appeared as a subsequent, but coinciding, exothermic process. The liberated (non-oxidized) urea ligands melt and then decompose in the molten state as usual. The thermal decomposition features and the reaction heats were very similar in the first three decomposition steps (Table S10), in both an inert and an air atmosphere; thus, the oxygen content of the air had no serious influence on these decomposition processes below 250 °C. The next decomposition step involved at least three different processes, corresponding to the 275, 290, and 312 °C DTG peak temperatures in the presence of oxygen, and the reaction heats differed by 1 order of magnitude in the inert and air atmospheres (103.7 and 766.5 kJ/mol, respectively). Thus, the fourth step in the oxidative atmosphere included combustion processes, as well. Altogether, an intense combustion process was confirmed by the DSC results, performed in argon-containing (ΣΔ*H* = 516.3 kJ/mol) and oxygen-containing (ΣΔ*H* = 1178.1 kJ/mol) atmospheres (Table S10).

The thermal decomposition of urea with H_2_O or NH_3_ formation above 135 °C^76^ and the solid-phase quasi-intramolecular redox reactions in compound **1** proceed in parallel; therefore, the formation of H_2_O and NH_3_ detected by TG-MS in itself does not confirm the presence of the redox reaction. However, the formation of CO/N_2_ and NO at ∼160 °C in an exothermic reaction confirms the ongoing solid-phase quasi-intramolecular redox reactions

### Heat Treatment of Compound **1**

**Figure 9:**
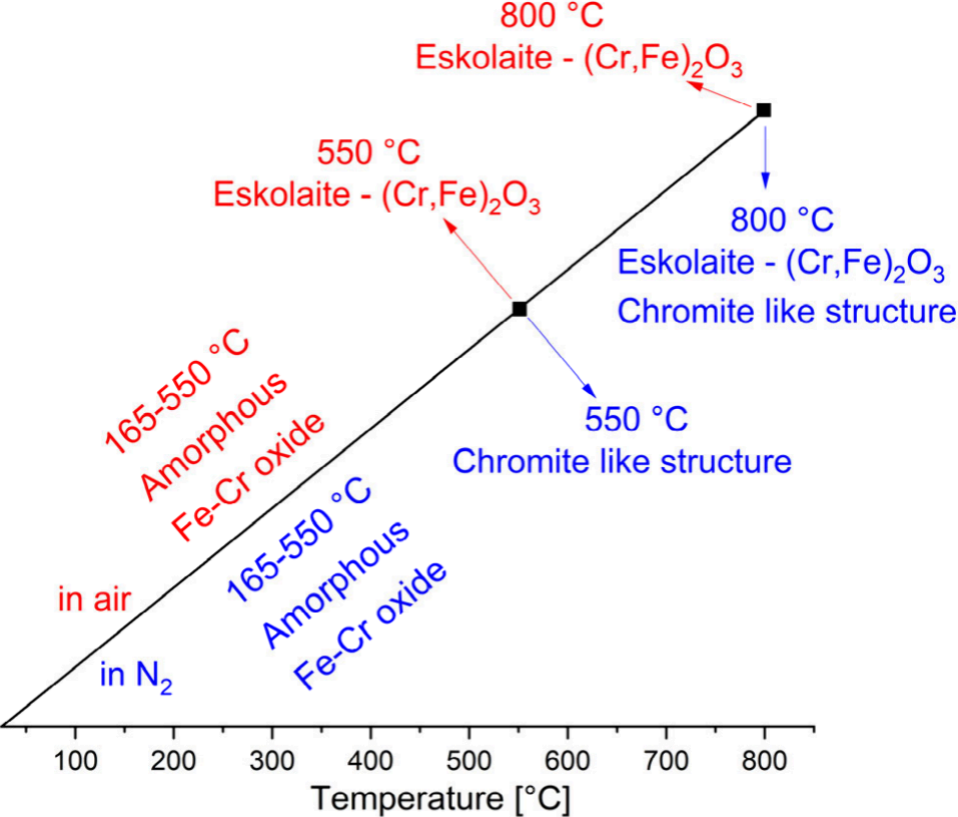
Summary of the intermediate and final products of the oxidative and inert atmosphere heat treatments (the calcination time was 2 h in all cases) of compound **1**.

**Figure 10:**
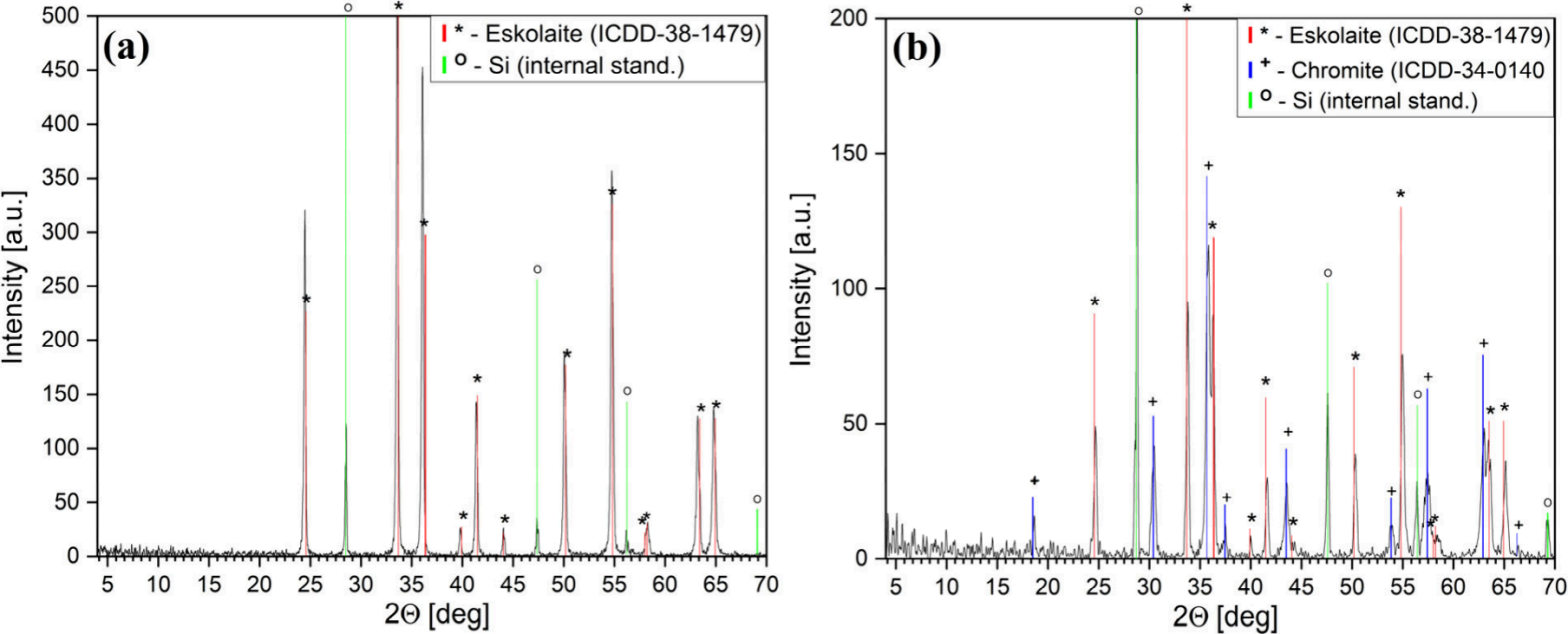
PXRD of the final products of the (a) oxidative and (b) inert atmosphere heat treatments of compound **1**.

To understand the features of the decomposition processes during the heat treatment of compound **1**, the chemical and physical parameters of the decomposition intermediates were studied. The intermediates were prepared in a laboratory furnace in both oxidative and inert (N_2_) atmospheres by isotherm heating at 165, 200, 320, 550, and 800 °C; these temperatures were selected according to the DTG decomposition temperatures of compound **1** (Figures S14 and S15). The calcination time was 2 h in all cases. The chemical nature of the intermediate and final products of the heat treatments in both atmospheres is summarized in Figure 9. The final products were as follows: a single-phase iron(III)–chromium(III) mixed oxide with an eskolaite-like (ICDD-PDF 38-1479^77^) structure in air (Figure 10a) and a two-phase system containing the same eskolaite-like Fe–Cr mixed oxide plus a chromite (spinel^78^) [ICDD-PDF 34-0140^79^ (Figure 10b)] in an inert atmosphere. The weight losses were 55.17% and 58.45% in the oxidative and inert atmospheres, respectively.

**Figure 11:**
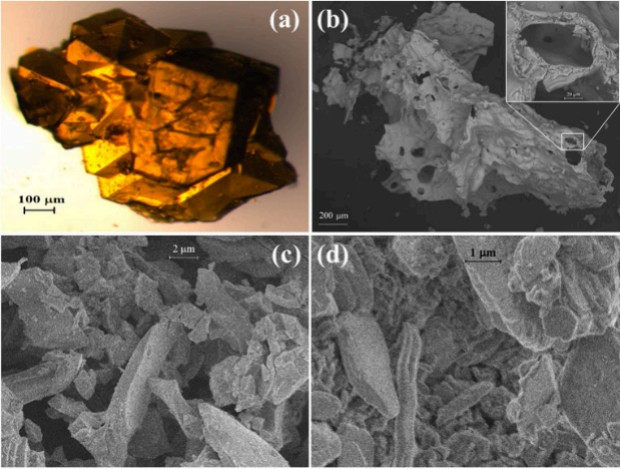
(a) Microscope picture of a crystallite of compound **1** before annealing and SEM images of the decomposition intermediate prepared at (b) 350 °C in air and (c) 550 °C in a N_2_ atmosphere and (d) the final product obtained at 800 °C in an oxidative atmosphere.

According to PXRD and SAED measurements (Figures S19 and S20, respectively), amorphous iron–chromium mixed oxides with a homogeneous iron and chromium distribution (Figure S20) formed in both oxidative and inert atmospheres between 165 and 550 °C. The diffraction rings of the SAED measurements correspond to the (104), (202), and (214) lattice planes (*d* = 2.60, 2.04, and 1.48 Å, respectively) of an eskolaite-like structure (Figure S20). In Figure 11, the microscope and scanning electron microscopy (SEM) pictures show the morphologic transformation of compound **1** (Figure 11). The SEM images show the formation of a solidified molten layer at the edges of the particles of compound **1** subjected to heat treatment at ∼350 °C in an oxidative atmosphere (Figure 11b). The observation of such a molten layer agrees well with the DSC results (Figures S17 and S18), which suggest endothermic ligand loss and melting processes. Figure 11b demonstrates a solidified material with bubble-shaped holes, which may be attributed to the formation of gases in a viscous liquid (NH_3_, H_2_O, and other gases in molten urea). The observation of these holes also suggests the exothermic reaction in the molten urea. At 550 °C (in a N_2_ atmosphere) and at 800 °C (in air), micro- and nanosize particles formed (Figure 11c,d).

**Figure 12:**
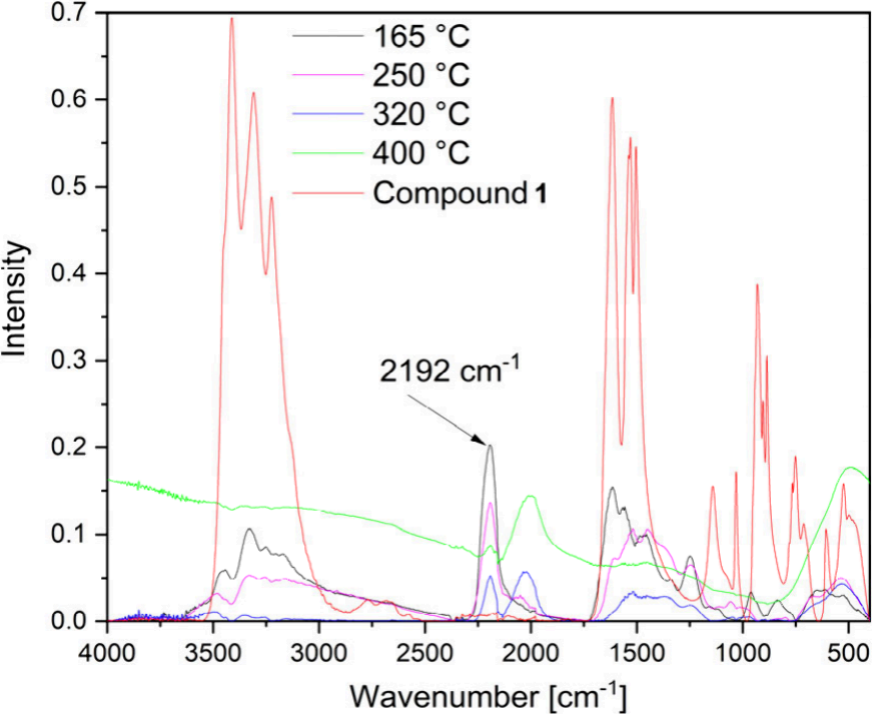
IR spectra of the decomposition intermediate phases formed during heat treatments of compound **1** in an inert atmosphere.

At temperatures of >550 °C, Mössbauer spectroscopy confirmed that the samples prepared in the oxidative atmosphere had only one iron(III) chemical environment (≤800 °C). On the contrary, in the case of the N_2_ atmosphere, the appearance of an iron(II)-like chemical environment (∼6%) was also detected around 325 °C (Tables S11–S13 and Figure S21).^80^ The presence of some organic residue of the decomposition of compound **1** was detected by IR spectroscopy at 165 °C (Figure 12), and the reduction of Fe^III^ to Fe^II^ started above this temperature. Upon further heat treatment, the amount of the Fe^2+^-containing phase increased (Figure S22 and Table S13). However, at 350 °C, there was no organic residue left (Figure 12).

Interestingly, two IR peaks appear at 2198 and 2031 cm^–1^ in the sample prepared at 320 °C in an inert atmosphere (Figure 12). These peaks can be attributed to the ν_C≡N_ modes^81,82^ of the Fe–C≡N–Cr(Fe) linkage-containing phases.^80^ The intense peak at 530 cm^–1^ belongs to the ν_M–C_ mode (M = Fe or Cr)^82^ (Figure 12). The Mössbauer spectroscopy results support these IR assignations, because a spectral component with an isomer shift (*δ*) of 0.217 mm/s and a quadrupole splitting (*Δ*) of 1.106 mm/s was found, indicating the formation of a low-spin iron(III) species (note that the CN moiety is a strong ligand) and another component with a *δ* of 0.347 mm/s and a *Δ* of 1.787 mm/s that shows the formation of an iron(III) chemical environment with strong tetragonal distortion (note the quasi-linear Fe coordination environment in the chain structure) (Figure S22 and Table S13). Upon further heat treatment, the amount of this low-spin Fe^III^ phase starts to decrease, and it disappears at 550 °C (Table S13). Because the formation of NO (*m*/*z* 30) and CO_2_/N_2_O (*m*/*z* 44) is detected (and there are no *m*/*z* 42 and 43 peaks as determined by TG-MS) in an inert atmosphere for heat treatments between 350 and 400 °C (Figure S16), the oxidation of the CN^–^ bridges should take place by the oxide phases present as supported by Mössbauer spectroscopy. Because the amount of Fe^III^ species decreases and the amount of Fe^II^ species increases, the Mössbauer data suggest that the oxidizing agent in this reaction is the Fe^III^ ion (Table S13).

**Figure 13:**
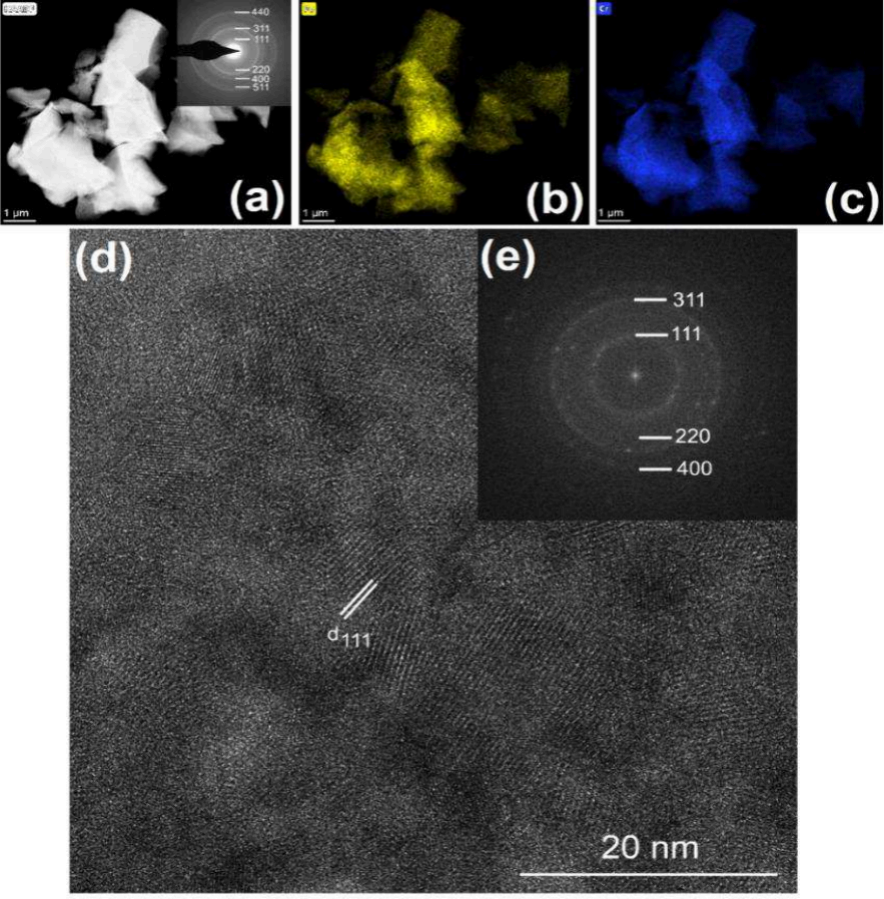
(a) HAADF-STEM image, (b) Fe and (c) Cr elemental maps, and (d) HRTEM image and (e) its corresponding FFT of a sample prepared at 600 °C in an inert atmosphere. White lines in panel d mark the d_111_ lattice sheet separation of the chromite phase.

On the basis of the PXRD^79^ (Figure S19b) and Mössbauer measurements^83^ (Table 4) above 550 °C (≤700 °C) in an inert atmosphere, the product is unambiguously chromite. The HAADF-STEM image of this sample shows several micrometer-sized particles consisting of 5–10 nm chromite nanograins with a homogeneous Fe–Cr distribution (Figure 13).

**Table 4:** Mössbauer Parameters, at Different Temperatures, of the Intermediate Phases Formed in an Inert Atmosphere at 600 °C^a^

	90 K				120 K			
	Fe^II^		(Fe^II^ + Fe^III^)/2		Fe^II^		(Fe^II^ + Fe^III^)/2	
relative area (%)	34	41	19	6	39	23	24	14
*δ* (mm s^–1^)	1.057 ± 0.011	1.051 ± 0.003	0.665 ± 0.025	0.766 ± 0.021	1.093 ± 0.009	1.038 ± 0.004	0.726 ± 0.017	0.778 ± 0.019
*Δ* (mm s^–1^)	2.075 ± 0.040	2.858 ± 0.015	0.764 ± 0.055	1.776 ± 0.048	2.106 ± 0.032	2.834 ± 0.017	0.702 ± 0.046	1.918 ± 0.035
*Γ* (mm s^–1^)	0.611 ± 0.074	0.474 ± 0.022	0.734 ± 0.080	0.395 ± 0.103	0.574 ± 0.043	0.369 ± 0.027	0.833 ± 0.090	0.463 ± 0.071

	160 K				200 K			
	Fe^II^		(Fe^II^ + Fe^III^)/2		Fe^II^		(Fe^II^ + Fe^III^)/2	
relative area (%)	43	24	19	14	38	24	18	20
*δ*(mm s^–1^)	1.046 ± 0.010	1.014 ± 0.005	0.734 ± 0.014	0.702 ± 0.013	1.036 ± 0.012	0.981 ± 0.005	0.724 ± 0.012	0.673 ± 0.013
*Δ* (mm s^–1^)	1.891 ± 0.030	2.715 ± 0.018	0.463 ± 0.042	1.633 ± 0.031	1.760 ± 0.040	2.607 ± 0.027	0.359 ± 0.027	1.525 ± 0.028
*Γ* (mm s^–1^)	0.644 ± 0.042	0.436 ± 0.030	0.724 ± 0.135	0.482 ± 0.074	0.630 ± 0.050	0.474 ± 0.036	0.587 ± 0.103	0.485 ± 0.053

	240 K				300 K			
	Fe^II^		(Fe^II^ + Fe^III^)/2		Fe^II^		(Fe^II^ + Fe^III^)/2	
relative area (%)	39	21	13	27	36	31	14	19
*δ* (mm s^–1^)	1.050 ± 0.020	0.961 ± 0.007	0.716 ± 0.013	0.642 ± 0.017	0.919 ± 0.029	0.901 ± 0.008	0.663 ± 0.014	0.573 ± 0.016
*Δ* (mm s^–1^)	1.592 ± 0.050	2.528 ± 0.027	0.208 ± 0.053	1.383 ± 0.040	1.436 ± 0.060	2.254 ± 0.027	0.147 ± 0.263	1.222 ± 0.060
*Γ* (mm s^–1^)	0.664 ± 0.062	0.440 ± 0.040	0.501 ± 0.180	0.551 ± 0.065	0.577 ± 0.063	0.475 ± 0.036	0.534 ± 0.502	0.467 ± 0.131

a Legend: *δ*, isomer shift relative to α-iron; *Δ*, quadrupole splitting; *Γ*, full width at half-weight.

**Figure 14:**
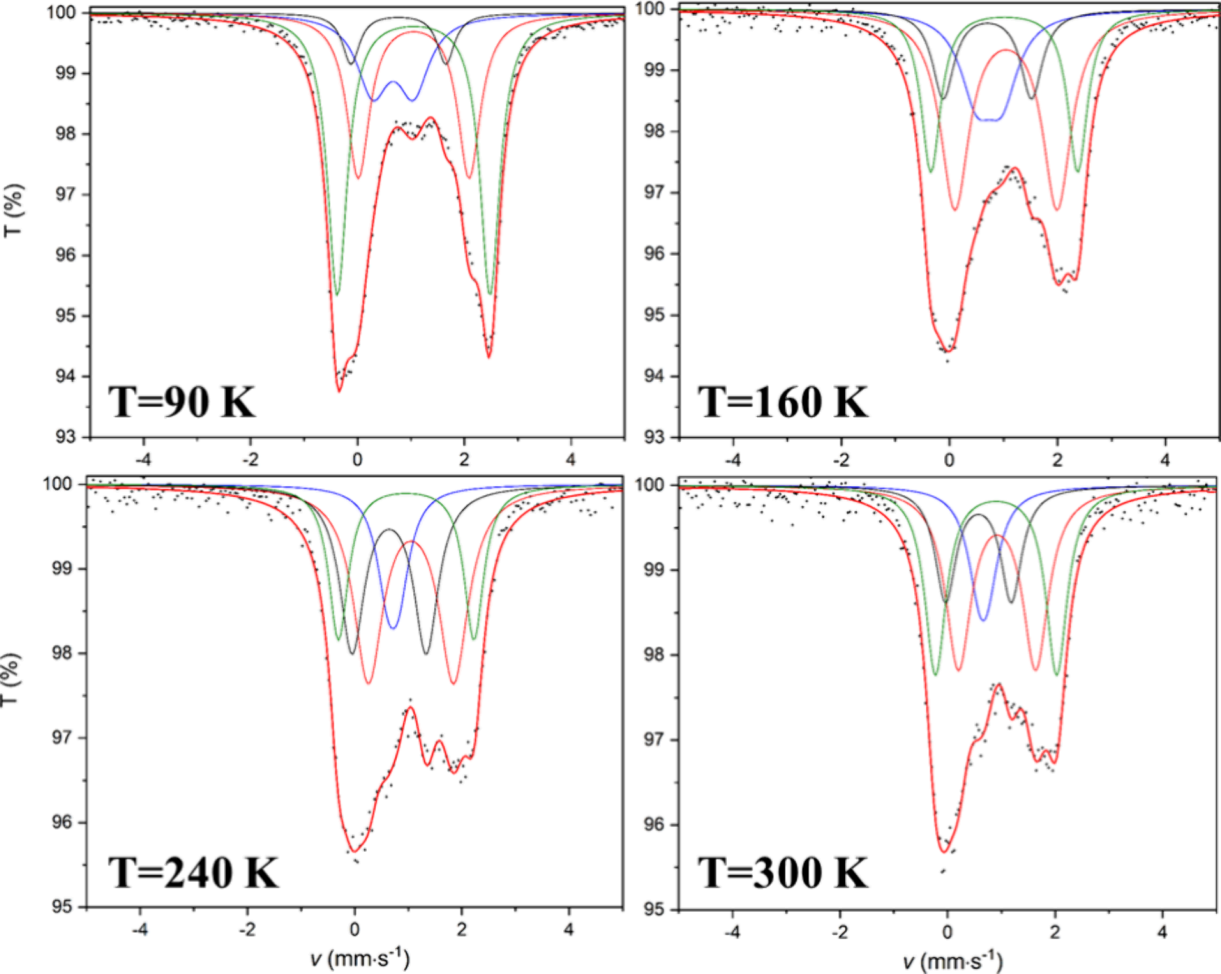
Mössbauer spectra (measured at different temperatures) of the decomposition intermediate of compound **1**, prepared in an inert atmosphere at 600 °C.

Normal chromite (FeCr_2_O_4_) has a spinel-type structure with iron(II) and chromium(III) occupying the tetrahedral [*A* (T-4)] and octahedral [*B* (OC-6)] sites, respectively.^84^ The stoichiometry of compound **1** forces an iron:chromium ratio 1:3, and the IR studies indicate a complete reduction of chromate into Cr^III^ (Figure 12) in the decomposition products. We note that the formation of chromium(II) is highly unlikely due to its reactivity. Our Mössbauer spectroscopy measurements indicate that the formal iron(II) and iron(III) contents are 87% and 13%, respectively. This is based on the following considerations. The isomer shifts of the four doublets observed in the spectra are consistent with pure Fe^III^ (two doublets) and mixed Fe^II/III^ states (the other two doublets) (Figure 14 and Table 4), with the latter being well-known in magnetite. Magnetite is an inverse spinel with Fe^III^ occupying the *A* site and Fe^II^ and Fe^III^ both occupying the *B* site (Fe^III^[Fe^II^Fe^III^]O_4_). At room temperature, an electron hopping (Fe^II^ ↔ Fe^III^) mechanism between the *B* sites is responsible for a strange isomer shift of ∼0.67 mm/s, an average of the isomer shifts that are typical for Fe^II^ and Fe^III^.^85^

In our chromite spinel, similar isomer shifts are observed for two of the four doublets (blue and black in Figure 14), and a valence state of 2.5 is assigned to these doublets. Taking into account the spectral area contributions and considering half of these doublets as Fe^II^ and the other half as Fe^III^, we obtained the formal Fe^II^ and Fe^III^ contributions as stated above. (The 90 K spectra were used for this calculation, where the possible difference between the Mössbauer–Lamb factors for the *A* and *B* sites is the lowest.)

In the inverse spinel structure of magnetite, divalent iron preferentially occupies the octahedral sites [*B* (OC-6)], resulting in mixed valence at this position, and the tetrahedral site [*A* (T-4)] is occupied by trivalent iron. In normal spinel chromite, the preference of Cr^III^ for the octahedral site is stronger than that of Fe^II^; thus, Fe^II^ is forced to take the tetrahedral sites. Because in our chromite spinel, Cr is over-stoichiometric, Cr^III^ should appear at the tetrahedral sites, too, which is therefore shared with Fe^II^ and possibly with Fe^III^ depending on the overall oxidation level of the phase. The presence of the mixed (2.5) valence states is proof that Fe^III^ is also present at the tetrahedral sites considering that electron hopping can be assumed only between identical lattice sites. In our case, the tetrahedral sites are involved, unlike in magnetite, where the octahedral Fe^II^ and Fe^III^ states participate in electron hopping.

Accordingly, on the basis of the *n*_Fe_ = 0.75 and *n*_Cr_ = 2.25 stoichiometry, the chemical formula of the single-phase spinel decomposition product can be written as (Fe^III^_0.098_Fe^II^_0.656_Cr^III^_0.250_)^T-4^(Cr^III^_2_)^OC-6^O_4+δ_, where *δ* = 0.178 (*δ* is the oxygen surplus due to the excess of trivalent metal content). However, because >4 mol of oxygen cannot be located in the spinel structure, the excess charges must be compensated with metal defects [vacancies (□)] in the structure; thus, the general formula is [(Fe^II^_0.625_(Cr,Fe)^III^_0.333_)^T-4^(Cr^III^_1.92_)^OC-6^(**□**^T-4,OC-6^_0.122_)]O_4_, supposing a homogeneous distribution of metal vacancies between the *A* and *B* spinel sites.

The typical preparation methods of chromite catalyst phases,^30,32,78,84^ which result in only ∼80% yields,^83,86^ are complicated by multistep reactions, application of harmful (NH_4_)_2_Cr_2_O_7_, high temperatures (1200–1600 °C), and long reaction times (>48 h) and also required subsequent calcination steps in H_2_, CO, and CO_2_ atmospheres. Our new method ensures phase pure chromite in 100% yield from a stable and non-explosive precursor (compound **1**) with a moderate calcination temperature (550 °C) and a short reaction time (2 h).

The defect structure and the simple preparation route make the decomposition products of compound **1** a promising candidate for catalysts in various chemical reactions, including the oxidation of benzene to phenol.^27^ It is reasonable to expect that the electron hopping behavior of the chromite phase, as concluded from our Mössbauer analysis, makes a significant contribution to the catalytic properties of this system by facilitating electron transfer mechanisms needed in redox processes.

The temperature dependence of the Mössbauer spectra of our [(Fe^II^_0.625_(Cr,Fe)^III^_0.333_)^T-4^(Cr^III^_1.92_)^OC-6^(**□**^T-4,OC-6^_0.122_)]O_4_ mixed Fe–Cr spinel revealed novel features of this potential catalyst. It has been found previously that FeCr_2_O_4_ has a cubic-to-tetragonal phase transformation between 90 and 293 K that results in a drastic change in the Mössbauer spectra; namely, the quadrupole splitting (*Δ*) of the single doublet appearing in the 90 K Mössbauer spectrum decreases from around ∼2.5 to 0.3 mm/s when room temperature is reached [while the isomer shift (*δ*) remains at ∼1.0 mm/s].^83,84^ The large *Δ* was associated with the Jahn–Teller active Fe^II^ ion at the tetrahedral position.^78,87,88^ The Mössbauer spectra of our chromium-overdoped chromite phase, obtained at 90, 120, 160, 200, 240, and 298 K (Figure 14), are different from those found in the literature for normal chromite. First, at all temperatures, the spectra can be evaluated with two sets of subspectra (doublets I and II and doublets III and IV) (Figure 14 and Table 4), representing four different chemical environments for iron. The isomer shifts of the subspectra can be separated into two sets. Doublets I and II vary between 1.1 and 1.0 mm/s, whereas with doublets III and IV varying between 90 and 298 K, the isomer shift falls between 0.8 and 0.55 mm/s (Figure S23b and Table S14). The isomer shift of doublets I and II agrees well with the high-spin iron(II) chemical environment, whereas in the case of doublets III and IV, the isomer shifts are rather consistent with a magnetite like the Fe^2.5+^ state. Furthermore, between 90 and 298 K, the quadrupole splittings start to decrease with an increase in temperature, and the slopes of the *Δ*–*T* curves for all four doublets are the same (Figure S23a). This confirms that all of the subspectra belong to the same phase. It is also obvious that despite the single-phase system, there are four different iron environments in this phase. However, only one subspectrum reaches ∼0 mm/s at 298 K, as in the report on FeCr_2_O_4_ by Tanaka et al.^83^ This subspectrum must represent a chemical environment close to that found in FeCr_2_O_4_. The presence of multiple chemical environments must be assigned to the variability of the tetrahedral nearest neighbor Fe^2+^, Fe^2.5+^, and Cr^3+^ or even a vacancy for the Fe^2+^ or Fe^2.5+^ sites. It is reasonable to assume that the Fe^2+^ showing a decrease in its quadrupole splitting to zero at room temperature (like in FeCr_2_O_4_^83,84^) has only Fe^2+^ tetrahedral nearest neighbors and no vacancies. The second Fe^2+^ species may have a tetrahedral Cr^3+^ neighbor and/or vacancies. Because electron hopping is possible between only Fe–Fe neighbors, a fraction of the tetrahedral irons (depending on the formal Fe^3+^ content) will appear as Fe^2.5+^ species. The two kinds of such species must also be due to tetrahedral Cr^3+^ neighbors or vacancies.

The presence of Cr^3+^ ions at the tetrahedral (*A*) site induces a strong Jahn–Teller effect.^88^ This means a local distortion of the lattice, and together with the effect of the vacancies, the consequence is the lack of a decrease in the quadrupole splitting to zero (it may take place above room temperature) in the case of doublets I–III. For doublet IV, the local chemical environment must be similar to the normal chromite structure (as discussed above); however, the isomer shift suggests some difference in the local electron density.

In summary, the main findings and explanations of the Mössbauer results are as follows.

(1) Nakamura et al. found an orthorhombic-to-tetragonal-to-cubic phase transition between 4 and 291 K, which resulted in a very significant change in the quadrupole splitting for normal chromite FeCr_2_O_4_.^83,84^ Due to the lack of such strong variation of *Δ*, we assume that the possible tetragonal-to-cubic phase transformation shifts to a temperature higher than 298 K in our chromium overdoped spinel. However, our cryo-temperature and high-temperature DSC studies performed between −130 and 800 °C did not show any phase transformation in this temperature range (Figure S24).
(2) Due to the 50% excess of Cr^III^ in our chromite sample, Cr^III^ is definitely positioned at the tetrahedral spinel position (*A*), too, causing Jahn–Teller distortion.^89^ Furthermore, due to the exchange of Fe^II^ for Cr^III^ and Fe^III^, cation vacancies should occur in the structure. The Jahn–Teller effect and cation vacancies can individually or jointly affect the variation of Mössbauer parameters, especially the quadrupole splitting.
(3) The isomer shifts of doublets III and IV were found to be 0.729 and 0.759 mm/s (at 90 K), respectively, which are unusual for pure iron(III) environments and suggest the formation of a virtual 2.5 oxidation state (similar to that in magnetite) due to an electron hopping behavior. The lack of a spectral change upon cooling suggests that electron hopping cannot be slowed enough to separate the iron(II) and iron(III) states even at 90 K.

Finding number 3 is supported by the lack of a wüstite-like phase in the sample prepared in a N_2_ atmosphere at 600 °C. In contrast, this phase was found by TEM in the sample prepared at 800 °C in an inert atmosphere (see below). Although further studies are necessary to determine whether our interpretations are correct, our chromite catalysts can be used, for example, in a solid oxide fuel cell or as a redox catalyst due to the high electron mobility.

The final decomposition product of compound **1** at 800 °C (in a N_2_ atmosphere) contains an iron(III)–chromium(III) mixed oxide with an eskolaite structure (ICDD-PDF 38-1479^77^) and a chromite (spinel)-like compound (ICDD-PDF 34-0140^79^) (Figure 10b).

**Figure 15:**
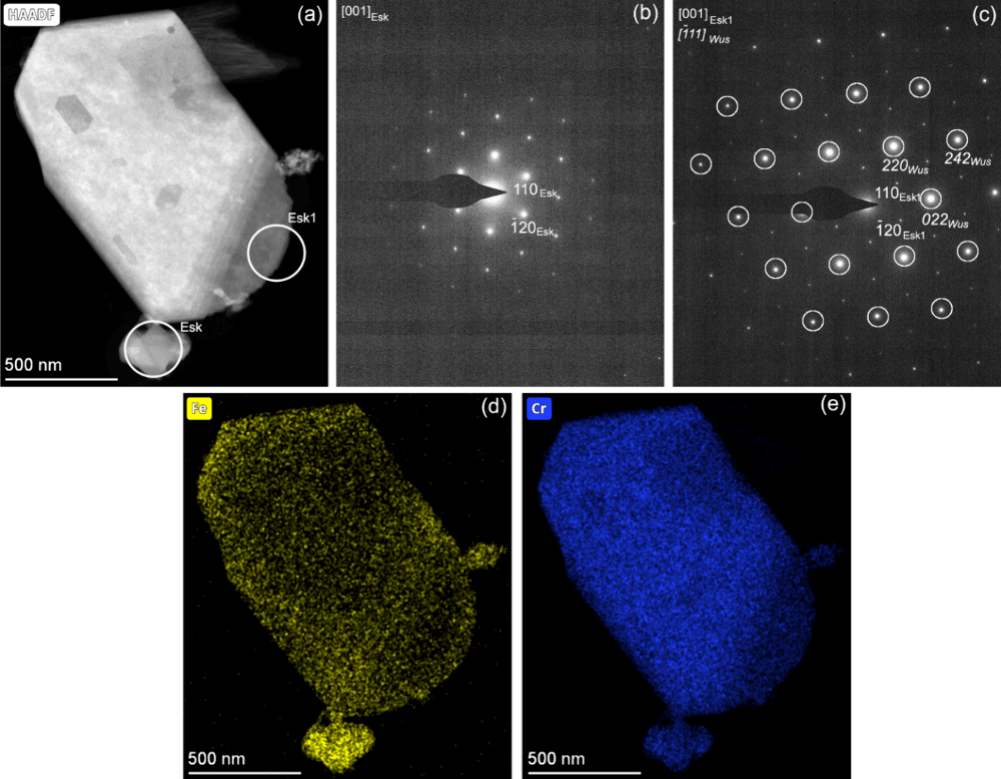
TEM results of the final mixed oxide obtained at 800 °C in a N_2_ atmosphere. (a) HAADF-STEM image, (b) SAED pattern of the grain annotated as Esk, and (c) SAED pattern from the area annotated as Esk1. White circles mark reflections that can also be interpreted with wüstite and indicate overlaps between 242(wüstite) and 330(eskolaite), 022(wüstite) and 030(eskolaite), and 220(wüstite) and 300(eskolaite) reflections. (d) Fe and (e) Cr elemental maps of the area shown in panel a.

Our TEM studies of two grains (Esk and Esk1 in panels a, d, and e of Figure 15), selected from the final thermal decomposition products, indicated the occurrence of two distinct crystal structures: homogeneous chromium and inhomogeneous iron distributions. The SAED pattern of the Esk grain was interpreted with a normal eskolaite-like lattice (Figure 15b), but the Esk1 grain (Figure 15c) revealed *d*_110_ spacings (2.63 Å) larger than those expected for eskolaite (2.48 Å), indicating the distortion of the eskolaite unit cell. Elemental maps of the grains showed homogeneous chromium (Figure 15d) and inhomogeneous iron distributions (Figure 15e). Furthermore, the SAED pattern of Esk1 (Figure 15c) showed a portion of the reflections being significantly stronger than the others. The systematically strong reflections can also be interpreted with the presence wüstite, and we interpreted the SAED pattern with the epitaxial intergrowth between distorted eskolaite, projected along [001], and wüstite, projected along ⟨111⟩ (Figure 15c). Similar intergrowth has also been observed between iron–manganese mixed oxides with a bixbyite-like Fe_2–*x*_Mn_*x*_O_3_ structure and braunite (Mn_7_SiO_12_).^90^ In the final product, we also found evidence for nanocrystalline chromite, which showed a homogeneous Fe–Cr distribution and occurred together with micrometer-sized wüstite (Figure S25).

The Mössbauer studies of the final product show an interesting subspectrum (doublet *A*) at room temperature with a δ of 0.882 mm/s and a Δ of 0.641 mm/s (Figure S26 and Table S13), which might represent the chromite-like phase identified by TEM (Figure S25) or another vacancy-containing, nonstoichiometric wüstite phase, similar to that formed by the heat treatment of [hexakis(urea)iron(III)] permanganate.^46^ There are two other subspectra, a broadened magnetic sextet and another doublet (doublet B) with a *δ* of 0.396 mm/s and a *Δ* of 0.478 mm/s (Figure S26 and Table S13). The contribution of the magnetic subspectra increased at a low temperature (90 K), whereas the contribution of doublet *B* decreased. Using the magnetic field distribution method, provided by the MossWinn code^91,92^ in the evaluation of the magnetic phase, a broad range of magnetic fields and doublet *B* with a similar isomer shift suggest that the material exhibits superparamagnetic behavior (Figure S27 and Table S13). Doublet A disappeared upon cooling. As described above, the chromite-like phase does not show magnetic splitting at 90 K; thus, doublet A rather represents the wüstite-like phase, which was identified by TEM (Figure 15 and Figure S25). Together with the SAED study that showed an epitaxial intergrowth between the eskolaite and wüstite-like phase, the Mössbauer study further strengthens our assumption. Because the eskolaite-like phase has a magnetically split spectrum at a low temperature (see below), the epitaxial intergrowth in the eskolaite–wüstite phase can be responsible for a transfer field on the iron atoms in the wüstite lattice at 90 K. The lower fields in the distribution fit must be assigned to the wüstite part where this transfer field acts on Fe^2+^ ions that have a magnetic moment that is lower than that of Fe^3+^.

**Figure 16:**
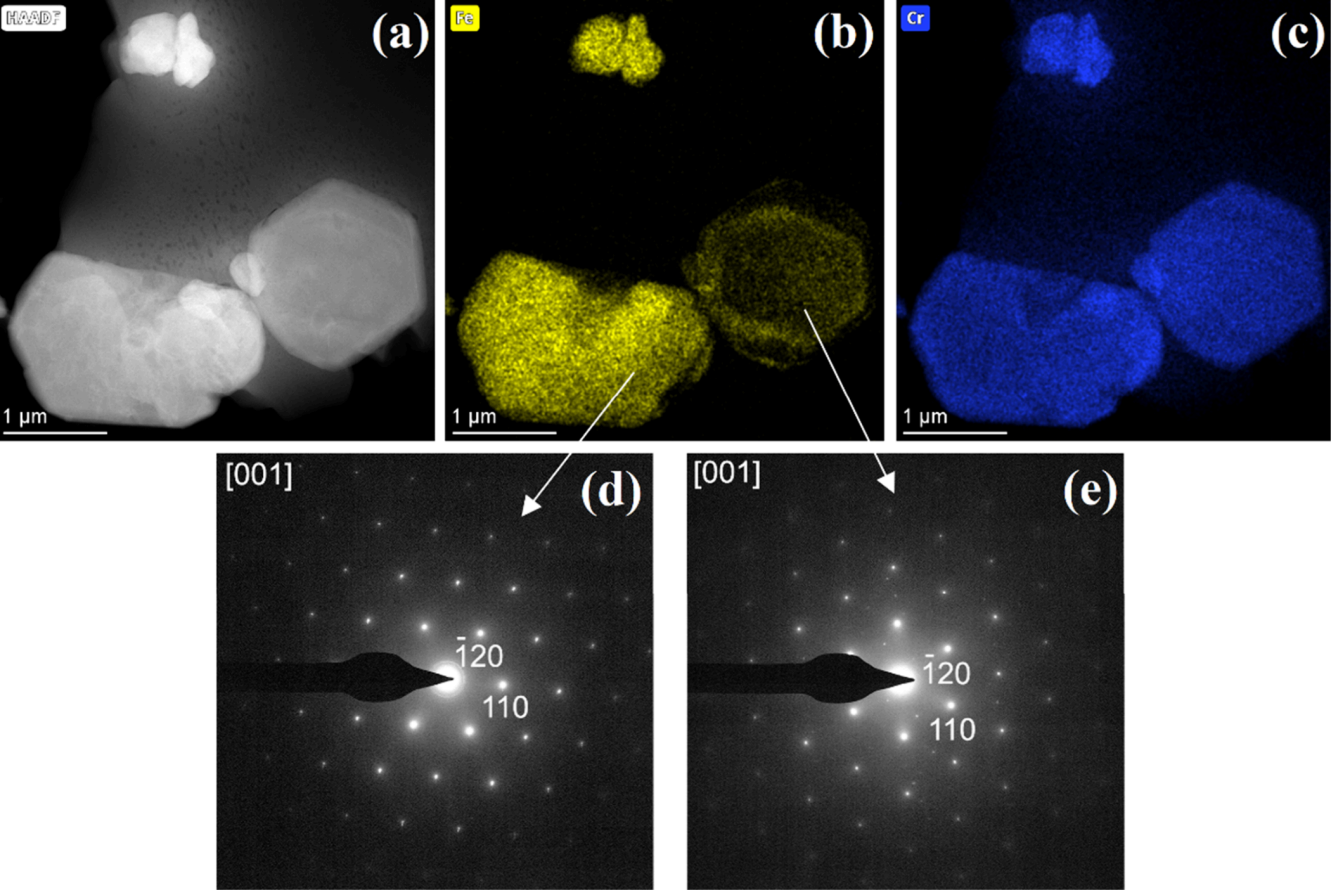
TEM results of the mixed oxide obtained from compound **1** at 800 °C in an oxidative atmosphere. (a) HAADF-STEM image and corresponding (b) Fe elemental and (c) Cr elemental maps. (d and e) SAED patterns of eskolaite projected along [001]. Weak superstructure reflections in panel e may indicate an ordering.

In an oxidative atmosphere, above 550 °C, exclusively iron–chromium mixed oxides with an eskolaite-like structure (ICDD-PDF 38-1479) formed, on the basis of PXRD and SAED measurements (Figures 10a and 16, respectively). The average grain size was 35 nm at 800 °C (based on the Scherer equation), which was confirmed by SEM (Figure 11d). According to the TEM measurements, the sample contained eskolaite grains (Figure 16a,d,e), which could be characterized by distinct iron–chromium distributions (Figure 16b,c). The SAED pattern of the low-Fe content grain (Figure 16b) reveals weak superstructure reflections indicating an ordering (Figure 16e) similar to that found by us for bixbyite prepared from hexakis(urea)iron(III) permanganate.^46^

The Mössbauer studies show the formation of iron(III)–chromium(III) mixed oxide nanoparticles with superparamagnetic behavior at 800 °C in an oxidative atmosphere. The room-temperature Mössbauer spectra consist of three subspectra (one doublet and two broadened sextets) with similar isomer shifts (0.350 mm/s), whereas the spectra, obtained at 90 K, contain only one sextet with an isomer shift of 0.456 mm/s (Figure S28 and Table S12). The isomer shifts (Table S12) reveal the iron(III) chemical environments. The disappearance of the doublet at 90 K suggests that the Fe–Cr oxide formed is superparamagnetic.

### Preliminary Result of the Catalytic Activity Test on Fe–Cr Oxides, Prepared from Compound **1** in a CO_2_ Hydrogenation Reaction

**Figure 17:**
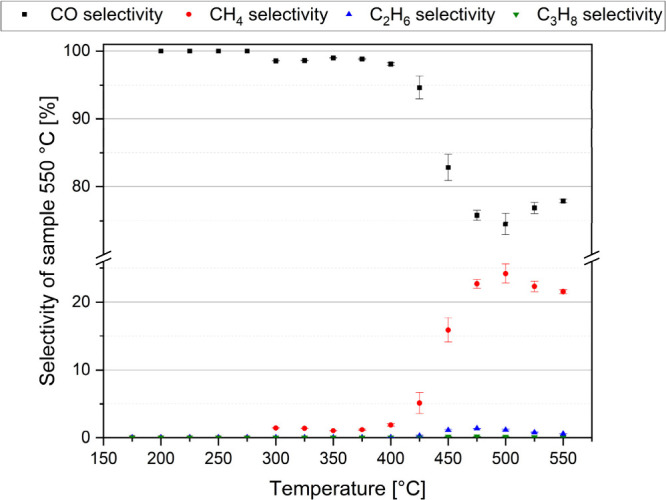
Selectivity of the different products in the CO_2_ hydrogenation reaction performed with the sample prepared at 550 °C in an oxidative atmosphere from compound **1**.

Three iron–chromium mixed oxides, prepared by the thermal decomposition of compound **1** (heating for 2 h at a particular temperature), were preliminarily tested to observe their catalytic activity in the CO_2_ hydrogenation reaction. Three intermediates were prepared in an oxidative atmosphere from compound **1**. Two were XRD amorphous [prepared at 200 and 350 °C (Figure S19)], and one had an eskolaite-like structure [prepared at 550 °C (Figure S19)]. The hydrogenation of CO_2_ was tested with a reactant gas mixture produced by simultaneous flow rates of He, H_2_, and CO_2_ of 7.5, 6, and 1.5 mL/min, respectively, at a pressure of 20 bar between 175 and 550 °C. The results are summarized in Figure 17, Table S14, and Figures S29 and S30. The conversion of CO_2_ started at ∼350 °C, and above this temperature, it resulted in yields between 50% and 62% in every experiment (Table S14 and Figures S29). Compared to our previous studies performed on Fe–Mn oxides,^46^ higher levels of conversion can be reached with the Fe–Cr oxides than with the Fe–Mn oxides, but the overall hydrocarbon selectivity is lower than that of Fe–Mn oxides. Compared to other Fe–Mn and Fe–Cr catalysts (on different carriers), the conversion was found to be better, because in those cases only ∼40% CO_2_ conversion could be reached.^16−18^ The main product was found to be carbon monoxide; however, some methane and ethane also formed, and even propane was detected in the catalytic reaction test performed with the sample prepared at 550 °C in air for 2 h (Figure 17). All of the tests were repeated five times for 1 h each, and there was no sign of loss of activity.

All catalysts were highly selective for CO (Table S14 and Figure S30); the largest amount of CO was produced with the catalyst prepared at 200 °C (>90%). However, a significant increase in selectivity for CH_4_, C_2_H_6_, and C_3_H_8_ was observed with a catalyst prepared at 550 °C from its precursors at 450 °C of the catalysis, The maximum selectivity values were ∼25% for CH_4_, ∼1.4% for C_2_H_6_, and ∼0.2% for C_3_H_8_. The observed change in the catalytic activity of the studied catalysts is probably connected to their crystallinity and the phase composition (appearance of eskolaite at 550 °C). This suggests two different mechanisms of the catalytic reactions on the amorphous and just-crystallized (eskolaite-containing) catalysts according to the two possible methods of CO_2_ adsorption: binding to the surface through the carbon atom or the two oxygen atoms. While the methanation is always mediated by adsorption through the carbon (the reduction of CO_2_ to CH_4_ is an eight-electron process with significant kinetic limitations),^93,94^ in the case of CO production, the CO_2_ molecule can be adsorbed in both ways (through carbon or two oxygens^94^). Methane formation can be attributed to the adsorption via the carbon atom of CO_2_ specifically on the eskolaite-containing samples. The large amount of CO formation does not disclose that both mechanisms, especially in the case of amorphous samples, may be present. To increase the number of sites for C-mediated adsorption, on the basis of our synthesis and catalytic test results, the high conversion activity of Fe–Cr oxides can be combined with that of Fe–Mn oxides in the future, to produce more efficient mixed oxides for applications in the conversion of CO_2_ with a selective conversion better than that obtained with the individual compounds.

## Conclusion and Future Perspectives

[Hexakis(urea-*O*)iron(III)] dichromate (compound **1**), a member of the family of versatile [M(urea)_6_]^3+^ (M = Fe, Cr, Co, Mn, Ti, or Al) complexes with oxidizing anions, was synthesized, and its crystal structure and various spectroscopic data are presented here for the first time. The solid complex is a selective mild oxidant of 2-mercaptoethanol. In particular, compound **1** can selectively (100%) oxidize 2-mercaptoethanol into the appropriate disulfide.

DSC and TG-MS studies of compound **1** showed that the complex salt decomposes upon being heated via a heat-induced solid-phase quasi-intramolecular redox reaction and yields an amorphous product, which starts to crystallize into various mixed Fe–Cr oxides. The phase relations strongly depend on the temperature and the atmosphere during the treatment, but the decomposition intermediates remain amorphous below 550 °C in both oxidative and inert atmospheres.

The single-crystal X-ray diffraction results showed that an extended hydrogen bond network is present, and the hydrogen bonds may serve as reaction centers of the heat-induced redox reaction. The composition of Fe–Cr oxides is similar to that of known catalysts, and the products of our reaction route also have catalytic activity in the hydrogenation of carbon dioxide.

The Mössbauer spectroscopy studies on the decomposition products show the formation of a structurally uncommon phase with a unique vacancy-containing composition of [(Fe^II^_0.625_(Cr,Fe)^III^_0.333_)^T-4^(Cr^III^_1.92_)^OC-6^(**□**^T-4,OC-6^_0.122_)]O_4_. In this oxide, electron hopping is recognized at the tetrahedral (*A*) sites of the spinel lattice, which opens a simple, controllable way to easily prepare not only catalysts but also SOFC electrodes. This synthesis route offers a shorter preparation time and less energy-consuming procedures. Our results can be applied to prepare solid solutions that contain different metal cations in different proportions in the cationic part, due to the isomorphism between the [Fe(urea)_6_]^3+^ and other [M(urea)_6_]^3+^ (M = Al, Mn, Cr, V, Ti, Rh, or Ir) complexes with the same anion(s). The great variability and combination of the anions can yield [(Fe, Cr, M)(urea)_6_](CrO_4_, Cr_2_O_7_, *X*O_4_, *Z*_2_O_7_, *Z*_2_O_8_)(Y) compounds with for which *X* = Mn, Re, or Cl; *Z* = S; and *Y* = a univalent anion. Our method provides a good opportunity for easy fine-tuning of the ratio of the transition metals, which is an excellent way to prepare more efficient and specific catalysts for various industrial technologies.

## Experimental Section

Chemicals [iron(III) nitrate nonahydrate, urea, sodium dichromate, unsubstituted and substituted benzyl alcohols (substituents 2-I, 2-MeO, 2-NO_2_, and 4-NO_2_), 2-octanol, 1,2-propanediol, 2-mercaptoethanol, diphenyl sulfide, hexylamine, and 3-methoxypropylamine] and the analytical reagents used in the experiments were supplied by Deuton-X Research Ltd.

### Synthesis of Compounds **1** and **1D**

[Hexakis(urea-*O*)iron(III)] dichromate was prepared as follows. Iron(III) nitrate nonahydrate (8.079 g, 0.02 mol) and urea (7.207 g, 0.12 mol) were dissolved in water (9.50 mL),^46^ and the obtained orange [hexakis(urea-*O*)iron(III)] nitrate solution was used without further purification. This solution was mixed with a solution of sodium dichromate dihydrate (9.834 g, 0.03 mol) in water (13.50 mL) at room temperature, and then the reaction mixture was left to crystallize at room temperature for 2–5 days. The mother liquor was decanted, and the orange crystals were washed successively with a copious amount of cold water, ethanol, and diethyl ether. The yield was 59%.

The iron and chromium ratio and content were determined by ICP-OS (Spectro Genesis ICP-OES), using a multielement Merck standard solution for calibration after dissolution of compound **1** in a nitric acid solution. CHN elemental analysis was performed after the ignition of compound **1** under O_2_ with GC determination of the amounts of CO_2_, H_2_O, and N_2_ using a Carlo Erba 1106 instrument. The complex was found to be anhydrous with an Fe:Cr ratio of 1:3. Elemental analysis data calculated for [Fe(urea-O)_6_]_2_(Cr_2_O_7_)_3_: C, 9.72%; H, 3.24%; N, 22.67%; Fe, 7.54%; Cr, 21.07%. Found: C, 9.56%; H, 3.30%; N, 22.67%; Fe, 7.91%; Cr, 21.29%.

Deuteration was achieved by dissolving compound **1** in the minimal amount of deuterated water, and the solution was evaporated to dryness in a desiccator over freshly prepared CaO with complete removal of the H_2_O, HDO, and D_2_O components from the solutions. This process has been repeated four times to reach an ∼70% deuteration level of compound **1**.

### Methods

The powder X-ray diffractograms were recorded with a Philips PW-1050 Bragg–Brentano parafocusing X-ray diffractometer (Cu Kα, 40 kV, 35 mA) consisting of a secondary beam graphite monochromator and a proportional counter. All scans were recorded in a step mode (0.04°/s). The calculated PXRD data were obtained with Mercury. During the calculation, we applied the same settings that we used during the PXRD measurements.

A single crystal with dimensions of 0.6 mm × 0.6 mm × 0.3 mm was picked from a crystal aggregate. The measured single crystal is shown in Figure S2. A numerical absorption correction was applied to the data. The single-crystal X-ray measurement was performed at −145 °C using Mo Kα radiation. Intensity data were collected on a RIGAKU RAXIS-RAPID diffractometer equipped with a graphite monochromator. The CCDC deposition number of compound **1** is 2366287.

The atomic positions were determined by the charge flipping method. The non-hydrogen atomic positions have been refined by anisotropic full-matrix least-squares refinement. The hydrogens were found in different Fourier maps, and the N–H bond lengths were fixed to 0.84 Å. Hydrogen atoms were included in structure factor calculations and were refined. Crystal data and details of the structure determination and refinement are listed in Table S1, atomic coordinates and equivalent isotropic displacement parameters in Table S2, hydrogen coordinates and equivalent isotropic displacement parameters in Table S3, anisotropic displacement parameters in Table S4, bond lengths and angles in Tables S5 and S6, respectively, and torsion angles in Table S7.

The Fourier transform IR and far-IR spectra of samples were measured at room temperature using Bio-Rad Digilab FTS-30-FIR and Bruker Alpha IR spectrometers (ranges of 4000–400 and 400–100 cm^–1^, respectively) in ATR (attenuated total reflection) mode.

High-resolution mass spectra (HR-MS) were obtained using a Waters Q-TOF Premier mass spectrometer in positive electrospray ionization (ESI) mode.

The UV–vis diffuse reflectance spectrum of compound **1** was measured using a Jasco V-670 UV–Vis photometer having an NV-470 integrating sphere at room temperature (standard, BaSO_4_).

The room- and low-temperature Raman measurements on compound **1** were performed at 123 and 298 K on a Horiba Jobin–Yvon LabRAM microspectrometer attached to an Olympus BX-40 optical microscope using a temperature-controlled microscope stage (Linkam THMS600). External Nd:YAG and diode laser sources (532 and 785 nm, respectively) were used at powers of ∼40 and 80 mW, respectively. The laser beam (20× objective) was focused and filtered with a D1 intensity filter in the case of 532 nm excitation to decrease the laser power and prevent the thermal decomposition of compound **1**. Light dispersion was performed using a 1000 μm confocal hole and a 1800 or 950 grooves mm^–1^ grating monochromator. The spectral ranges were between 4000 and 100 cm^–1^ with a resolution of 3 cm^–1^ and an exposure time of 40 s using 532 nm excitation and between 2000 and 200 cm^–1^ with a resolution of 4 cm^–1^ and an acquisition time of 40 s in the case of the 785 nm laser beam.

The DSC measurements were performed between −140 and 45 °C in a N_2_ atmosphere with a heating rate of 5 °C/min using a Setaram DSC92 calorimeter and a liquid N_2_ cryostat. A modified PerkinElmer DSC-3 calorimeter was used to follow the decomposition process of compound **1** between 25 and 400 °C. The heating rate was 5 °C/min (20 cm^3^/min Ar flow, ∼6 mg sample mass, and aluminum pan).

TG-MS experiments were performed using a modified PerkinElmer instrument (TGS-2) coupled to a HiQuad quadrupole mass spectrometer. Only ∼1 mg of the sample was heated at a rate of 5 °C/min to prevent explosion-like decomposition in a Pt crucible under an Ar flow rate of 140 cm^3^ min^–1^ until the temperature reached 500 °C. The ions (*m*/*z* 2–88) were monitored in SIM (selected ion monitoring) mode. To distinguish water and ammonia, the *m*/*z* 18 (water) ion intensity curve was subtracted in proportion to the MS fragmentation of water to OH (*m*/*z* 17); thus, the *m*/*z* 17 ion curve belongs to the ammonia parent ion. The *m*/*z* 28 (N_2_, CO) ion intensity curve was modified similarly taking into account the fragmentation of CO_2_ and N_2_O (*m*/*z* 44).

The GC-MS measurement parameters were given in detail previously.^46^ Briefly, a Shimadzu QP2010 SE instrument was used with a 30 m ZB-WAX PLUS capillary column, a He flow rate of 0.87 mL/min, and a split ratio of 300, from 0 to 340 °C with a heating rate of 20 °C/min (15.5 min per given temperature). The injector and interface temperatures were 300 and 325 °C, respectively. The ionization energy was 70 keV. The MS ion source temperature was 260 °C. The scanning range was *m*/*z* 10–800. The MS spectra were measured 2.3 min after the injection.

The ^57^Fe Mössbauer spectroscopy measurements were performed with a conventional Mössbauer spectrometer (Wissel, Starnberg, Germany) operating in constant acceleration mode. The source was ^57^Co in a Rh matrix. For low-temperature measurements, the samples were kept in a cryostat (SVT-400-MOSS, Janis, Woburn, MA) filled with liquid nitrogen. The isomer shifts are given relative to α-Fe at room temperature. The Mössbauer spectra were evaluated by standard computer-based statistical analysis methods that included fitting the experimental data by a sum of Lorentzians using a least-squares minimization procedure with the help of MossWinn version 4.0.^91,92^

TEM data were acquired with a 200 keV Talos Thermo Scientific transmission electron microscope. Grains were crushed under ethanol and deposited on copper grids covered with lacey carbon. We obtained bright field TEM (BFTEM) images, high-angle annular dark field scanning (HAADF-STEM) images, and selected-area electron diffraction (SAED) patterns. The elemental composition of the grains was measured with a “Super-X” detector system, having four silicon drift detectors built into the microscope column. Fast Fourier transforms (FFTs) were calculated with Gatan Digital Micrograph version 3.6.1.

SEM measurements were performed with a JEOL JSM-IT700HR instrument operating at 20 kV in low vacuum (30 Pa) and 3 kV in high vacuum (5 × 10^–7^ Pa).

### Organic Oxidation Reactions

The oxidation reactions of a given organic substrate (1.3 mmol) with compound **1** (2.6 mmol) were performed in a 250 mL round-bottom flask supplied with a reflux condenser in 30 mL of benzene as the reaction medium. The reaction mixture was stirred for 2 h at room temperature or the reflux temperature of benzene (80 °C) for 2 and 4 h. The organic compounds, formed during the oxidation reaction, were identified and determined with GC-MS, whereas the isolated yield of compounds was determined with the formation of the 2,4-dinitrophenylhydrazone precipitate. The 2,4-dinitrophenylhydrazine reagent was prepared by the dissolution of 1.2 g of 2,4-dinitrophenylhydrazine in 50 mL of 30% aqueous perchloric acid. The reagent was diluted twice with water and added to the filtered benzene solution diluted with ethanol. After benzene separation, the ethanol suspension was filtered and dried.

### Catalytic CO_2_ Hydrogenation Experiments

The catalytic reaction (CO_2_ hydrogenation) was studied in the Microactivity Effi microreactor. The pressure was set to 20 bar, and gas flows were 12, 6, and 1.5 N mL/min for He, H_2_, and CO_2_, respectively. Catalysts were studied between 175 and 550 °C (25 °C steps, 4 h at each temperature); thus, the studies were performed at 16 temperature points, all together in 64 h. The products were analyzed in hourly intervals by CG/MS (connected to the reactor, Agilent 7890B and MS - Agilent 5977B MSD).

## Supporting Information Available

HPLC-MS-CI studies of compound **1** (Figure S1); orange hexagonal prism of compound **1** (Figure S2); cryo-DSC curve of compound **1** (Figure S3); crystal data and structural refinement of compound **1** (Table S1); atomic coordinates (×10^4^) and equivalent isotropic displacement parameters (Å^2^ × 10^3^) (Table S2); hydrogen coordinates (×10^4^) and isotropic displacement parameters (Å^2^ × 10^3^) (Table S3); anisotropic displacement parameters (Å^2^ × 10^3^) (Table S4); bond lengths (Å) in compound **1** (Table S5); bond angles (deg) in compound **1** (Table S6); torsion angles (deg) compound **1** (Table S7); theoretical (calculated from SXRD measurement) and experimental PXRD of compound **1** (Figure S4); asymmetric unit of compound **1** (Figure S5); distances between the iron ions in the structure of compound **1** (Figure S6); packing of isomorphous [hexaureachromium(III)] dichromate (PEDWIG in CCSD) (Figure S7); assignment of the vibrational modes of urea ligands in compound **1** (Table S8); results of the correlation analysis for the urea ligand modes (Text S1); internal (a) and external (b) vibrational modes of the urea ligand in the complex cation of compound **1** (Figure S8); hindered translations of the Fe^III^ ion in compound **1** (Figure S9); analytical range IR spectra of compounds **1** and **1D** (Figure S10); far-IR spectra of compound **1** (Figure S11); Raman spectra of compound **1** (with a 785 nm laser) (Figure S12); Raman spectra of compound **1** (with a 532 nm laser) (Figure S13); oxidation test results of different alcohols with compound **1** (Table S9); TG, DTG, and DTA curves of compound **1** in air (Figure S14); TG and DTG curves of compound **1** in an inert atmosphere (Figure S15); TG-MS curves of compound **1** in an inert atmosphere (Figure S16); DSC curve of compound **1** in air (Figure S17); DSC curve of compound **1** in an inert atmosphere (Figure S18); *ΔH* (mJ/g) values of the oxidative and inert atmosphere decomposition reactions, determined with the DSC method (Table S10); PXRD of the heat treatment products of compound **1** in (a) oxidative and (b) inert atmospheres (Figure S19); SEAD and distribution of Cr and Fe in the heat treatment product of compound **1** in an oxidative atmosphere prepared at 325 °C (Figure S20); Mössbauer parameters of the intermediate phases formed in an oxidative atmosphere between 165 and 550 °C (Table S11); Mössbauer parameters of the intermediate phases formed in an oxidative atmosphere at 800 °C (Table S12); Mössbauer parameters of the intermediate phases formed in an inert atmosphere between 165 and 800 °C (Table S13); Mössbauer spectra of the heat treatment products of compound **1** prepared in (a) oxidative and (b) inert atmospheres at 325 °C (Figure S21); Mössbauer spectra of the heat treatment products of compound **1** prepared in an inert atmosphere at 400 °C (Figure S22); changes (with temperature) in the quadrupole splitting (QS) and isomer shift (IS) parameters of the decomposition intermediate of compound **1** prepared in an inert atmosphere at 600 °C (Figure S23); (a) cryo-temperature and (b) high-temperature DSC studies of the decomposition intermediate of compound **1** prepared in an inert atmosphere at 600 °C (Figure S24); SEAD and distribution of Cr and Fe in the heat treatment product of compound **1** in an inert atmosphere prepared at 800 °C (Figure S25); Mössbauer spectra (measured at room temperature) of the heat treatment product of compound **1** in an inert atmosphere prepared at 800 °C (Figure S26); (a) Mössbauer spectra (measured at 90 K) of the heat treatment product of compound **1** in an inert atmosphere prepared at 800 °C and (b) magnetic field distribution evaluation of the sample (Figure S27); Mössbauer spectra [measured at (a) room temperature and (b) 90 K] of the heat treatment product of compound **1** in an inert atmosphere prepared at 800 °C and (c) evaluation of the magnetic field distribution (at 90 K) (Figure S28); preliminary result of the catalytic activity test of iron–chromium mixed oxides in CO_2_ hydrogenation (Table S14); conversion of CO_2_ in hydrogenation (Figure S29); selectivity of the different catalysts in the CO_2_ hydrogenation reaction (Figure S30) (PDF)
