# Supplementary material for: Studies on a Urea-Complexed Iron(III) Dichromate, a Precursor of Chromium-Rich Nanospinel Catalysts Prepared for the Reductive Transformation of Carbon Dioxide
Source: Inorg Chem. 2025 Feb 10;64(7):3427–44. doi: 10.1021/acs.inorgchem.4c05009 (PMC11863387; doi:10.1021/acs.inorgchem.4c05009)
Supplement: Supplementary file 1 — ic4c05009_si_001.pdf [file ic4c05009_si_001.pdf]

Electronic Supplementary Material

Studies on a urea complexed iron(III) dichromate – a precursor of chromium-rich nano-spinel catalysts preparation for the reductive transformation of carbon dioxide

Kende Attila Béres[1,2], Zoltán Homonnay[1,3], Laura Bereczki[1,4],Vladimir M. Petruševski[5], Attila Farkas[6], Zsuzsanna Czégény[1], Péter Németh[7,8], Péter Pekker[8], Fanni Béres-Szilágyi[9], Tomáš Stryšovský[10], Libor Kvitek[10], Ágnes Gömöry[11], László Kótai[1,*]

[1]*Institute of Materials and Environmental Chemistry, HUN-REN Research Centre for Natural Sciences, Budapest, Hungary; beres.kende.attila@ttk.hu (K.A.B.); nagyne.bereczki.laura@ttk.mta.hu (L.B.)*

[2]*György Hevesy PhD School of Chemistry, ELTE Eötvös Loránd University, Budapest, Hungary*

[3]*Institute of Chemistry, ELTE Eötvös Loránd University, Budapest, Hungary;* homon-nay.zoltan@ttk.elte.hu

[4]*Centre for Structural Science, HUN-REN Research Centre for Natural Sciences, Budapest, Hungary*

[5]*Institute of Chemistry, Faculty of Natural Sciences and Mathematics, Ss. Cyril and Methodius University, Skopje, MK-1000, Republic of North Macedonia; vladimirpetrusevski@yahoo.com*

[6]*Department of Organic Chemistry and Technology, Faculty of Chemical Technology and Biotechnology, Budapest University of Technology and Economics, Budapest, Hungary;* farkas.attila@vbk.bme.hu

[7]*Institute for Geological and Geochemical Research, HUN-REN Research Centre for Astronomy and Earth Sciences (MTA Centre of Excellence), Budapest, Hungary; nemeth.peter@csfk.org*

[8]*University of Pannonia, Research Institute of Biomolecular and Chemical Engineering, Nanolab, Veszprém, Hungary; nanolabpekker@gmail.com*

[9]*Bay Zoltan Ltd. for Applied Research, Production Division (BAY-PROD), H-1116 Budapest, Hungary; fanni.szilagyi@bayzoltan.hu*

[10]*Faculty of Science, Department of Physical Chemistry, Palacky University Olomouc, Olomouc 77146, Czech Republic;* libor.kvitek@upol.cz

[11]*Institute of Organic Chemistry, MS Proteomics Research Group, HUN-REN Research Centre for Natural Sciences, Budapest, Hungary;* gomory.agnes@ttk.hu

**Corresponding author: László Kótai,* kotai.laszlo@ttk.hu

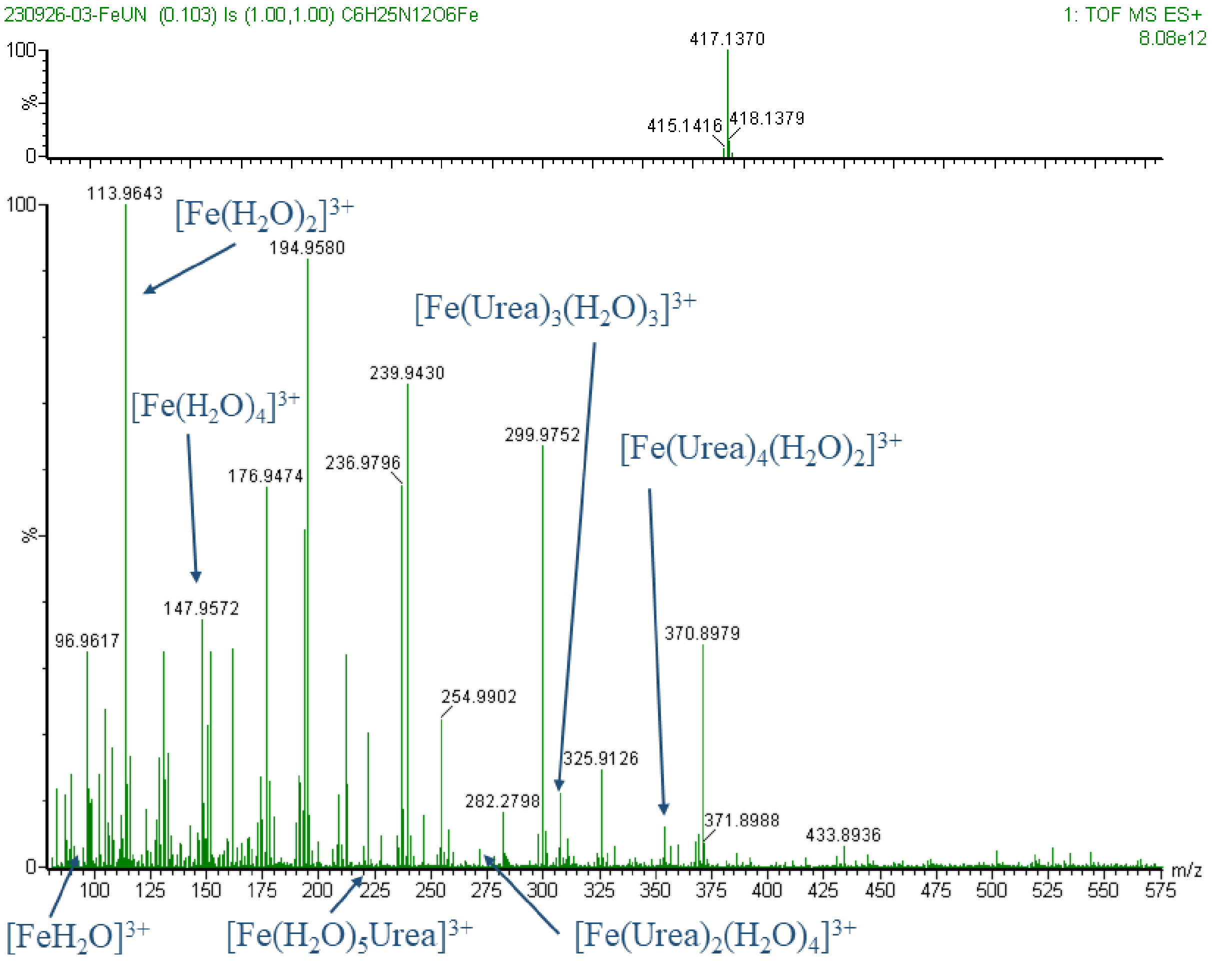

ESI Figure S1 HPLC-MS-CI studies of compound **1**. Please note, that the ionization was done by Na-formate.

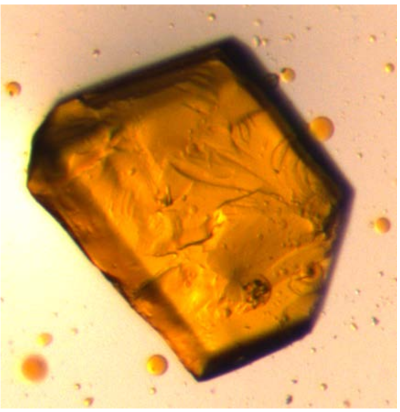

ESI Figure S2 Orange hexagonal prism of compound **1**.

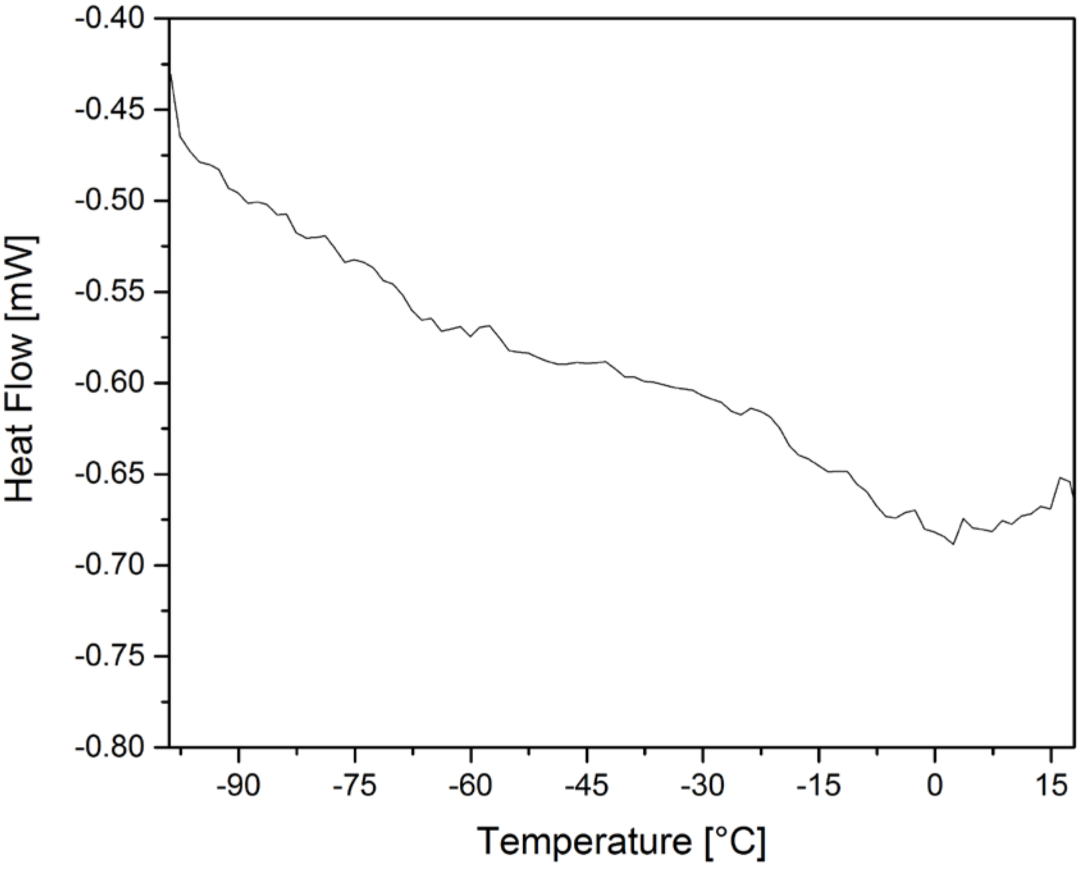

ESI Figure S3 The cryo-DSC curve of compound **1**.

ESI Table S1 Crystal data and structure refinement of compound **1**.

Empirical formula	C12 H48 Cr6 Fe2 N24 O33
Formula weight	1480.44
Temperature	128(2) K
Radiation and wavelength	Mo-Kα, λ =0.71073Å
Crystal system	Trigonal
Space group	*R* -3 c:H
Unit cell dimensions	*a* =18.6756(13)Å
	b =18.6756(13)Å
	c =27.2545(9)Å
	α =90°
	β =90°
	γ =120°
Volume	8232.2(12)$Å^3$
Z	6
Density (calculated)	1.792 Mg/m^3
Absorption coefficient, μ	1.762 mm^{-1}
F(000)	4488
Crystal colour	Yellow
Crystal description	Prism
Crystal size	0.6 x 0.6 x 0.3 mm
Absorption correction	numerical
Max. and min. transmission	0.9830.959
θ–range for data collection	$3.128 \leq \theta \leq 25.347°$
Index ranges	$-22 \leq h \leq 22;-22\leq k \leq 22;-32 \leq l \leq 32$
Reflections collected	92396
Completeness to 2θ	0.998
Independent reflections	1683 [*R*(int) =0.0790]
Reflections $I>2\sigma(I)$	1660
Refinement method	full-matrix least-squares on $F2$
Data / restraints / parameters	1683 /0 /149
Goodness-of-fit on $F2$	1.403
Final *R* indices [$I>2\sigma(I)$]	*R*1 =0.0474, *wR*2 =0.0906
R indices (all data)	*R*1 =0.0488, *wR*2 =0.0912
Max. and mean shift/esd	0.001;0.000
Largest diff. peak and hole	0.261;-0.286 e.$Å^{-3}$

ESI Table S2 Atomic coordinates (x 10^4) and equivalent isotropic displacement parameters (Å^2 x 10^3). U(eq) is defined as one third of the trace of the orthogonalized U_{ij} tensor.

	x	*Y*	*z*	*U*(eq)
Fe	0	0	1311.5(3)	23(1)
O1	571(1)	1019(1)	889.3(8)	28(1)
O2	-510(1)	493(1)	1728.7(8)	29(1)
N1	1604(2)	2199(2)	586(1)	32(1)
N2	1709(2)	1033(2)	586(1)	40(1)
N3	-1538(2)	749(2)	1874(1)	40(1)
N4	-1786(2)	-575(2)	1919(1)	39(1)
C1	1285(2)	1407(2)	693(1)	27(1)
C2	-1272(2)	219(2)	1837(1)	30(1)
Cr	562.5(3)	3460.2(3)	261.5(2)	30(1)
O3	705(2)	4386(2)	234(1)	43(1)
O4	966(2)	3333	833	31(1)
O5	1054(2)	3296(2)	-169.0(9)	39(1)
O6	-417(1)	2796(2)	231(1)	39(1)

ESI Table S3 Hydrogen coordinates (x 10^4) and isotropic displacement parameters ($Å^2$ x 10^3).

	x	*y*	*z*	*U*(iso)
H1A	1345(18)	2442(18)	663(11)	21(9)
H1B	2034(14)	2430(20)	416(12)	37(11)
H2A	2208(9)	1320(20)	505(15)	47(12)
H2B	1520(20)	540(9)	674(12)	33(10)
H3A	-1210(20)	1231(11)	1777(14)	44(12)
H3B	-2033(9)	610(20)	1911(14)	41(11)
H4A	-2291(9)	-750(20)	1963(14)	42(11)
H4B	-1600(20)	-890(20)	1892(15)	43(12)

ESI Table S4 Anisotropic displacement parameters ($Å^2 \times 10^3$). The anisotropic displacement factor exponent takes the form: $-2\pi^2(h^2a^{*2}U_{11} + ... + 2hka^*b^*U_{12})$

	U_{11}	U_{22}	U_{33}	U_{23}	U_{13}	U_{12}
Fe	20(1)	20(1)	30(1)	0	0	10(1)
O1	24(1)	25(1)	34(1)	3(1)	4(1)	14(1)
O2	24(1)	27(1)	35(1)	0(1)	5(1)	12(1)
N1	28(2)	26(2)	43(2)	6(1)	12(1)	14(1)
N2	35(2)	28(2)	59(2)	9(2)	20(2)	18(2)
N3	27(2)	40(2)	58(2)	11(2)	13(2)	21(2)
N4	29(2)	33(2)	51(2)	3(2)	11(2)	13(2)
C1	25(2)	29(2)	27(2)	-2(1)	-1(1)	12(1)
C2	29(2)	36(2)	27(2)	2(1)	2(1)	16(2)
Cr	26(1)	25(1)	40(1)	-7(1)	-7(1)	13(1)
O3	45(2)	28(1)	58(2)	-4(1)	-9(1)	20(1)
O4	26(1)	32(2)	38(2)	-6(1)	-3(1)	16(1)
O5	39(1)	38(1)	40(1)	-8(1)	-2(1)	20(1)
O6	25(1)	35(1)	55(2)	-9(1)	-11(1)	13(1)

ESI Table S5 Bond lengths (Å) in compound **1**.

Fe-O2#1	1.981(2)	Fe-O2#2	1.981(2)
Fe-O2	1.981(2)	Fe-O1#2	2.013(2)
Fe-O1#1	2.013(2)	Fe-O1	2.013(2)
O1-C1	1.275(4)	O2-C2	1.283(4)
N1-C1	1.322(4)	N2-C1	1.325(4)
N3-C2	1.314(5)	N4-C2	1.321(5)
Cr-O5	1.610(2)	Cr-O3	1.614(2)
Cr-O6	1.620(2)	Cr-O4	1.797(2)
O4-Cr#3	1.797(2)		

Symmetry codes to generate equivalent atoms:

[1] -x+y,-x,z

[2] -y,x-y,z

[3] y+1/3,x+2/3,-z+7/6

ESI Table S6 Bond angles (°) in compound **1.**

O2#1-Fe-O2#2	90.35(9)	O2#1-Fe-O2	90.35(9)
O2#2-Fe-O2	90.35(9)	O2#1-Fe-O1#2	176.29(9)
O2#2-Fe-O1#2	92.24(9)	O2-Fe-O1#2	86.98(9)
O2#1-Fe-O1#1	92.24(9)	O2#2-Fe-O1#1	86.98(9)
O2-Fe-O1#1	176.29(9)	O1#2-Fe-O1#1	90.55(9)
O2#1-Fe-O1	86.98(9)	O2#2-Fe-O1	176.29(9)
O2-Fe-O1	92.24(9)	O1#2-Fe-O1	90.55(9)
O1#1-Fe-O1	90.55(9)	C1-O1-Fe	133.4(2)
C2-O2-Fe	130.0(2)	O1-C1-N1	119.1(3)
O1-C1-N2	121.6(3)	N1-C1-N2	119.3(3)
O2-C2-N3	118.6(3)	O2-C2-N4	121.6(3)
N3-C2-N4	119.8(3)	O5-Cr-O3	111.5(1)
O5-Cr-O6	110.4(1)	O3-Cr-O6	109.6(1)
O5-Cr-O4	106.9(1)	O3-Cr-O4	109.0(1)
O6-Cr-O4	109.3(1)	Cr#3-O4-Cr	122.0(2)

Symmetry codes to generate equivalent atoms:

[1] -x+y,-x,z

[2] -y,x-y,z

ESI Table S7 Torsion angles (°) compound **1.**

Fe-O1-C1-N1	-155.7(2)	Fe-O1-C1-N2	25.3(5)
Fe-O2-C2-N3	-142.9(3)	Fe-O2-C2-N4	38.0(5)
O5-Cr-O4-Cr#1	-164.3(1)	O3-Cr-O4-Cr#1	75.1(1)
O6-Cr-O4-Cr#1	-44.7(1)		

Symmetry codes to generate equivalent atoms:

[1] -x+y,-x,z

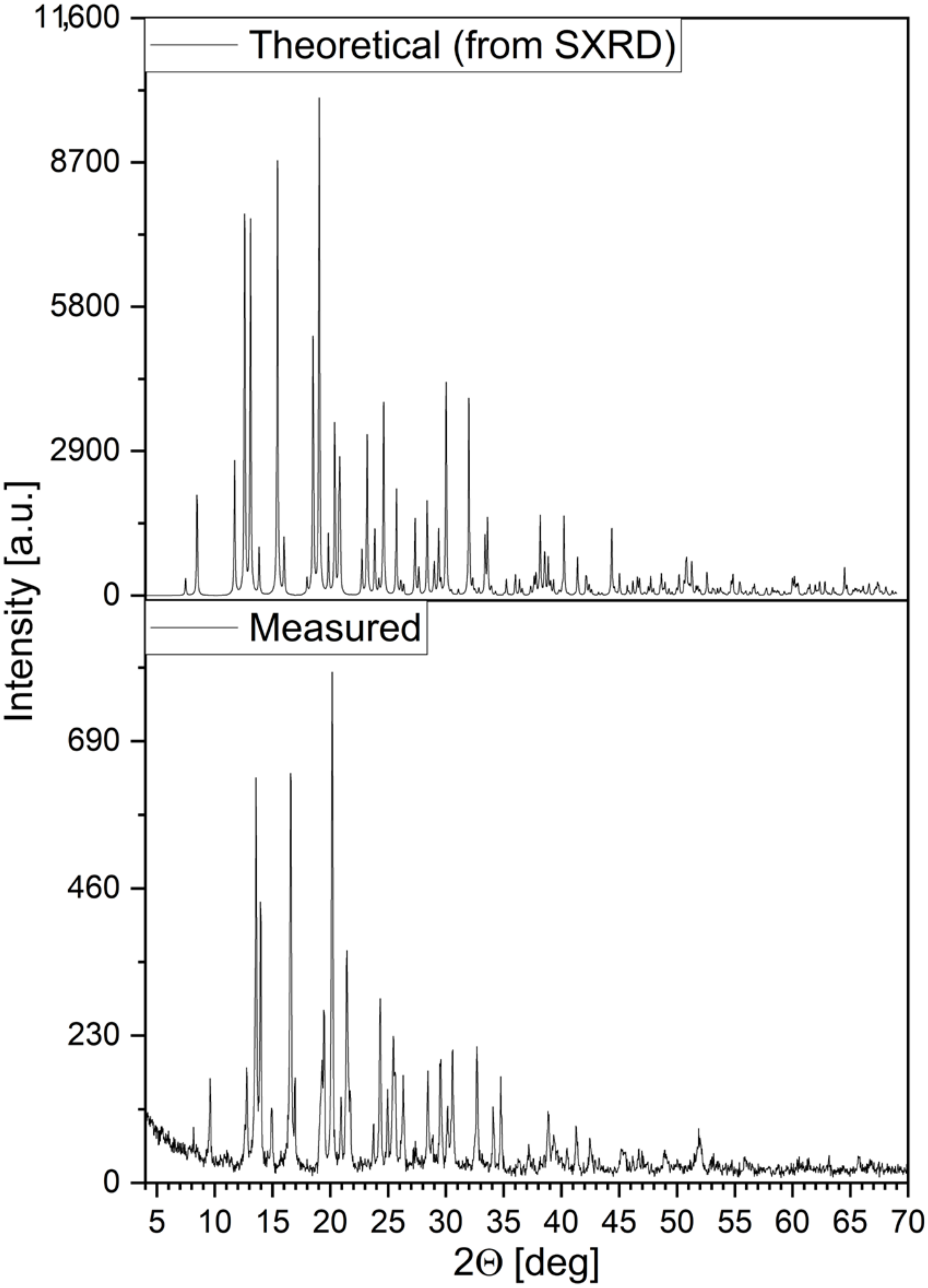

ESI Figure S4 The theoretical (calculated from SXRD measurement) and experimental PXRD of compound **1**.

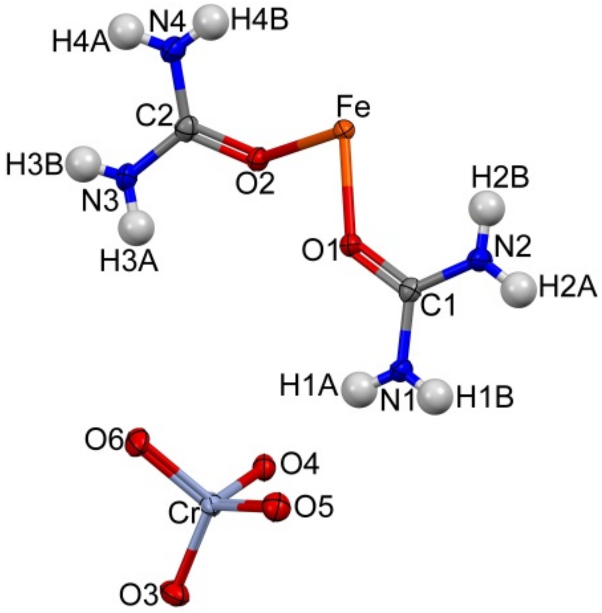

ESI Figure S5 The assymetric unit of compound **1**.

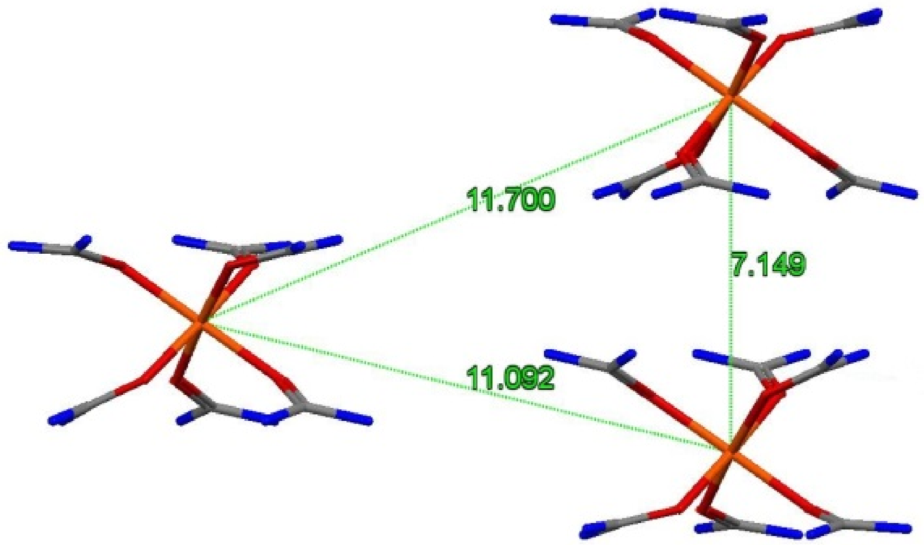

ESI Figure S6 The distances between the iron ions in the structure of compound **1**.

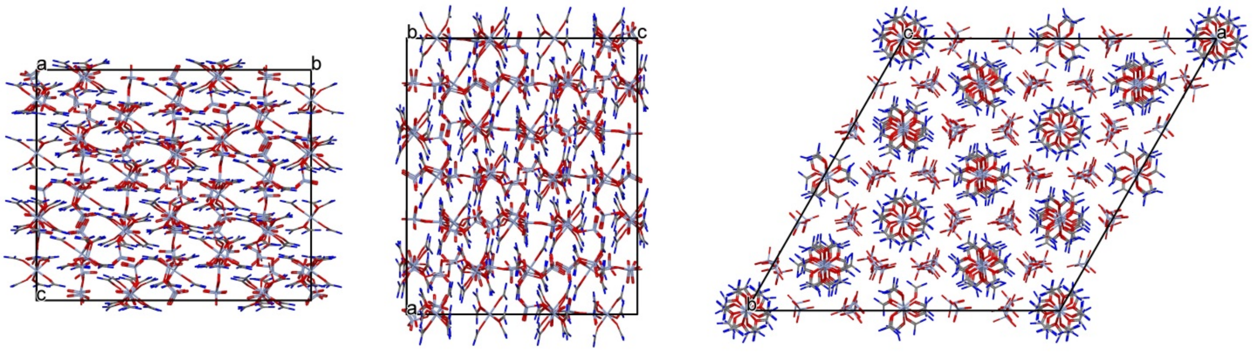

view along *a* axis　　view along *b* axis　　view along *c* axis

ESI Figure S7 The packing of the isomorphous [hexaureachromium(III)] dichromate (PEDWIG in CCSD).

ESI Table S8 The assignment of the vibrational modes of urea ligands in compound **1**.

$[Fe(urea\text{-}O)_6]_2(Cr_2O_7)_3$			Compound **1-D**	Solid urea*	Gaseous urea*	
**IR, 298 K**	**Raman, 298 K (532 nm)**	**Raman, 123 K (532 nm)**	**IR, 298 K**	**IR**	**IR**	**Assignment**
3450sh, 3412	3419	-	2598	3450, 3444	3548, 3548	$\nu_{as}(NH_2)$
3309	3350	-	2573	3349, 3341	3442,3437	$\nu_s(NH_2)$
3225	-	-	2433sh, 2413,2358			
-	-	-	1266,1288	1683	1592	$\delta_s(NH_2)$
1617	1616	-	1222	1625	1603	$\delta_{as}(NH_2)$
1539, 1532	1540	1532	1567,1556sh	1601	1735	$\nu(C=O)$
1503	1499,1461	1502	1516	1466	1388	$\nu_{as}(C\text{-}N)$
1140	1136		905	1153	1155	$\rho_s(NH_2)$
1031	1034	1052, 1038	731	1057	1029	$\rho_{as}(NH_2)$
-	-	-	998	1003	936	$\nu_s(CN)$
780sh	-	769	766sh	789	786	$\pi(CO)$
711	743	757	562	721	517	$\tau_{as}(NH)$
604	607	607	562	569	568	$\delta(C=O)$
526	-	511	550	530	467	$\delta(C=O)$, $\delta(C\text{-}N)$
495,471	477	475	450	509	423	$\omega_{as}(NH_2)$, together with $\delta(C=O$

* Keuleers et al.[33]

ESI Text S1: The results of the correlation analysis for the urea ligand modes

Urea (C_{2v} symmetry) has 18 internal vibrations: $7A_1$, $2A_2$, $3B_1$ and $6B_2$. The 24 urea molecules of the primitive cell occupy two sets of general symmetry positions (C_1 symmetry). Thus, all vibrational modes of urea, under the static field due to the local symmetry group, will be of *A* symmetry, and further give sextets due to resonant splitting of the identical oscillators in the D_{3d} unit-cell group, which is isomorphous with the factor group of the space group *R*–3*c*. Thus, it will give rise to 144 vibrations as a result of the “factor-group” splitting, equivalent to 216 vibrational degrees of freedom due to internal vibrations. For two distinct types of urea molecules, it is 288 unit-cell group vibrations, i.e. 432 vibrational degrees of freedom.

Since in the primitive cell there are 12 urea molecules of each type, the external modes of urea transform giving rise to *f-g* octets (resonant splitting) for each mode from the local group. That means 12 vibrational degrees of freedom for each hindered mode, or 36 degrees of freedom for hindered translations and 36 for hindered rotations. For the two crystallographically distinct types of urea molecules, it results in 144 unit-cell group vibrations due to external vibrations of urea.

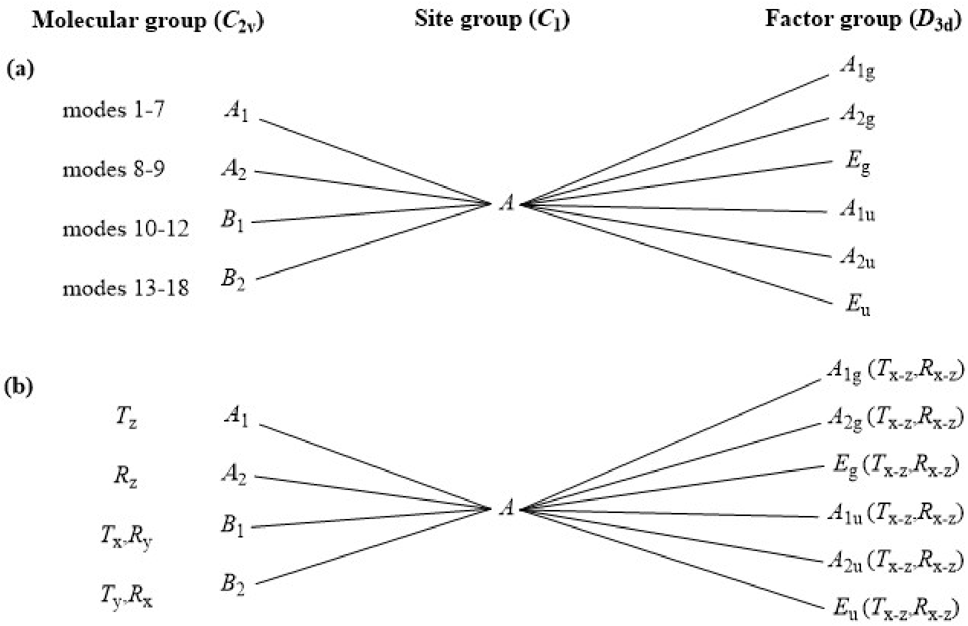

ESI Figure S8. Internal **(a)** and external **(b)** vibrational modes of urea ligand in the complex cation of compound **1**.

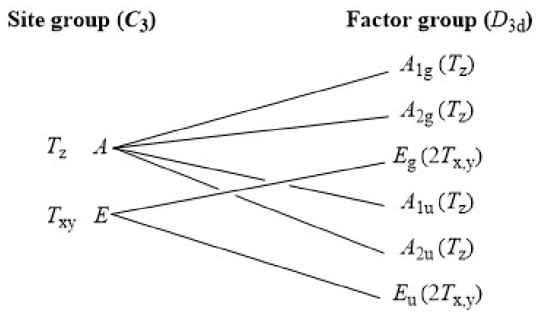

ESI Figure S9. The hindered translations of the Fe^{III} –ion in compound **1**.

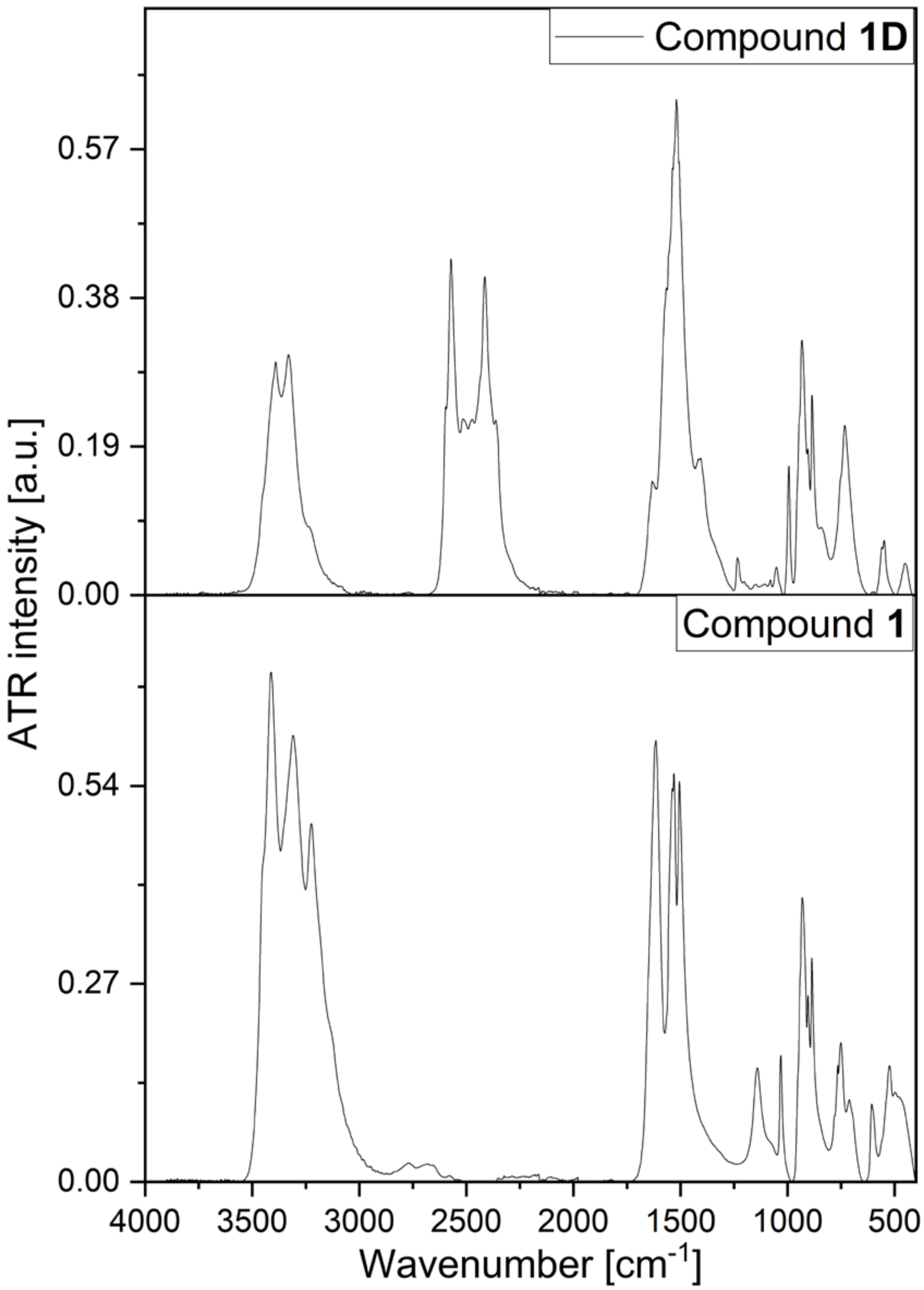

ESI Figure S10 The analytical range IR spectra of compound **1** and **1D**.

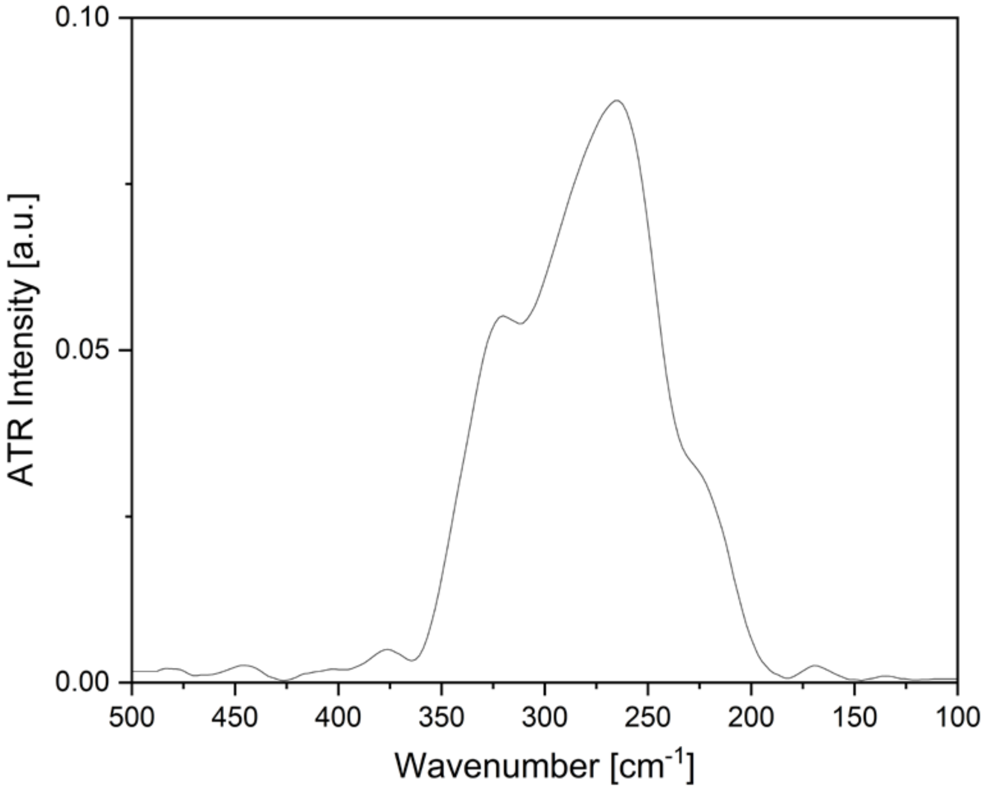

ESI Figure S11 The far range IR spectra of compound **1**.

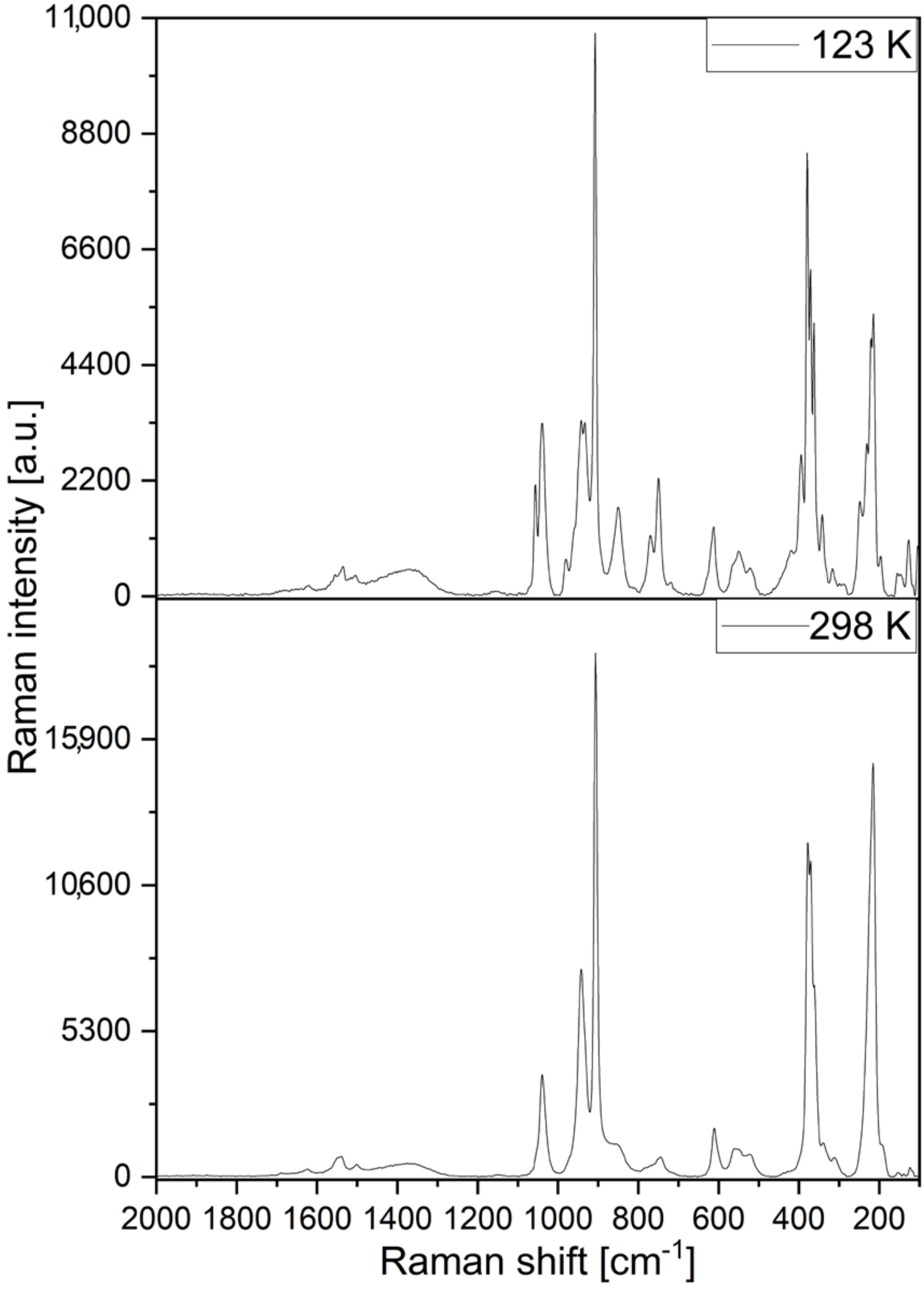

ESI Figure S12 The Raman spectra of compound **1** (with 785 nm laser).

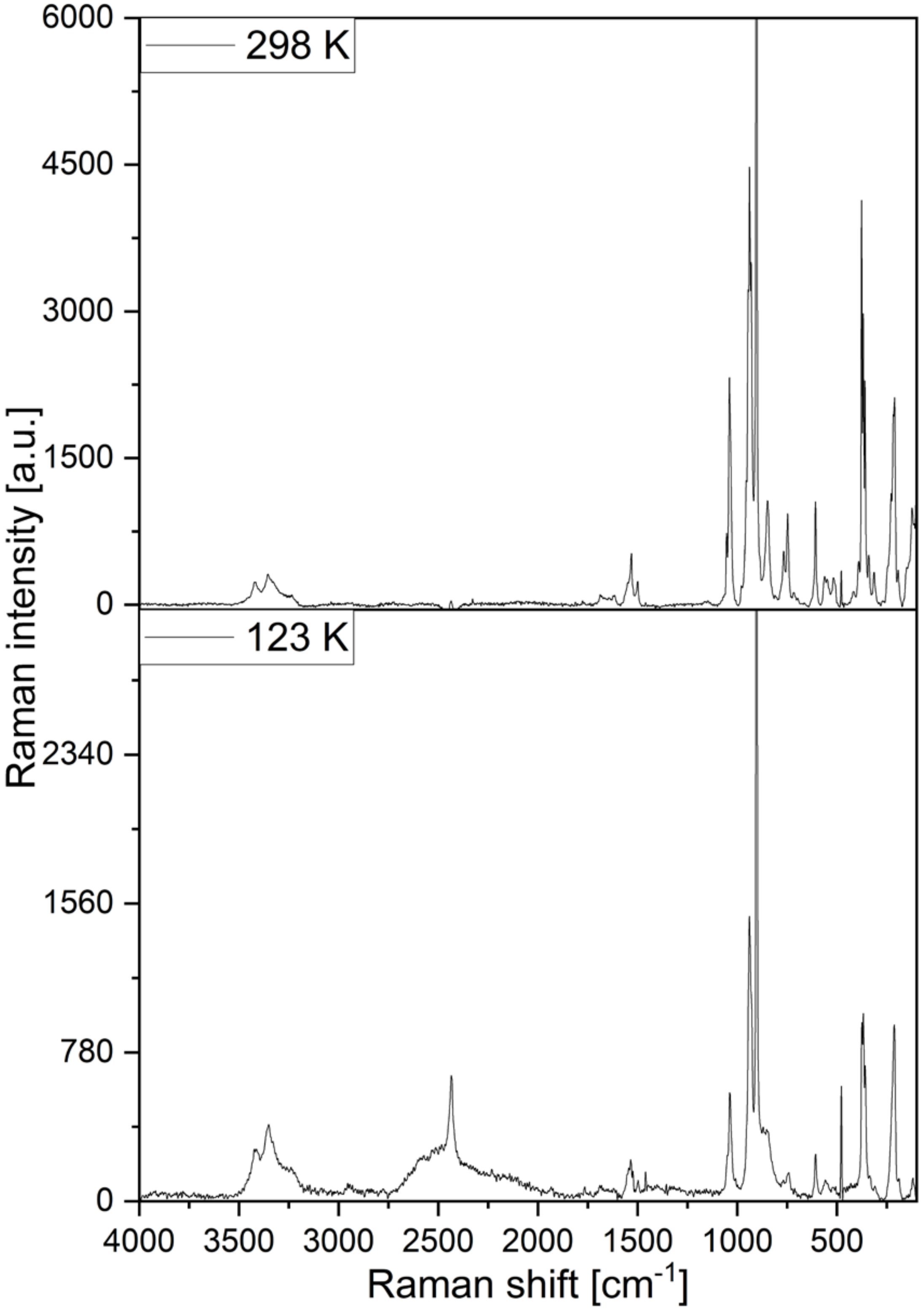

ESI Figure S13 The Raman spectra of compound **1** (with 532 nm laser).

ESI Table S9 Oxidation test results of different alcohols with compound **1**.

Substrate	*T*, °C	*t*, h	Conversion (%)		
			Aldehyde	***Alcohol***	***Ketone***
benzyl alcohol	RT	2	7.55	92.45	0
	Reflux	2	15.64	84.36	0
		4	21.58	78.35	0
2-Iodobenzyl alcohol	RT	2	7.66	92.34	0
	Reflux	2	6.68	93.32	0
		4	6.13	93.09	0
2-nitrobenzyl alcohol	RT	2	3.79	96.21	0
	Reflux	2	4.78	95.22	0
		4	9.47	90.53	0
2-nitrobenzyl alcohol	RT	2	4.78	95.22	0
	Reflux	2	7.11	92.89	0
		4	8.68	91.31	0
2-methoxybenzyl alcohol	RT	2	7.45	90.83	0
	Reflux	2	5.48	93.11	0
		4	8.96	89.17	0
2-Octanol	RT	2	0	91.01	9.1
	Reflux	2	0	89.51	10.49
		4	0	90.9	12.99

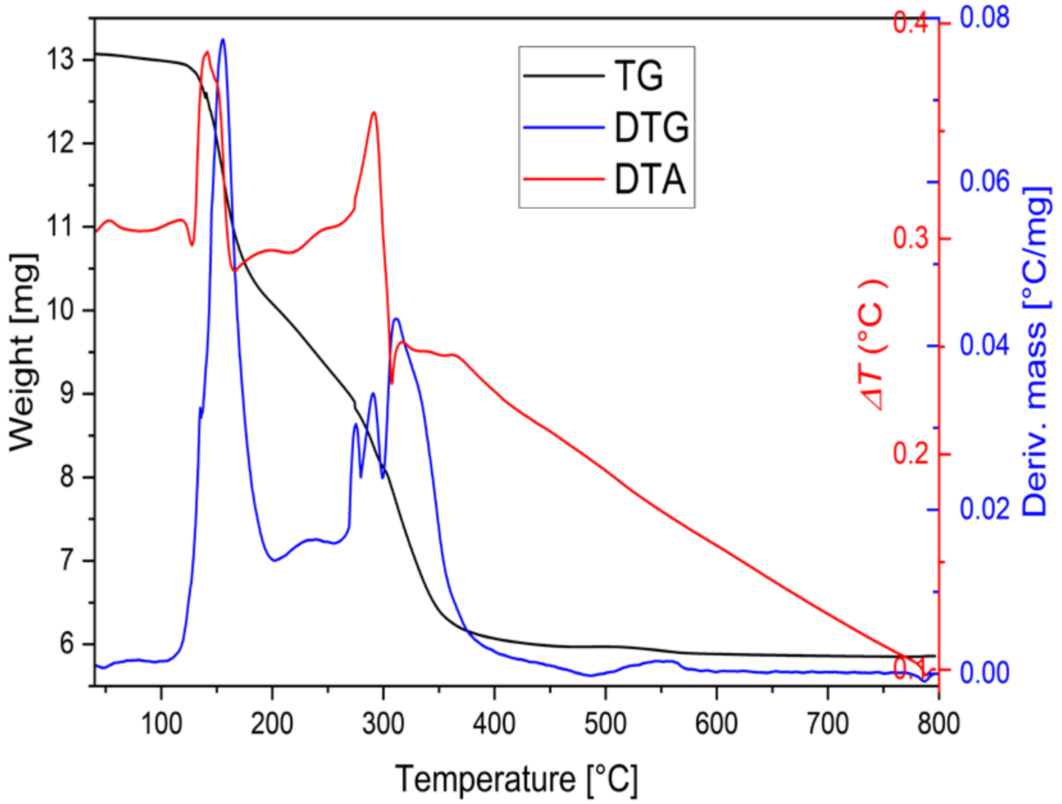

ESI Figure S14 The TG, DTG, and DTA curves of compound **1** in air**.**

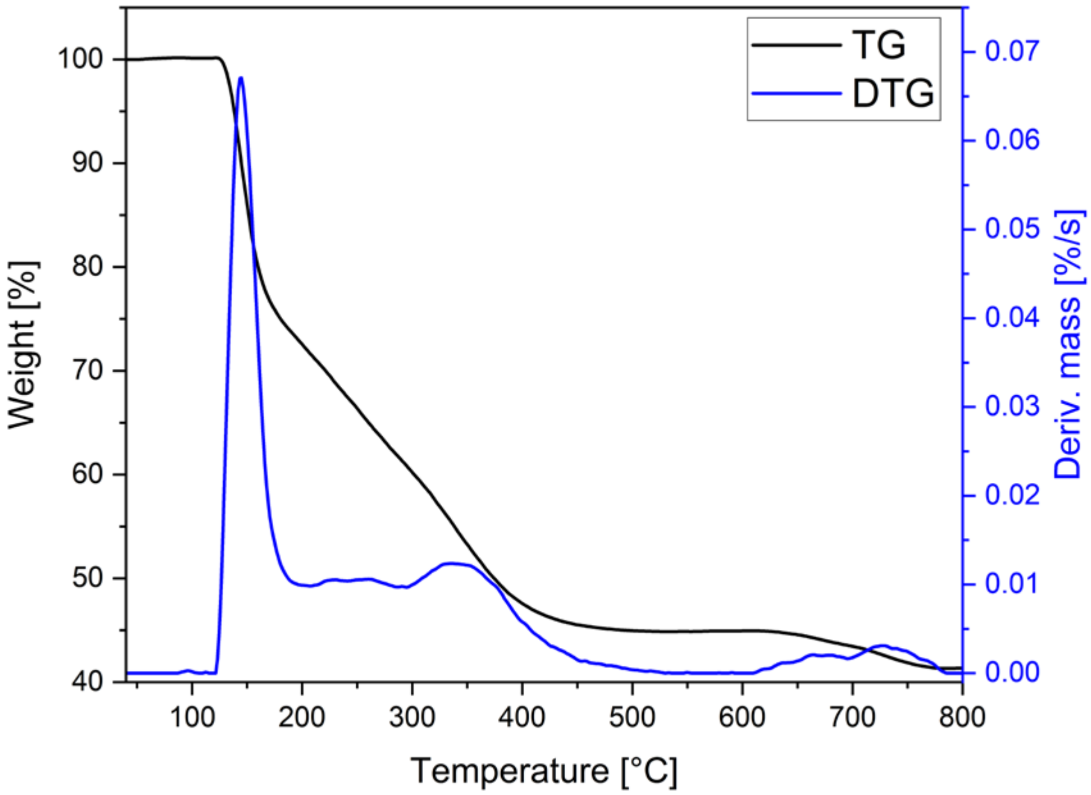

ESI Figure S15 The TG and DTG curves of compound **1** in an inert atmosphere**.**

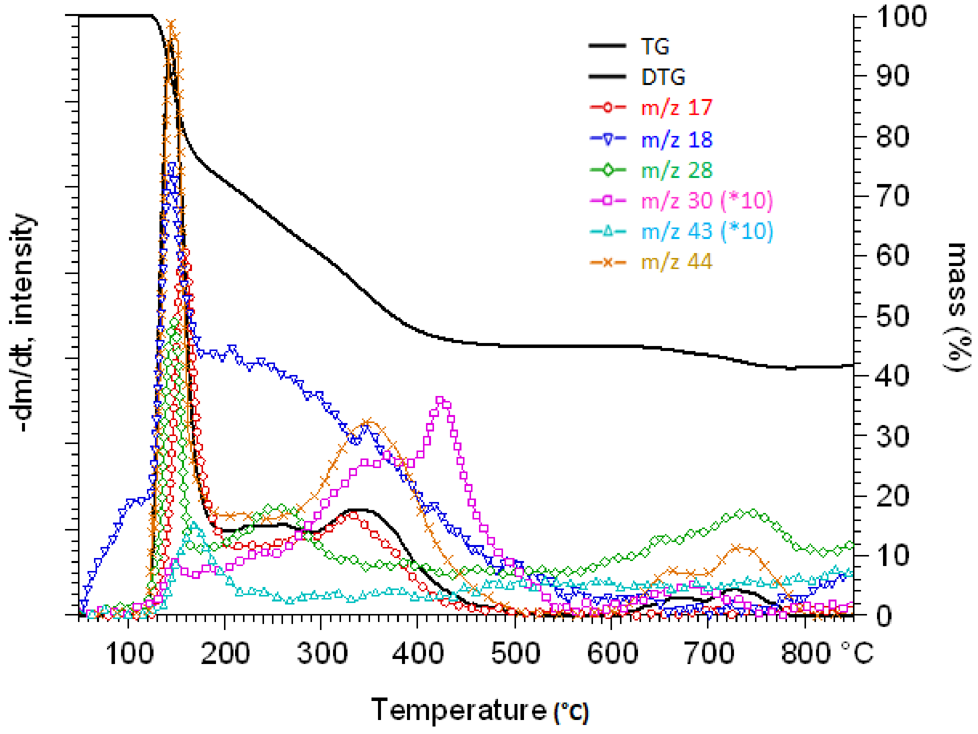

ESI Figure S16 The TG-MS curves of compound **1** in an inert atmosphere.

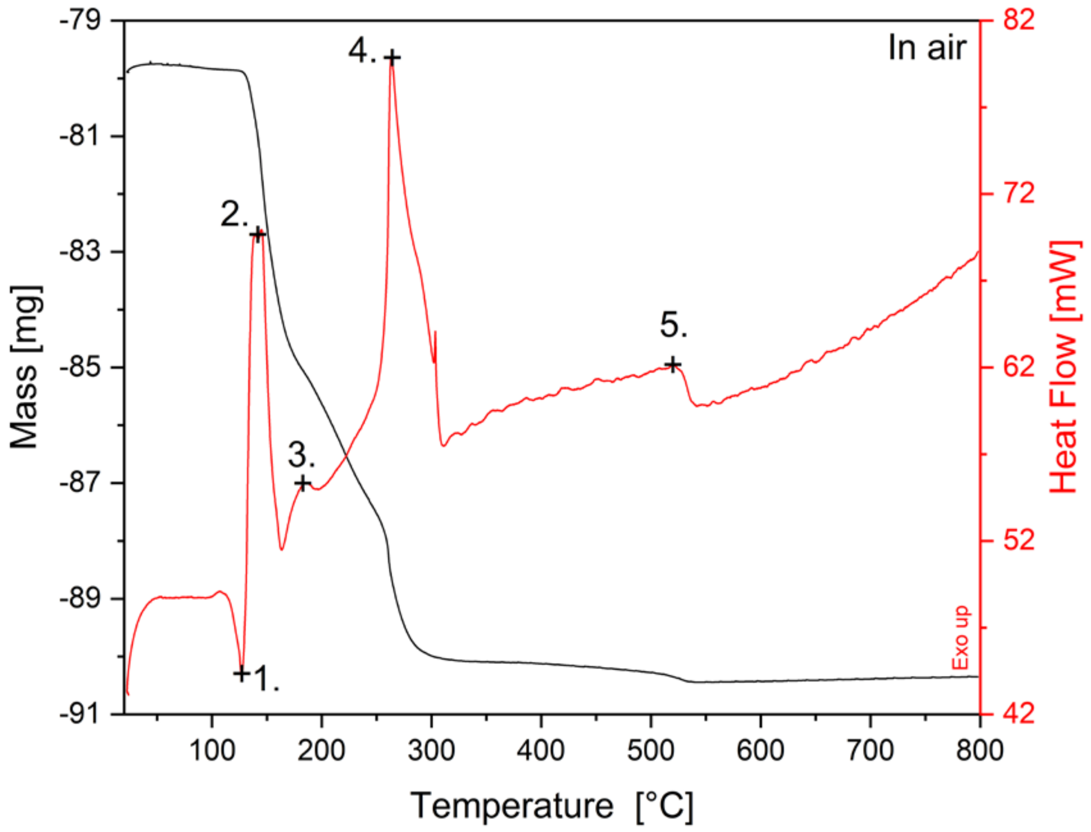

ESI Figure S17 The DSC curve of compound **1** in air.

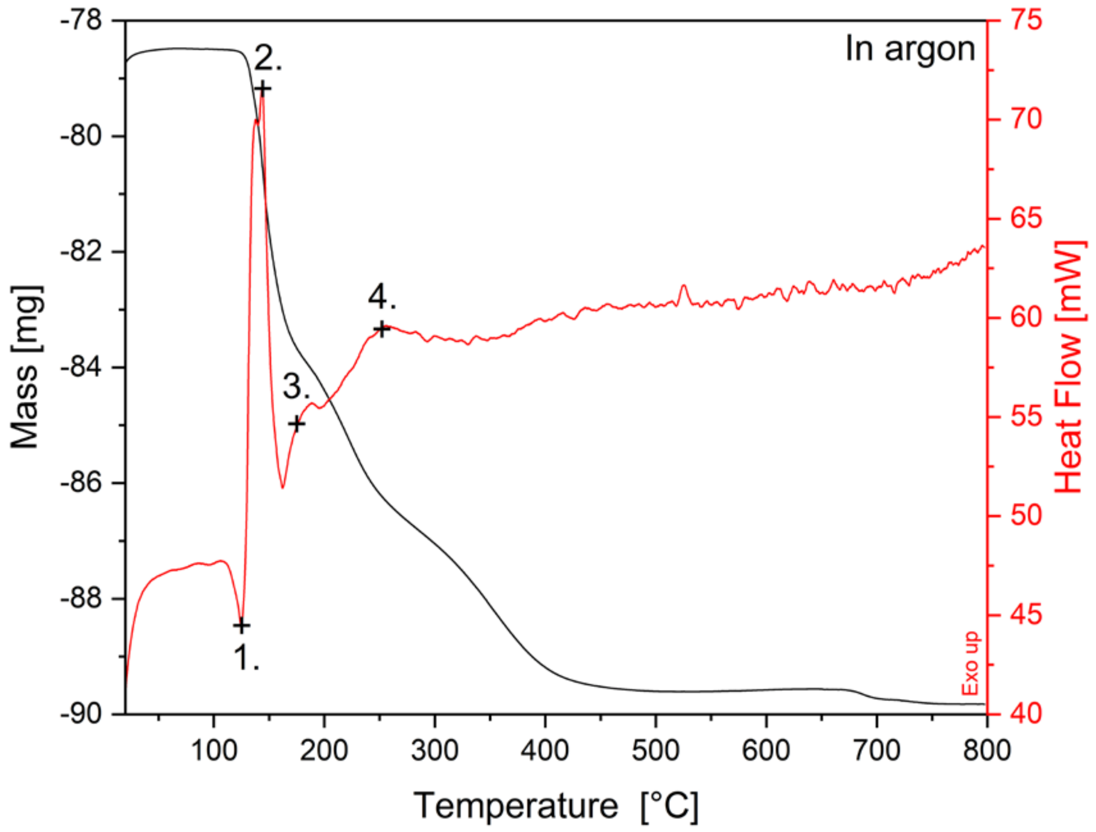

ESI Figure S18 The DSC curve of compound **1** in an inert atmosphere**.**

ESI Table S10 The *ΔH* (mJ/g) values of the oxidative and inert atmosphere decomposition reactions, were determined with the DSC method**.**

Steps	1	2	3	4	5
			In air		
T (°C)	115-131	131-163	166-196	199-310	493-537
ΔH (mJ/g)	-33.965	371.297	33.681	766.466	40.638
			In argon		
T (°C)	112-128	128-163	163-196	201-292	---
ΔH (mJ/g)	-23.686	398.205	38.111	103.711	---

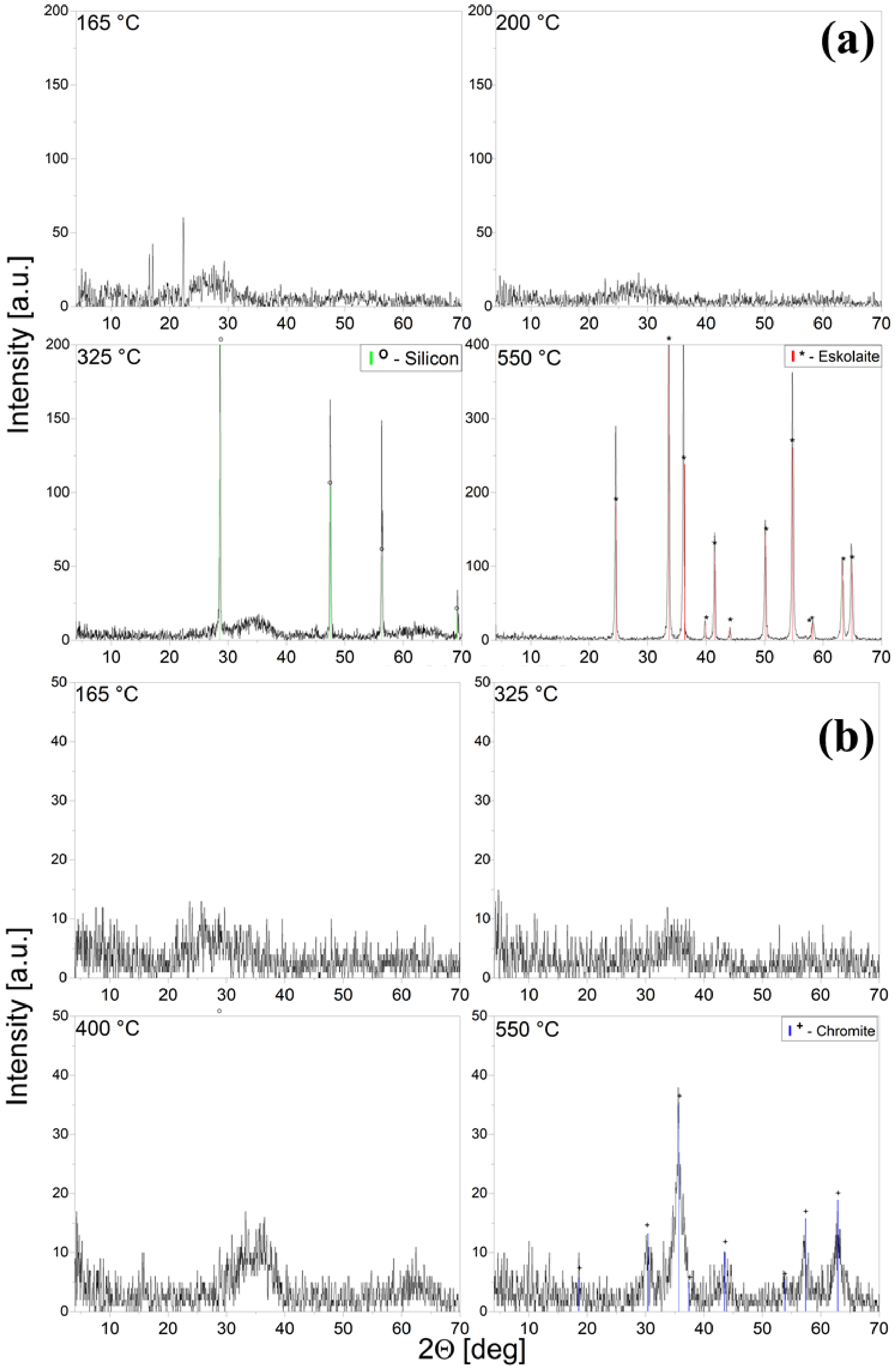

ESI Figure S19 The PXRD of the heat treatment products of compound **1** in **(a)** oxidative and **(b)** inert atmosphere**.**

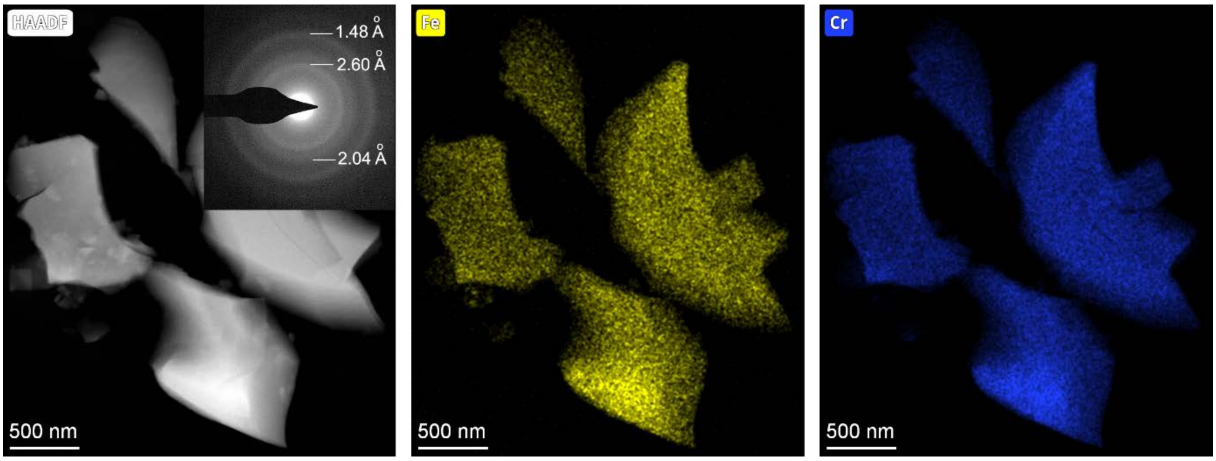

ESI Figure S20 The SEAD and the distribution of the Cr and Fe in the heat treatment product of compound **1** in an oxidative atmosphere prepared at 325 °C**.**

ESI Table S11 The Mössbauer parameters of the intermediate phases formed in an oxidative atmosphere between 165 °C-550 °C.

	165 °C			200 °C			350 °C	
Component	**Fe^{III}-tet.**		**Fe^{III}-oct.**	**Fe^{III}-tet.**		**Fe^{III}-oct.**	**Fe^{III}-tet.**	
Relative area (%)	25	39	36	38	40	22	54	46
$\boldsymbol{\delta}$ ($mm \cdot s^{-1}$)	0.341 (±0.002)	0.343 (±0.011)	0.347 (±0.012)	0.338 (±0.002)	0.343 (±0.001)	0.343 (±0.002)	0.334 (±0.003)	0.328 (±0.003)
Δ ($mm \cdot s^{-1}$)	1.437 (±0.029)	1.024 (±0.012)	0.657 (±0.019)	1.366 (±0.045)	0.956 (±0.020)	0.578 (±0.035)	1.530 (±0.027)	0.932 (±0.021)
Γ ($mm \cdot s^{-1}$)	0.376 (±0.022)	0.354 (±0.056)	0.352 (±0.017)	0.281 (±0.016)	0.367 (±0.095)	0.318 (±0.045)	0.567 (±0.023)	0.480 (±0.020)

	450 °C		550 °C	
Component	**Fe^{III}-tet.**		**Eskolaite**	**Fe^{III}-oct. (SPM)**
Relative area (%)	41	59	69	31
$\boldsymbol{\delta}$ ($mm \cdot s^{-1}$)	0.333 (±0.005)	0.313 (±0.004)	0.363 (±0.010)	0.347 (±0.001)
Δ ($mm \cdot s^{-1}$)	1.583 (±0.034)	0.978 (±0.027)	-0.235 (±0.022)	0.388 (±0.003)
Γ ($mm \cdot s^{-1}$)	0.517 (±0.0344)	0.541 (±0.023)	0.415 (±0.026)	0.309 (±0.003)
B (T)	---	---	30.0	---

ESI Table S12 The Mössbauer parameters of the intermediate phases formed in an oxidative atmosphere between 800 °C.

**Measurement temperature**	298 K		
Component	**Fe^{III}-oct. (SPM)**	**Eskolaite**	
Relative area (%)	28	70	30
δ ($mm·s^{-1}$)	0.356 (±0.002)	0.345 (±0.011)	
Δ ($mm·s^{-1}$)	0.367 (±0.004)	-0.279 (±0.023)	
Γ ($mm·s^{-1}$)	0.340 (±0.002)	0.413 (±0.027)	
B (T)	------	36.0 (±1.0)	44.6 (±0.3)
**Measurement temperature**	90 K		
Component	------	**Eskolaite**	
Relative area (%)		56	44
δ ($mm·s^{-1}$)		0.455 (±0.003)	
Δ ($mm·s^{-1}$)		-0.230 (±0.006)	
Γ ($mm·s^{-1}$)		0.361 (±0.009)	
B (T)		47.6 (±0.7)	50.6 (±0.4)

ESI Table S13 The Mössbauer parameters of the intermediate phases formed in an inert atmosphere between 165 °C-800 °C.

	**165 °C**			**250 °C**			**325 °C**			
Component	**Fe^{III}-tet.**		**Fe^{III}-oct.**	**Fe^{III}-tet.**		**Fe^{III}-oct.**	**Fe^{II}**	**LS-Fe^{III}**	**Fe^{III}-tet.**	
Relative area (%)	30	39	31	32	41	27	6	34	20	40
δ (mm·s^{-1})	0.331 (±0.004)	0.338 (±0.002)	0.340 (±0.003)	0.321 (±0.004)	0.338 (±0.002)	0.330 (±0.004)	0.625 (±0.062)	0.217 (±0.028)	0.347 (±0.013)	0.433 (±0.020)
Δ (mm·s^{-1})	1.442 (±0.054)	1.030 (±0.026)	0.676 (±0.044)	1.656 (±0.044)	1.171 (±0.023)	0.772 (±0.032)	2.589 (±0.102)	1.106 (±0.039)	1.787 (±0.067)	1.118 (±0.035)
Γ (mm·s^{-1})	0.380 (±0.043)	0.333 (±0.109)	0.326 (±0.041)	0.418 (±0.037)	0.355 (±0.084)	0.316 (±0.038)	0.517 (±0.163)	0.435 (±0.045)	0.416 (±0.091)	0.415 (±0.041)

	**400 °C**					**800 °C**				
T (K)	**298 K**					**298 K**			**90K**	
Component	**Fe^{II}**		**LS-Fe^{III}**	**Fe^{III}-tet.**		**Eskolaite like phase**				
Relative area (%)	10	9	21	8	52	28	54	18	84	16
δ (mm·s^{-1})	0.661 (±0.025)	0.718 (±0.014)	0.248 (±0.012)	0.412 (±0.024)	0.495 (±0.016)	0.386 (±0.039)	0.882 (±0.010)	0.390 (±0.008)	0.449 (±0.006)	0.464 (±0.003)
Δ (mm·s^{-1})	2.199 (±0.076)	2.711 (±0.055)	1.075 (±0.027)	1.843 (±0.068)	1.097 (±0.027)	----	0.638 (±0.013)	0.481 (±0.017)	-0.151 (±0.009)	0.445 (±0.005)
Γ (mm·s^{-1})	0.441 (±0.149)	0.393 (±0.068)	0.382 (±0.038)	0.369 (±0.139)	0.553 (±0.022)	$\Gamma_{1,6}$=0.842 $\Gamma_{2,5}$=1.412 $\Gamma_{1,6}$=2.540	0.770 (±0.016)	0.474 (±0.024)	0.365 (±0.016)	0.284 (±0.012)
B (T)	---					44.1 (±0.3)	----	----	B_1=48.5 B_2=39.6	----

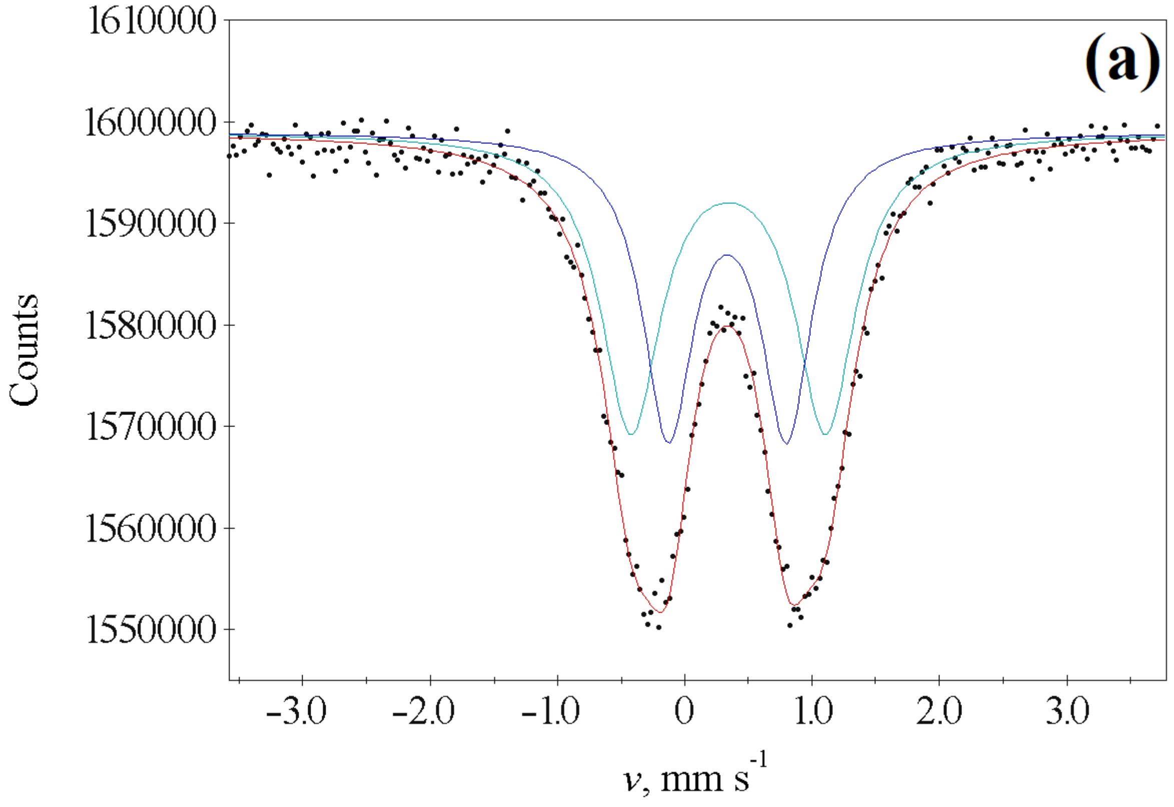

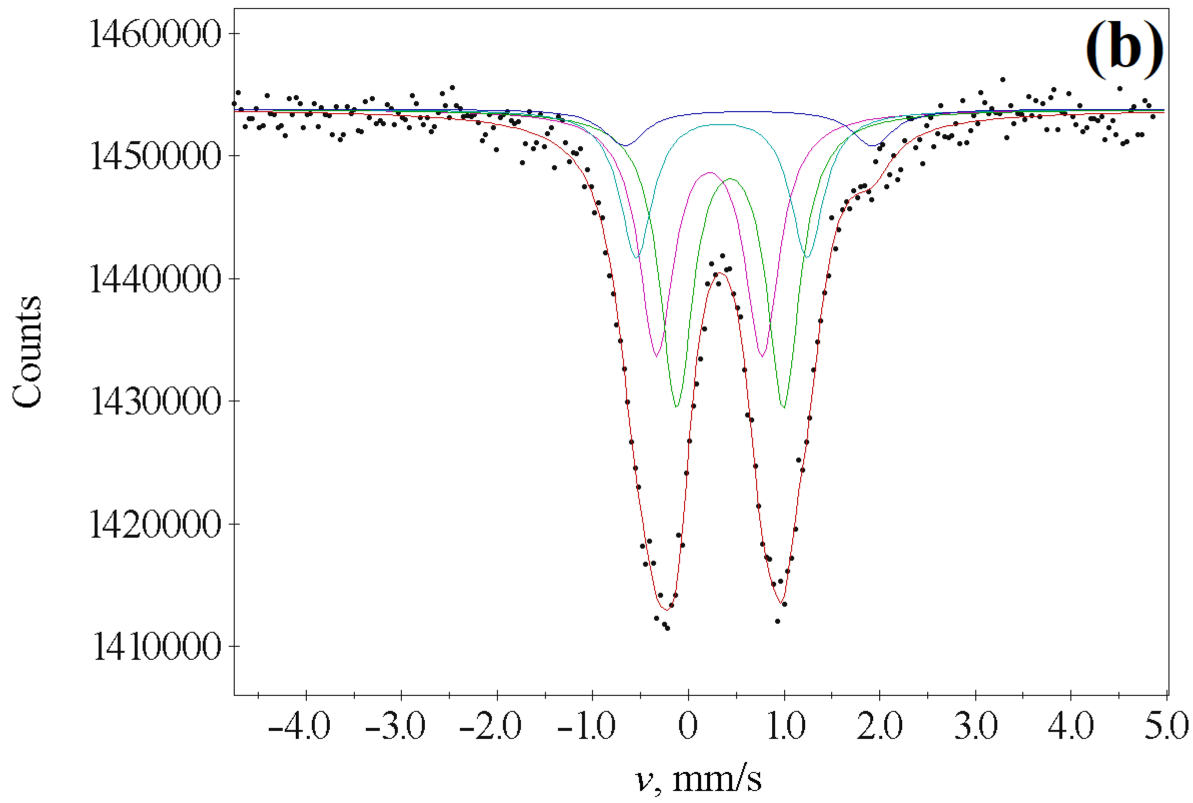

ESI Figure S21 The Mössbauer spectra of the heat treatment products of compound **1** prepared in **(a)** oxidative and **(b)** inert atmosphere at 325 °C**.**

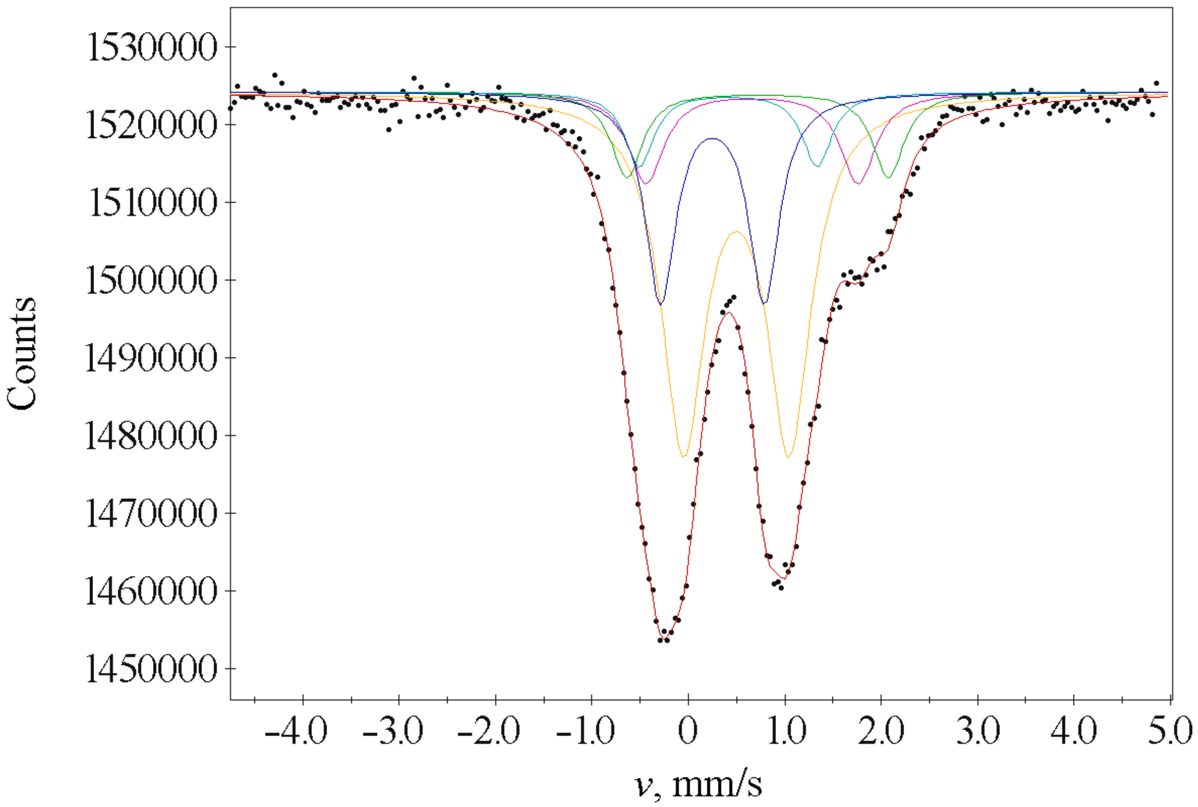

ESI Figure S22 The Mössbauer spectra of the heat treatment products of compound **1** prepared in an inert atmosphere at 400 °C.

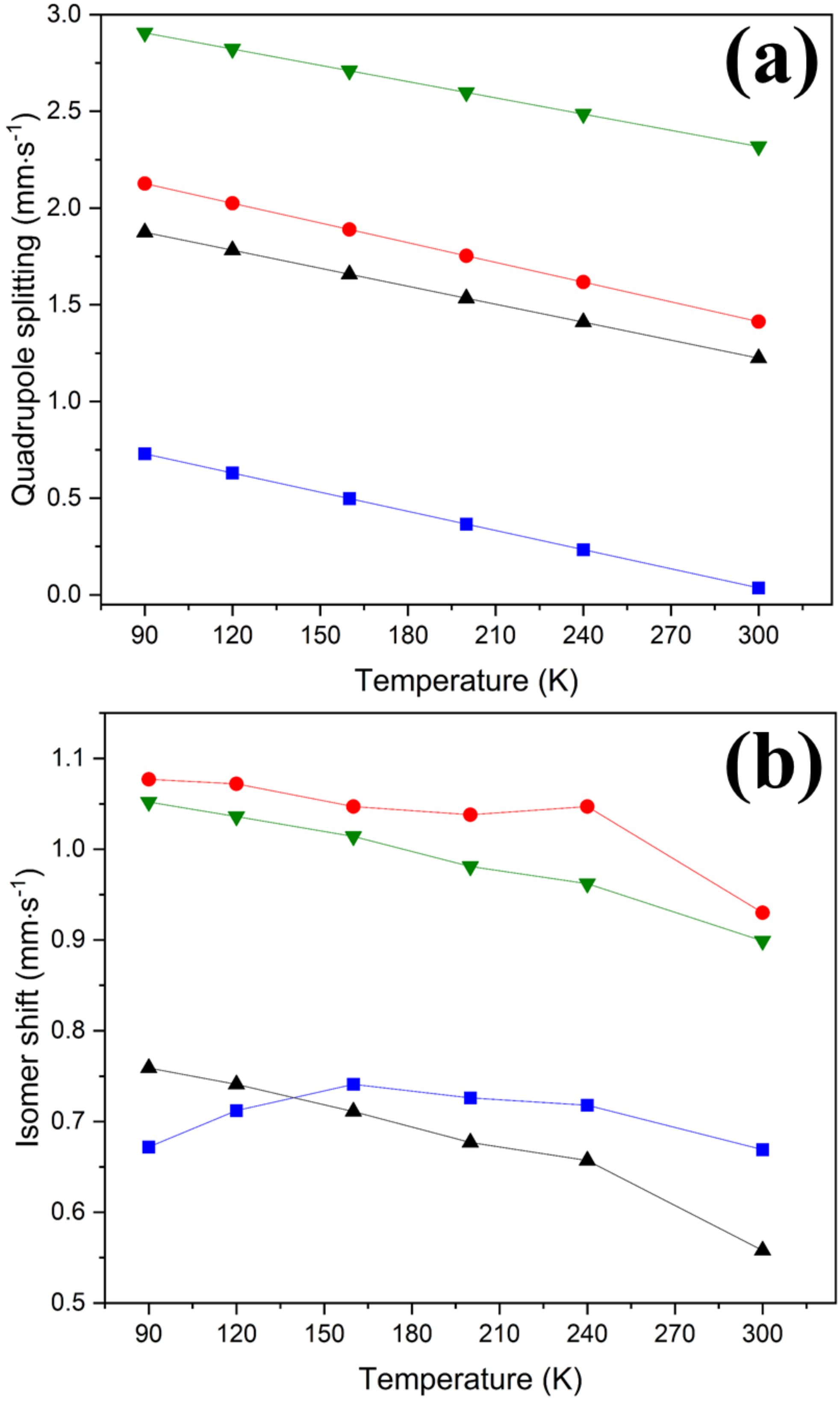

ESI Figure S23 The change (with the temperature) of the quadrupole splitting (QS) and isomer shift (IS) parameters of the decomposition intermediate of compound **1** prepared in an inert atmosphere at 600 °C.

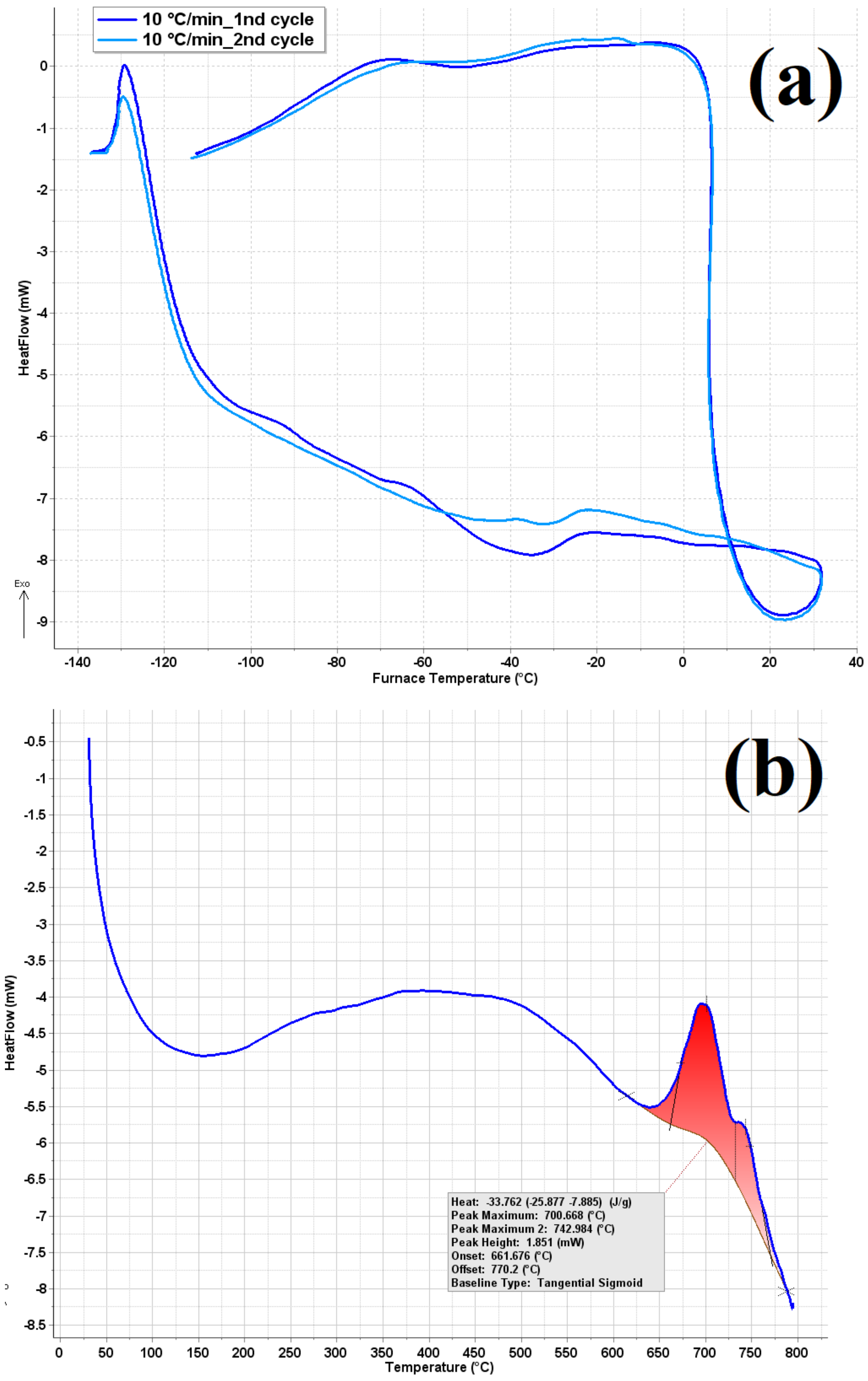

ESI Figure S24 The **(a)** cryo- and **(b)** high-temperature DSC studies of the decomposition intermediate of compound **1** prepared in an inert atmosphere at 600 °C.

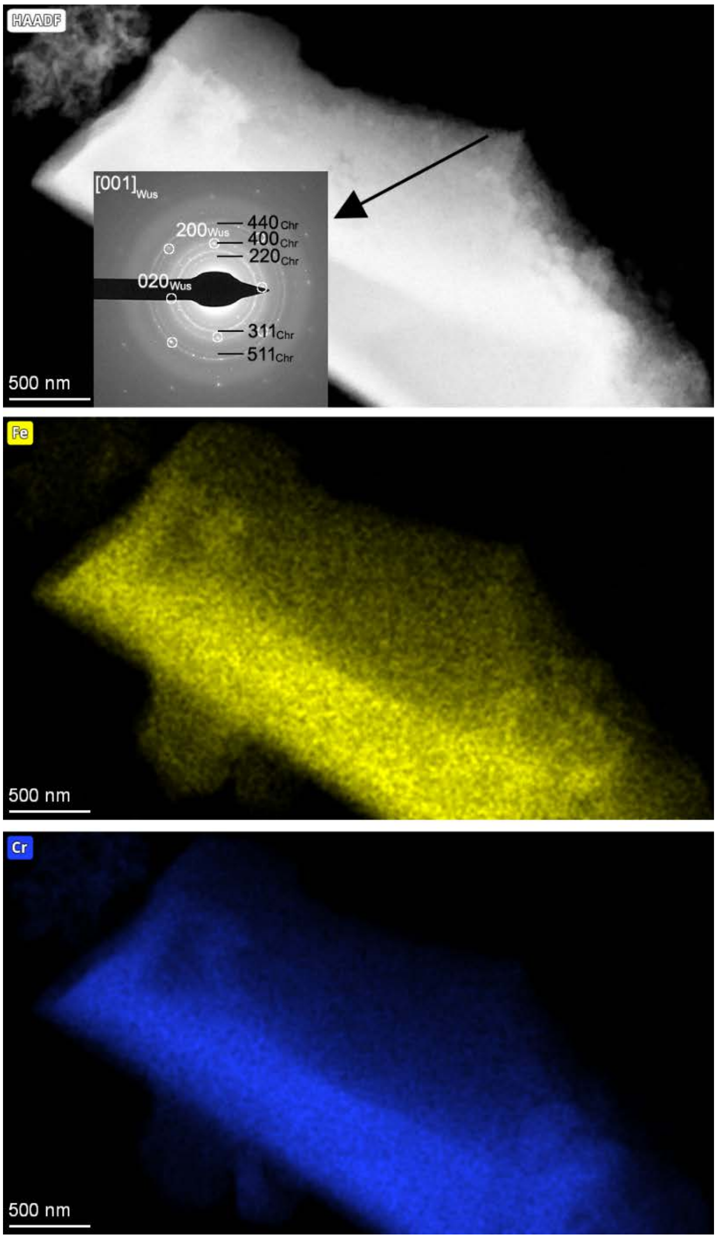

ESI Figure S25 The SEAD and the distribution of the Cr and Fe in the heat treatment product of compound **1** in an inert atmosphere prepared at 800 °C.

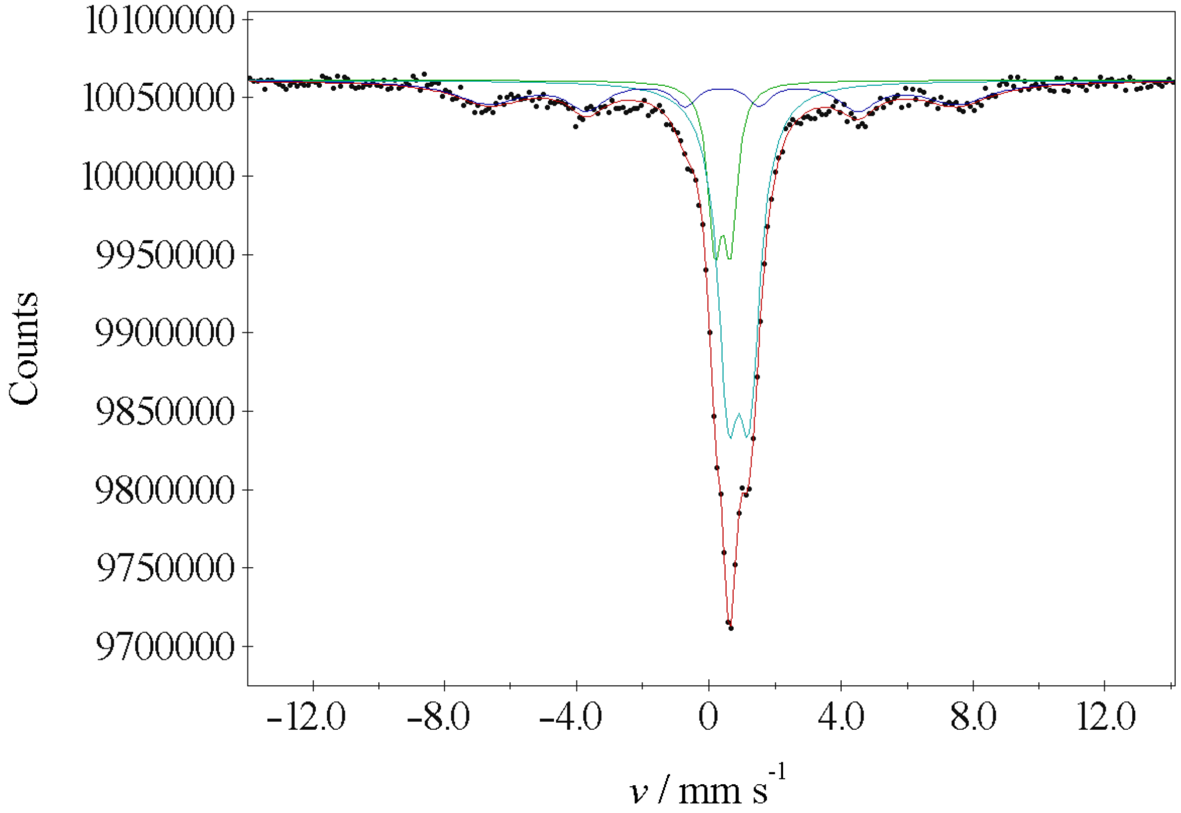

ESI Figure S26 The Mössbauer spectra (measured at RT) of the heat treatment product of compound **1** in an inert atmosphere prepared at 800 °C.

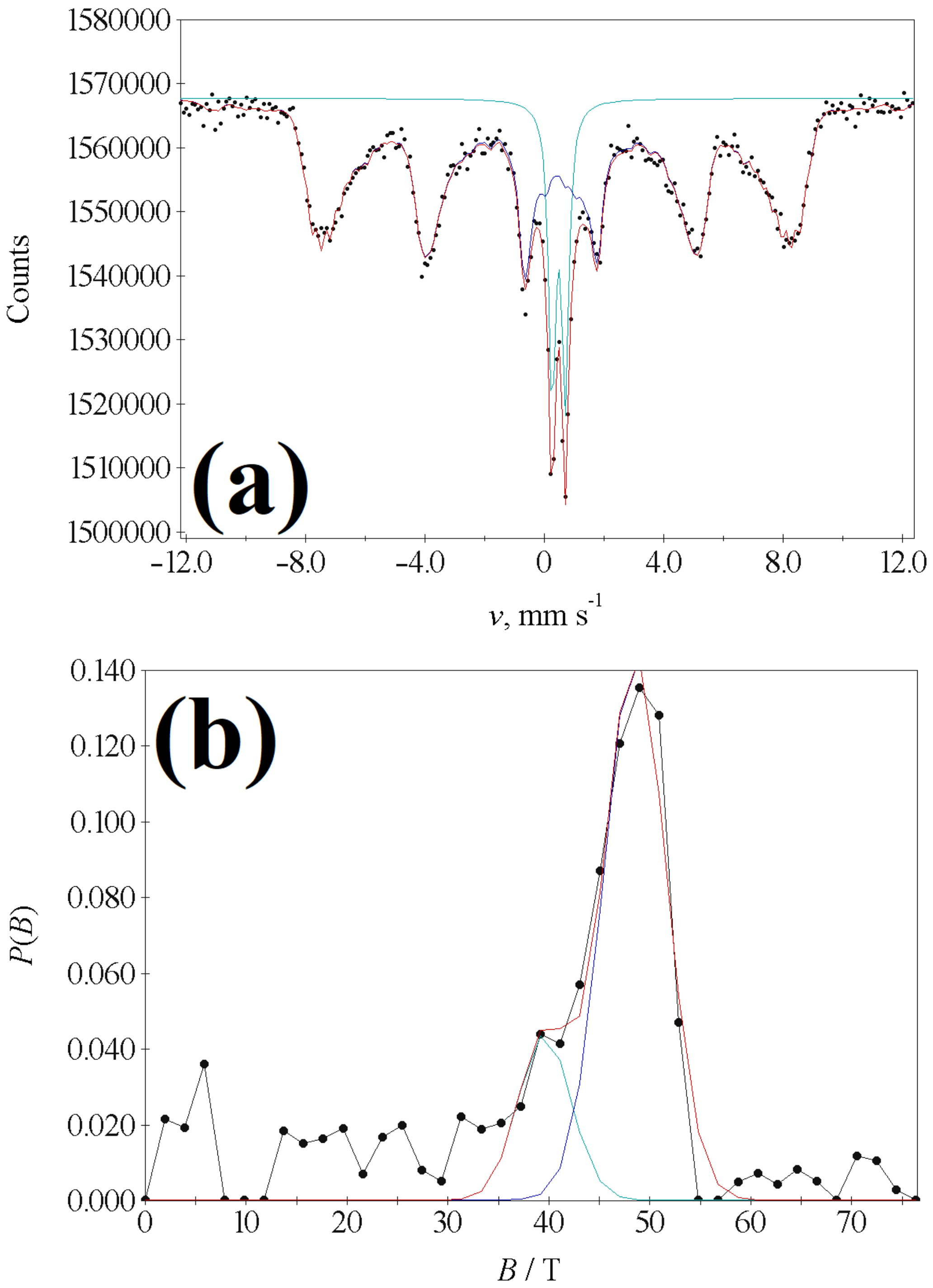

ESI Figure S27 The **(a)** Mössbauer spectra (measured at 90 K) of the heat treatment product of compound **1** in an inert atmosphere prepared at 800 °C. **(b)** The magnetic field distribution evaluation of the sample.

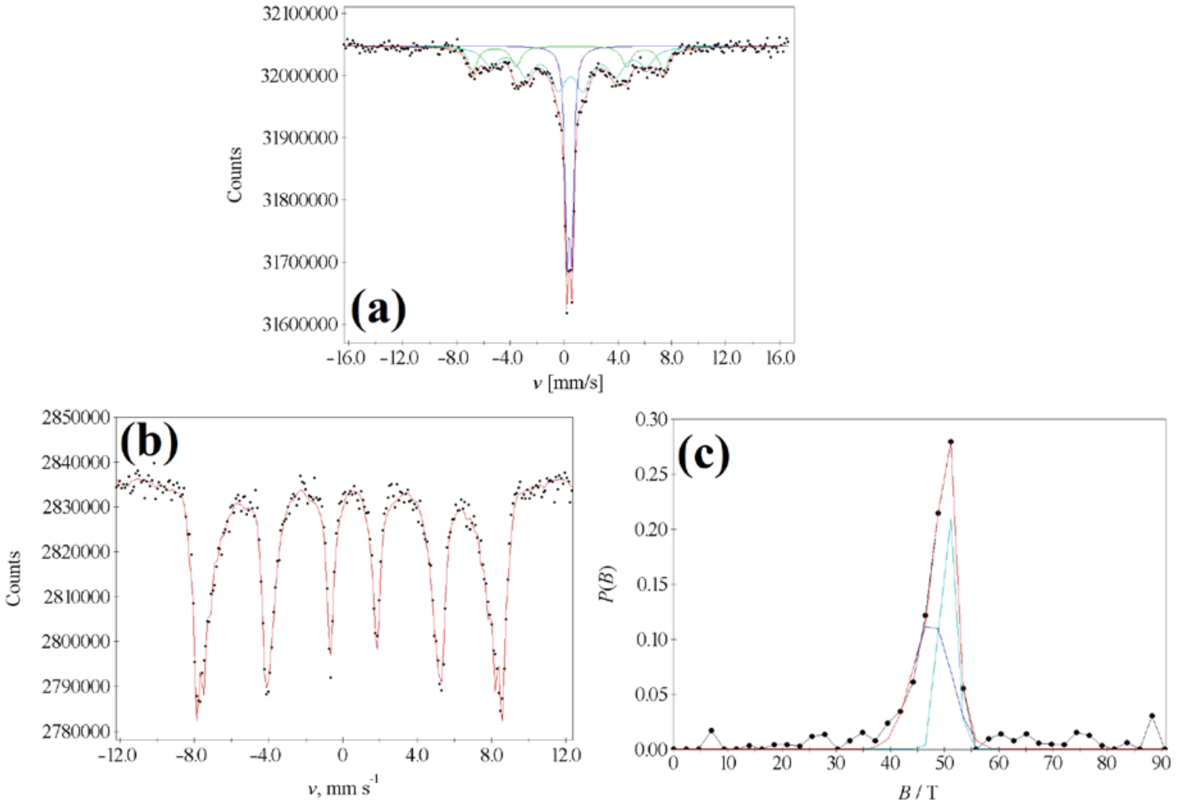

ESI Figure S28 The Mössbauer spectra (measured at **(a)** RT and **(b)** 90 K) of the heat treatment product of compound **1** in an inert atmosphere prepared at 800 °C. **(c)** The evaluation of the magnetic field distribution (at 90 K).

ESI Table S14 The Preliminary result of catalytic activity test of iron-chromium mixed oxides in CO_2 hydrogenation reaction.

Heat treatment condition	Max. Conv.[%]	CO [%]	CH_4 [%]	C_2H_6 [%]	C_3H_8 [%]
200 °C (Air)	61.9 ± 0.2	93.1 ± 0.2	6.8 ± 0.2	0.2 ± 0.1	-
350 °C (Air)	61.3 ± 0.3	91.0 ± 1.0	8.4 ± 0.9	0.2 ± 0.02	-
550 °C (Air)	53.0 ± 1.3	74.5 ± 1.6	24.2 ± 1.1	1.2 ± 0.1	0.1 ± 0.02

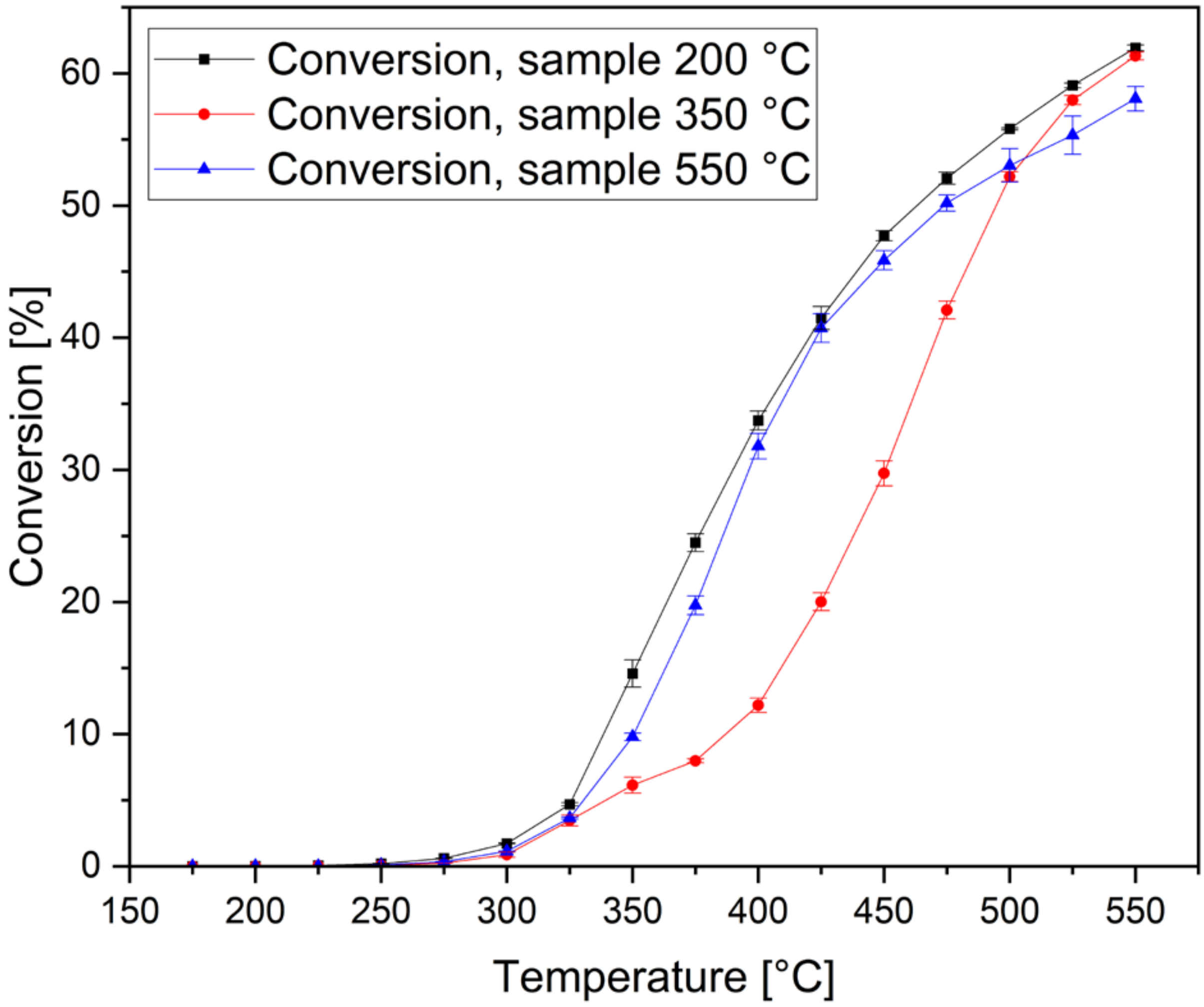

ESI Figure S29 The conversion % of CO_2 in the hydrogenation reaction.

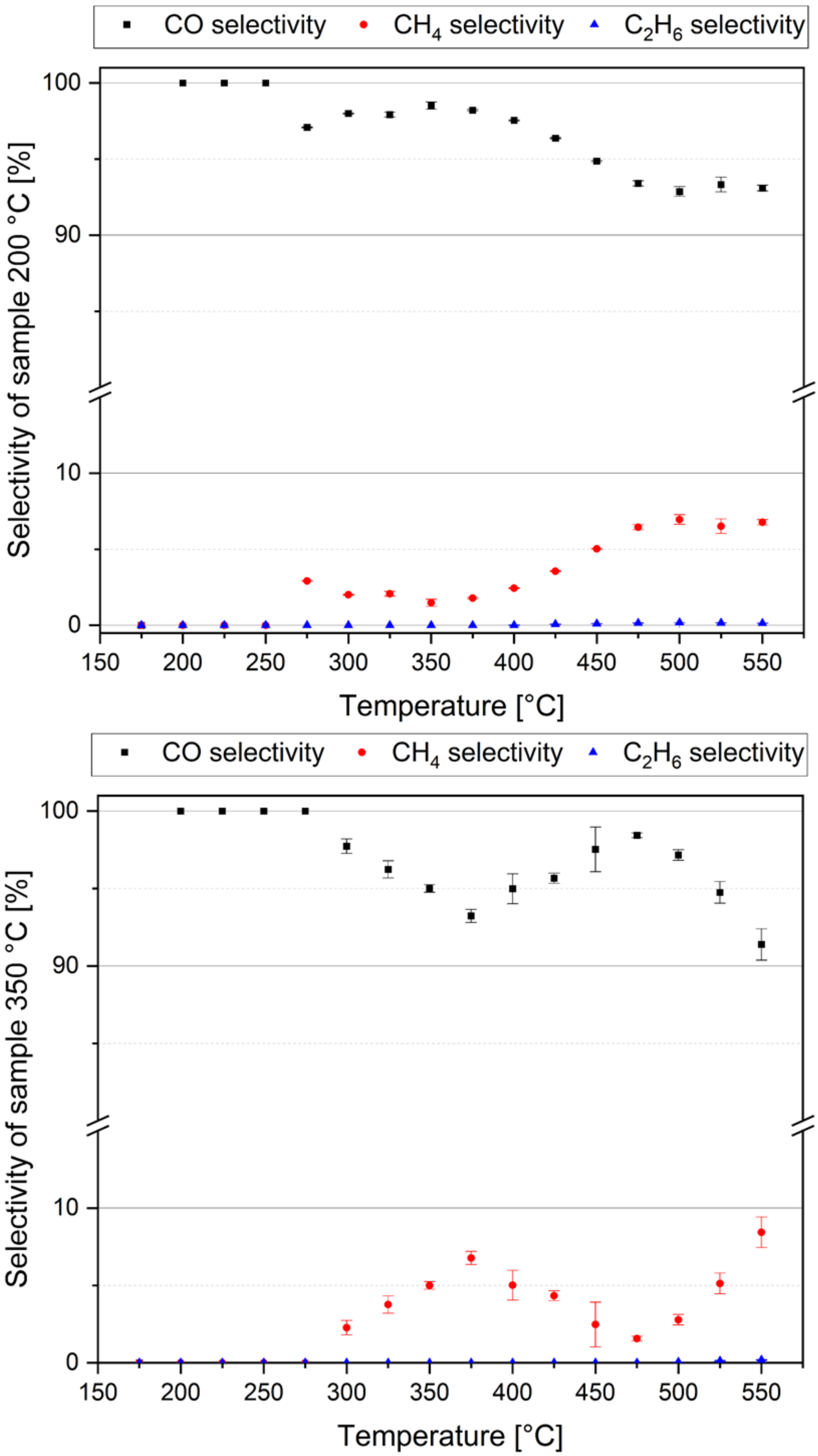

ESI Figure S30 The selectivity of the different catalysts in the CO_2 hydrogenation reaction.
